# Supplementary material for: Molecular Signatures Integral to Natural Reprogramming in the Pigment Epithelium Cells after Retinal Detachment in Pleurodeles waltl
Source: Int J Mol Sci. 2023 Nov 29;24(23):16940. doi: 10.3390/ijms242316940 (PMC10707686; doi:10.3390/ijms242316940)
Supplement: Supplementary file 1 [file ijms-24-16940-s001.zip › Supplementary Materials_Markitantova et al_2023.pdf]

Supplementary Materials for manuscript of

Markitantova et al. “Molecular signatures integral to natural reprogramming in the pigment epithelium cells after retinal detachment in Pleurodeles waltl”

Table S1. Transcripts of differentially expressed genes (DEGs) in RPE during an early stage of retina regeneration in the newt.

NN		logFC	logCPM	P-Value	FDR
1	TRINITY_DN955_c1_g2_i3	19.692	11.559	4.6E-40	1.9E-37
2	TRINITY_DN93_c0_g1_i36	11.567	3.442	9.8E-16	4.6E-14
3	TRINITY_DN93_c0_g1_i41	11.522	3.402	1.3E-15	6.0E-14
4	TRINITY_DN311_c0_g1_i3	10.488	2.379	1.3E-12	3.7E-11
5	TRINITY_DN80_c1_g1_i8	9.799	1.708	1.1E-10	2.6E-09
6	TRINITY_DN31589_c1_g1_i1	9.589	1.493	3.8E-10	8.5E-09
7	TRINITY_DN6480_c1_g1_i8	9.376	1.286	1.4E-09	3.0E-08
8	TRINITY_DN3138_c5_g2_i1	9.342	8.099	3.8E-23	3.3E-21
9	TRINITY_DN137_c0_g1_i1	8.976	0.889	1.6E-08	2.8E-07
10	TRINITY_DN2_c0_g3_i6	8.900	5.480	2.4E-19	1.6E-17
11	TRINITY_DN37667_c0_g1_i9	8.706	0.641	7.3E-08	1.1E-06
12	TRINITY_DN222_c0_g1_i57	8.584	0.543	1.6E-07	2.2E-06
13	TRINITY_DN209344_c0_g1_i2	8.520	0.473	2.1E-07	2.9E-06
14	TRINITY_DN79_c0_g1_i14	8.455	0.401	3.0E-07	3.9E-06
15	TRINITY_DN112_c1_g2_i20	8.382	0.318	4.3E-07	5.4E-06
16	TRINITY_DN474_c1_g1_i8	8.374	0.313	4.3E-07	5.4E-06
17	TRINITY_DN727_c0_g1_i2	8.278	0.245	7.8E-07	9.0E-06
18	TRINITY_DN33040_c1_g1_i2	8.261	0.227	8.9E-07	1.0E-05
19	TRINITY_DN2429_c0_g1_i1	8.197	0.157	1.2E-06	1.3E-05
20	TRINITY_DN160_c0_g1_i1	8.105	0.089	2.1E-06	2.2E-05
21	TRINITY_DN149_c0_g1_i4	8.040	0.030	3.0E-06	2.9E-05
22	TRINITY_DN3098_c0_g1_i22	8.034	-0.001	2.8E-06	2.8E-05
23	TRINITY_DN4697_c0_g1_i9	8.034	-0.005	2.8E-06	2.8E-05
24	TRINITY_DN1818_c4_g1_i1	8.025	-0.004	3.0E-06	2.9E-05
25	TRINITY_DN192_c0_g1_i3	7.963	-0.050	4.1E-06	3.8E-05
26	TRINITY_DN222_c0_g1_i50	7.891	-0.119	6.1E-06	5.4E-05
27	TRINITY_DN14_c1_g1_i12	7.827	-0.190	7.8E-06	6.7E-05
28	TRINITY_DN9741_c0_g1_i5	7.765	-0.219	1.2E-05	9.8E-05
29	TRINITY_DN612_c0_g1_i1	7.550	-0.419	3.3E-05	2.3E-04
30	TRINITY_DN103_c0_g1_i10	7.423	-0.521	6.0E-05	3.7E-04
31	TRINITY_DN11300_c1_g1_i2	7.423	-0.533	5.4E-05	3.4E-04
32	TRINITY_DN4726_c0_g1_i3	7.404	-0.574	5.4E-05	3.4E-04
33	TRINITY_DN2559_c0_g1_i36	7.119	-0.795	2.2E-04	1.1E-03
34	TRINITY_DN29373_c0_g1_i10	6.317	1.453	4.8E-09	9.3E-08
35	TRINITY_DN443_c0_g1_i13	6.060	1.216	2.3E-08	4.1E-07
36	TRINITY_DN757_c0_g1_i2	5.633	0.793	2.7E-07	3.7E-06
37	TRINITY_DN1271_c0_g1_i5	5.593	0.749	3.3E-07	4.2E-06
38	TRINITY_DN148_c0_g1_i26	5.547	0.707	4.2E-07	5.3E-06
39	TRINITY_DN5490_c0_g1_i4	5.544	0.713	4.4E-07	5.4E-06
40	TRINITY_DN10858_c9_g1_i1	5.429	0.604	8.5E-07	9.8E-06
41	TRINITY_DN28_c1_g1_i29	5.285	0.474	1.8E-06	2.0E-05
42	TRINITY_DN3169_c0_g1_i3	5.271	1.345	5.5E-08	8.8E-07

43	TRINITY_DN955_c1_g2_i19	5.263	0.466	2.2E-06	2.3E-05
44	TRINITY_DN23068_c2_g1_i1	5.230	0.414	2.4E-06	2.5E-05
45	TRINITY_DN181_c0_g3_i8	5.217	11.841	3.1E-12	8.7E-11
46	TRINITY_DN1666_c0_g1_i3	5.152	0.353	4.0E-06	3.7E-05
47	TRINITY_DN30_c0_g1_i23	5.116	0.312	4.5E-06	4.2E-05
48	TRINITY_DN11_c0_g1_i10	4.978	0.189	9.7E-06	8.2E-05
49	TRINITY_DN4607_c0_g1_i1	4.842	0.054	1.8E-05	1.4E-04
50	TRINITY_DN955_c1_g2_i9	4.714	3.158	3.2E-09	6.4E-08
51	TRINITY_DN352_c0_g3_i2	4.647	-0.128	4.8E-05	3.1E-04
52	TRINITY_DN10266_c0_g1_i10	4.565	1.910	6.0E-08	9.4E-07
53	TRINITY_DN5490_c0_g1_i6	4.193	0.319	2.1E-05	1.6E-04
54	TRINITY_DN11_c0_g1_i17	4.189	0.845	3.9E-06	3.7E-05
55	TRINITY_DN181_c0_g3_i6	4.186	5.546	4.8E-09	9.3E-08
56	TRINITY_DN113_c0_g1_i31	4.159	0.290	2.5E-05	1.8E-04
57	TRINITY_DN17_c0_g1_i2	4.140	0.271	2.8E-05	2.0E-04
58	TRINITY_DN112_c1_g2_i33	4.139	7.964	4.2E-09	8.4E-08
59	TRINITY_DN260_c0_g2_i1	4.095	2.109	2.1E-07	2.9E-06
60	TRINITY_DN11_c0_g1_i8	3.910	0.972	6.1E-06	5.4E-05
61	TRINITY_DN4841_c0_g4_i1	3.849	0.920	8.5E-06	7.3E-05
62	TRINITY_DN5567_c0_g1_i14	3.779	-0.050	1.7E-04	8.8E-04
63	TRINITY_DN2556_c0_g1_i10	3.771	0.838	1.3E-05	1.0E-04
64	TRINITY_DN7478_c0_g1_i1	3.726	-0.078	2.2E-04	1.1E-03
65	TRINITY_DN226077_c0_g1_i1	3.711	1.733	2.0E-06	2.1E-05
66	TRINITY_DN1393_c0_g1_i1	3.711	-0.126	2.2E-04	1.1E-03
67	TRINITY_DN489_c0_g1_i18	3.703	0.775	1.9E-05	1.4E-04
68	TRINITY_DN53300_c0_g2_i6	3.693	-0.123	2.6E-04	1.3E-03
69	TRINITY_DN5087_c1_g4_i1	3.681	1.055	9.7E-06	8.2E-05
70	TRINITY_DN82_c0_g1_i4	3.635	5.257	1.5E-07	2.1E-06
71	TRINITY_DN11263_c0_g1_i10	3.588	4.096	3.0E-07	3.9E-06
72	TRINITY_DN443_c0_g1_i6	3.588	0.302	9.5E-05	5.5E-04
73	TRINITY_DN9421_c0_g1_i1	3.537	-0.279	4.9E-04	2.2E-03
74	TRINITY_DN129_c0_g5_i1	3.517	0.899	2.3E-05	1.7E-04
75	TRINITY_DN15513_c7_g1_i1	3.473	2.481	2.4E-06	2.5E-05
76	TRINITY_DN138613_c1_g1_i1	3.449	0.564	7.2E-05	4.3E-04
77	TRINITY_DN59306_c0_g3_i1	3.422	2.744	2.1E-06	2.2E-05
78	TRINITY_DN10760_c0_g1_i8	3.414	5.548	4.9E-07	6.0E-06
79	TRINITY_DN394_c0_g1_i14	3.395	0.112	2.3E-04	1.2E-03
80	TRINITY_DN16632_c4_g1_i1	3.390	5.258	6.0E-07	7.2E-06
81	TRINITY_DN3404_c1_g1_i3	3.388	0.480	9.4E-05	5.5E-04
82	TRINITY_DN5090_c6_g1_i1	3.387	2.641	2.9E-06	2.9E-05
83	TRINITY_DN4219_c0_g1_i1	3.386	0.474	9.4E-05	5.5E-04
84	TRINITY_DN30721_c0_g4_i1	3.385	0.786	4.8E-05	3.1E-04
85	TRINITY_DN210_c0_g1_i1	3.364	0.452	1.0E-04	5.9E-04
86	TRINITY_DN379_c0_g1_i1	3.350	0.446	1.1E-04	6.2E-04
87	TRINITY_DN181_c0_g3_i1	3.336	8.086	6.2E-07	7.3E-06
88	TRINITY_DN594_c0_g1_i2	3.310	0.954	4.0E-05	2.7E-04
89	TRINITY_DN516_c0_g2_i4	3.267	-0.005	4.2E-04	1.9E-03
90	TRINITY_DN1101_c0_g1_i2	3.249	-0.020	4.5E-04	2.1E-03
91	TRINITY_DN82_c0_g1_i7	3.208	2.038	1.5E-05	1.2E-04
92	TRINITY_DN3153_c0_g1_i2	3.188	0.312	2.6E-04	1.3E-03
93	TRINITY_DN119_c0_g1_i15	3.162	-0.612	2.2E-03	7.9E-03
94	TRINITY_DN15_c0_g2_i6	3.146	0.260	3.1E-04	1.5E-03
95	TRINITY_DN148_c0_g1_i13	3.146	0.563	1.6E-04	8.5E-04
96	TRINITY_DN80_c1_g1_i33	3.143	3.572	4.6E-06	4.3E-05
97	TRINITY_DN82_c0_g1_i2	3.050	2.459	1.7E-05	1.3E-04

98	TRINITY_DN211081_c0_g1_i1	3.039	-0.210	1.1E-03	4.3E-03
99	TRINITY_DN1875_c0_g2_i10	3.037	0.704	1.7E-04	8.9E-04
100	TRINITY_DN102_c0_g1_i1	3.022	2.292	2.3E-05	1.7E-04
101	TRINITY_DN11661_c0_g2_i1	3.019	0.444	2.9E-04	1.4E-03
102	TRINITY_DN1536_c0_g1_i11	2.993	0.131	6.0E-04	2.6E-03
103	TRINITY_DN270767_c0_g1_i1	2.989	6.696	5.4E-06	4.9E-05
104	TRINITY_DN535_c0_g1_i3	2.976	1.819	5.3E-05	3.4E-04
105	TRINITY_DN2_c0_g4_i1	2.945	0.615	2.5E-04	1.3E-03
106	TRINITY_DN1180_c0_g2_i1	2.933	-0.306	1.7E-03	6.4E-03
107	TRINITY_DN4702_c0_g1_i1	2.930	2.944	2.3E-05	1.7E-04
108	TRINITY_DN939_c0_g1_i5	2.928	1.964	5.3E-05	3.4E-04
109	TRINITY_DN474_c1_g1_i9	2.924	0.374	4.8E-04	2.1E-03
110	TRINITY_DN112_c1_g2_i22	2.921	1.165	2.0E-04	1.0E-03
111	TRINITY_DN123_c0_g1_i4	2.896	2.019	5.7E-05	3.5E-04
112	TRINITY_DN1708_c0_g2_i2	2.889	-0.315	2.4E-03	8.3E-03
113	TRINITY_DN137_c0_g1_i2	2.874	1.909	7.1E-05	4.3E-04
114	TRINITY_DN2823_c0_g1_i4	2.856	0.022	1.2E-03	4.5E-03
115	TRINITY_DN955_c28_g1_i1	2.843	0.747	2.9E-04	1.4E-03
116	TRINITY_DN93_c0_g1_i2	2.841	1.239	2.3E-04	1.2E-03
117	TRINITY_DN179_c0_g1_i21	2.831	3.207	2.9E-05	2.1E-04
118	TRINITY_DN1479_c0_g2_i3	2.805	2.584	4.5E-05	3.0E-04
119	TRINITY_DN172904_c0_g1_i1	2.790	1.438	2.0E-04	1.0E-03
120	TRINITY_DN65289_c0_g1_i1	2.777	-0.057	1.6E-03	6.1E-03
121	TRINITY_DN92_c0_g1_i8	2.775	0.220	9.0E-04	3.6E-03
122	TRINITY_DN3716_c1_g5_i1	2.767	1.149	3.2E-04	1.5E-03
123	TRINITY_DN40630_c0_g1_i2	2.765	4.407	2.7E-05	2.0E-04
124	TRINITY_DN19569_c0_g1_i7	2.721	4.788	3.1E-05	2.2E-04
125	TRINITY_DN300822_c0_g1_i1	2.720	2.216	9.5E-05	5.5E-04
126	TRINITY_DN40630_c0_g1_i3	2.719	4.765	3.1E-05	2.2E-04
127	TRINITY_DN265_c0_g1_i1	2.702	-0.479	4.7E-03	1.4E-02
128	TRINITY_DN19569_c0_g1_i2	2.701	4.003	4.3E-05	2.9E-04
129	TRINITY_DN6651_c0_g1_i11	2.693	1.346	3.2E-04	1.5E-03
130	TRINITY_DN52257_c0_g1_i8	2.663	0.567	6.7E-04	2.8E-03
131	TRINITY_DN10266_c0_g1_i7	2.654	2.446	1.0E-04	5.9E-04
132	TRINITY_DN6230_c6_g1_i1	2.648	7.109	3.7E-05	2.6E-04
133	TRINITY_DN12229_c1_g1_i5	2.644	0.107	1.6E-03	6.1E-03
134	TRINITY_DN181_c1_g1_i1	2.638	7.357	3.9E-05	2.6E-04
135	TRINITY_DN70900_c0_g3_i1	2.624	-0.207	2.9E-03	9.8E-03
136	TRINITY_DN9754_c0_g3_i3	2.618	0.528	8.5E-04	3.5E-03
137	TRINITY_DN6763_c2_g6_i2	2.600	0.529	9.4E-04	3.7E-03
138	TRINITY_DN570_c0_g2_i2	2.563	0.043	2.3E-03	7.9E-03
139	TRINITY_DN10_c0_g1_i1	2.563	0.475	1.1E-03	4.3E-03
140	TRINITY_DN7379_c0_g1_i5	2.556	-0.259	3.9E-03	1.2E-02
141	TRINITY_DN149_c0_g1_i5	2.546	-0.257	4.2E-03	1.3E-02
142	TRINITY_DN28_c1_g1_i9	2.543	0.652	9.2E-04	3.7E-03
143	TRINITY_DN11067_c0_g3_i4	2.523	-0.271	4.6E-03	1.4E-02
144	TRINITY_DN4745_c1_g1_i8	2.481	-0.031	3.3E-03	1.1E-02
145	TRINITY_DN20576_c0_g2_i2	2.475	4.544	1.2E-04	6.7E-04
146	TRINITY_DN290_c0_g1_i15	2.474	1.702	4.6E-04	2.1E-03
147	TRINITY_DN8690_c1_g1_i2	2.463	0.181	2.3E-03	8.1E-03
148	TRINITY_DN16495_c0_g1_i1	2.462	0.189	2.3E-03	8.1E-03
149	TRINITY_DN18248_c0_g2_i5	2.401	-0.399	7.3E-03	2.1E-02
150	TRINITY_DN15937_c0_g1_i3	2.399	0.336	2.2E-03	7.9E-03
151	TRINITY_DN1779_c0_g1_i15	2.389	-0.105	5.0E-03	1.5E-02
152	TRINITY_DN261_c0_g1_i2	2.363	0.785	2.2E-03	7.9E-03

153	TRINITY_DN21_c0_g1_i4	2.357	0.091	3.7E-03	1.2E-02
154	TRINITY_DN1460_c0_g1_i1	2.349	8.214	1.9E-04	9.9E-04
155	TRINITY_DN2263_c0_g1_i4	2.345	0.084	4.0E-03	1.3E-02
156	TRINITY_DN179_c0_g1_i11	2.337	5.922	2.2E-04	1.1E-03
157	TRINITY_DN7347_c1_g1_i1	2.334	3.425	3.3E-04	1.5E-03
158	TRINITY_DN1667_c11_g1_i1	2.327	4.280	2.8E-04	1.4E-03
159	TRINITY_DN165315_c2_g1_i1	2.322	12.173	2.1E-04	1.1E-03
160	TRINITY_DN42741_c0_g1_i2	2.311	0.253	3.2E-03	1.1E-02
161	TRINITY_DN2_c1_g1_i1	2.306	1.102	1.8E-03	6.5E-03
162	TRINITY_DN10760_c0_g1_i2	2.273	3.506	4.1E-04	1.9E-03
163	TRINITY_DN955_c1_g2_i25	2.271	7.456	2.9E-04	1.4E-03
164	TRINITY_DN1625_c0_g2_i1	2.264	-0.224	7.6E-03	2.2E-02
165	TRINITY_DN2340_c0_g1_i1	2.263	0.951	2.5E-03	8.6E-03
166	TRINITY_DN8380_c0_g1_i6	2.253	-0.500	1.3E-02	3.4E-02
167	TRINITY_DN1189_c0_g1_i2	2.249	-0.514	1.3E-02	3.4E-02
168	TRINITY_DN6651_c0_g1_i8	2.226	1.627	1.3E-03	5.1E-03
169	TRINITY_DN908_c0_g1_i6	2.219	0.660	4.1E-03	1.3E-02
170	TRINITY_DN3010_c0_g5_i3	2.217	1.547	1.5E-03	5.7E-03
171	TRINITY_DN9729_c0_g1_i11	2.214	0.906	3.1E-03	1.0E-02
172	TRINITY_DN28_c0_g3_i1	2.206	0.508	5.3E-03	1.6E-02
173	TRINITY_DN776_c0_g1_i10	2.206	-0.028	7.0E-03	2.0E-02
174	TRINITY_DN56112_c0_g1_i1	2.206	2.322	1.0E-03	4.0E-03
175	TRINITY_DN2850_c0_g1_i17	2.204	8.015	4.1E-04	1.9E-03
176	TRINITY_DN93_c0_g1_i44	2.186	3.365	6.6E-04	2.8E-03
177	TRINITY_DN101_c0_g1_i6	2.184	-0.557	1.7E-02	4.1E-02
178	TRINITY_DN5308_c0_g2_i1	2.168	4.792	5.8E-04	2.5E-03
179	TRINITY_DN39414_c0_g1_i1	2.156	0.291	4.8E-03	1.5E-02
180	TRINITY_DN5330_c0_g1_i4	2.138	1.802	1.6E-03	6.1E-03
181	TRINITY_DN27378_c1_g1_i1	2.130	2.369	1.3E-03	5.1E-03
182	TRINITY_DN1245_c2_g1_i4	2.127	0.580	6.2E-03	1.8E-02
183	TRINITY_DN7688_c0_g3_i1	2.121	0.569	6.2E-03	1.8E-02
184	TRINITY_DN179_c0_g1_i7	2.117	6.131	6.7E-04	2.8E-03
185	TRINITY_DN955_c1_g2_i33	2.060	8.765	8.6E-04	3.5E-03
186	TRINITY_DN955_c1_g2_i8	2.060	8.765	8.6E-04	3.5E-03
187	TRINITY_DN83939_c0_g1_i1	2.057	0.208	7.4E-03	2.1E-02
188	TRINITY_DN381_c0_g1_i4	2.043	0.355	6.5E-03	1.9E-02
189	TRINITY_DN312_c0_g1_i11	2.036	-0.397	1.9E-02	4.5E-02
190	TRINITY_DN3338_c0_g1_i9	2.034	1.305	3.8E-03	1.2E-02
191	TRINITY_DN131_c0_g1_i26	2.031	2.713	1.7E-03	6.2E-03
192	TRINITY_DN11488_c7_g1_i1	2.030	0.184	8.2E-03	2.3E-02
193	TRINITY_DN58_c0_g1_i10	2.029	1.758	2.6E-03	8.9E-03
194	TRINITY_DN13791_c0_g1_i5	2.002	1.672	3.0E-03	1.0E-02
195	TRINITY_DN311_c0_g1_i6	1.997	2.771	1.9E-03	6.9E-03
196	TRINITY_DN4841_c0_g2_i1	1.989	-0.026	1.2E-02	3.1E-02
197	TRINITY_DN3519_c2_g1_i1	1.976	1.648	3.4E-03	1.1E-02
198	TRINITY_DN143_c0_g1_i2	1.968	0.434	1.2E-02	3.1E-02
199	TRINITY_DN1479_c0_g1_i2	1.968	0.563	1.0E-02	2.7E-02
200	TRINITY_DN1875_c0_g2_i13	1.953	0.776	8.3E-03	2.3E-02
201	TRINITY_DN11263_c0_g1_i15	1.952	3.271	2.0E-03	7.4E-03
202	TRINITY_DN9706_c0_g1_i1	1.947	0.977	7.1E-03	2.1E-02
203	TRINITY_DN28_c1_g1_i10	1.942	1.153	6.2E-03	1.8E-02
204	TRINITY_DN37209_c0_g1_i2	1.930	0.120	1.3E-02	3.4E-02
205	TRINITY_DN35157_c4_g1_i1	1.922	4.260	2.0E-03	7.4E-03
206	TRINITY_DN24297_c1_g3_i2	1.916	2.318	3.3E-03	1.1E-02
207	TRINITY_DN351_c0_g1_i1	1.908	1.774	4.3E-03	1.3E-02

208	TRINITY_DN131_c0_g1_i7	1.896	2.369	3.5E-03	1.1E-02
209	TRINITY_DN28_c1_g1_i14	1.883	0.387	1.7E-02	4.2E-02
210	TRINITY_DN895_c1_g1_i14	1.876	0.067	1.5E-02	3.9E-02
211	TRINITY_DN1411_c0_g1_i12	1.867	0.202	1.3E-02	3.4E-02
212	TRINITY_DN8_c17_g1_i13	1.861	7.264	2.3E-03	8.1E-03
213	TRINITY_DN1929_c7_g1_i1	1.858	5.119	2.5E-03	8.7E-03
214	TRINITY_DN6671_c0_g1_i1	1.858	0.990	9.2E-03	2.5E-02
215	TRINITY_DN2224_c1_g2_i1	1.852	8.068	2.4E-03	8.4E-03
216	TRINITY_DN160_c0_g1_i8	1.846	0.044	1.7E-02	4.2E-02
217	TRINITY_DN224_c0_g1_i14	1.843	0.962	9.5E-03	2.6E-02
218	TRINITY_DN394_c0_g1_i12	1.807	0.159	1.6E-02	4.1E-02
219	TRINITY_DN0_c3_g1_i27	1.797	13.186	3.1E-03	1.0E-02
220	TRINITY_DN23_c0_g2_i3	1.791	1.655	7.0E-03	2.0E-02
221	TRINITY_DN311_c0_g1_i10	1.780	4.171	4.0E-03	1.3E-02
222	TRINITY_DN1245_c10_g1_i2	1.770	0.129	1.9E-02	4.7E-02
223	TRINITY_DN0_c3_g2_i1	1.761	0.989	1.3E-02	3.4E-02
224	TRINITY_DN179_c0_g1_i15	1.758	0.600	1.7E-02	4.2E-02
225	TRINITY_DN43236_c0_g1_i8	1.757	2.798	5.7E-03	1.7E-02
226	TRINITY_DN955_c1_g2_i6	1.757	10.108	3.8E-03	1.2E-02
227	TRINITY_DN34362_c4_g1_i1	1.748	6.122	4.1E-03	1.3E-02
228	TRINITY_DN46193_c0_g1_i1	1.747	1.977	7.9E-03	2.3E-02
229	TRINITY_DN2189_c0_g4_i10	1.741	1.799	7.7E-03	2.2E-02
230	TRINITY_DN396_c0_g3_i2	1.731	0.592	2.0E-02	4.8E-02
231	TRINITY_DN10266_c0_g1_i1	1.729	0.709	1.9E-02	4.7E-02
232	TRINITY_DN58410_c0_g1_i1	1.714	5.284	4.9E-03	1.5E-02
233	TRINITY_DN24357_c0_g1_i9	1.704	0.757	1.9E-02	4.5E-02
234	TRINITY_DN13354_c0_g2_i4	1.642	1.415	1.4E-02	3.5E-02
235	TRINITY_DN28_c1_g1_i4	1.633	1.669	1.3E-02	3.3E-02
236	TRINITY_DN30_c0_g1_i34	1.631	1.157	1.7E-02	4.2E-02
237	TRINITY_DN40630_c0_g1_i1	1.596	5.617	8.4E-03	2.4E-02
238	TRINITY_DN1044_c0_g2_i1	1.565	2.143	1.5E-02	3.7E-02
239	TRINITY_DN36611_c0_g1_i1	1.557	3.785	1.1E-02	3.0E-02
240	TRINITY_DN312_c0_g1_i4	1.555	1.933	1.7E-02	4.2E-02
241	TRINITY_DN93_c0_g1_i14	1.552	1.215	2.1E-02	5.0E-02
242	TRINITY_DN10728_c0_g1_i9	1.547	1.799	1.8E-02	4.5E-02
243	TRINITY_DN64_c0_g2_i20	1.533	3.565	1.3E-02	3.4E-02
244	TRINITY_DN4270_c0_g1_i6	1.524	2.827	1.4E-02	3.7E-02
245	TRINITY_DN3049_c0_g1_i1	1.523	2.721	1.5E-02	3.7E-02
246	TRINITY_DN4564_c9_g1_i1	1.482	6.102	1.4E-02	3.5E-02
247	TRINITY_DN8_c0_g1_i3	1.470	2.119	2.1E-02	5.0E-02
248	TRINITY_DN8_c0_g1_i27	1.465	4.365	1.6E-02	3.9E-02
249	TRINITY_DN19480_c0_g4_i1	1.459	6.450	1.5E-02	3.7E-02
250	TRINITY_DN179_c0_g1_i26	1.390	4.689	2.1E-02	5.0E-02
251	TRINITY_DN19591_c0_g2_i14	-1.134	8.841	2.0E-02	4.8E-02
252	TRINITY_DN129_c1_g2_i1	-1.137	8.509	2.0E-02	4.8E-02
253	TRINITY_DN8433_c0_g3_i1	-1.140	6.650	2.0E-02	4.8E-02
254	TRINITY_DN21693_c7_g1_i1	-1.162	5.911	1.8E-02	4.3E-02
255	TRINITY_DN0_c3_g1_i28	-1.177	3.635	1.8E-02	4.3E-02
256	TRINITY_DN210791_c0_g1_i1	-1.193	3.384	1.6E-02	4.0E-02
257	TRINITY_DN2_c6_g1_i3	-1.199	2.483	1.7E-02	4.2E-02
258	TRINITY_DN112_c46_g1_i1	-1.201	9.725	1.4E-02	3.5E-02
259	TRINITY_DN2102_c0_g2_i2	-1.206	2.729	1.6E-02	4.0E-02
260	TRINITY_DN0_c9_g4_i1	-1.210	10.277	1.3E-02	3.4E-02
261	TRINITY_DN28_c0_g1_i36	-1.223	1.512	1.8E-02	4.4E-02
262	TRINITY_DN1441_c0_g1_i11	-1.234	3.922	1.3E-02	3.3E-02

263	TRINITY_DN5_c0_g1_i1	-1.234	1.700	1.6E-02	4.1E-02
264	TRINITY_DN26986_c0_g2_i3	-1.239	1.163	1.9E-02	4.7E-02
265	TRINITY_DN335_c12_g1_i1	-1.239	3.706	1.2E-02	3.3E-02
266	TRINITY_DN31978_c2_g1_i1	-1.246	4.303	1.1E-02	3.1E-02
267	TRINITY_DN16127_c0_g1_i12	-1.246	4.643	1.1E-02	3.0E-02
268	TRINITY_DN961_c0_g1_i3	-1.247	1.400	1.7E-02	4.2E-02
269	TRINITY_DN2_c0_g2_i2	-1.251	0.760	1.9E-02	4.7E-02
270	TRINITY_DN8_c0_g1_i2	-1.256	2.445	1.3E-02	3.3E-02
271	TRINITY_DN981_c0_g2_i1	-1.266	2.019	1.3E-02	3.4E-02
272	TRINITY_DN7607_c0_g1_i1	-1.268	1.208	1.6E-02	4.0E-02
273	TRINITY_DN475_c0_g1_i13	-1.271	2.002	1.2E-02	3.2E-02
274	TRINITY_DN108_c2_g3_i2	-1.279	4.701	9.3E-03	2.6E-02
275	TRINITY_DN125068_c0_g1_i1	-1.282	2.806	1.0E-02	2.7E-02
276	TRINITY_DN28_c5_g1_i16	-1.291	3.466	9.0E-03	2.5E-02
277	TRINITY_DN28_c0_g2_i5	-1.300	2.335	9.8E-03	2.7E-02
278	TRINITY_DN85_c1_g1_i12	-1.305	1.116	1.3E-02	3.4E-02
279	TRINITY_DN2907_c0_g1_i20	-1.307	1.306	1.2E-02	3.2E-02
280	TRINITY_DN1909_c0_g1_i1	-1.309	2.744	9.0E-03	2.5E-02
281	TRINITY_DN353_c0_g1_i2	-1.322	0.642	1.6E-02	4.0E-02
282	TRINITY_DN10121_c0_g1_i15	-1.325	-0.045	2.0E-02	4.8E-02
283	TRINITY_DN2442_c0_g1_i3	-1.332	0.791	1.3E-02	3.4E-02
284	TRINITY_DN8347_c2_g2_i2	-1.341	8.328	5.9E-03	1.7E-02
285	TRINITY_DN28_c0_g1_i16	-1.342	0.636	1.2E-02	3.2E-02
286	TRINITY_DN9462_c5_g1_i1	-1.342	7.835	5.8E-03	1.7E-02
287	TRINITY_DN2907_c0_g1_i23	-1.355	2.318	7.2E-03	2.1E-02
288	TRINITY_DN28_c0_g1_i7	-1.355	0.740	1.2E-02	3.3E-02
289	TRINITY_DN8690_c4_g1_i1	-1.365	2.367	6.8E-03	2.0E-02
290	TRINITY_DN384_c0_g1_i20	-1.370	1.552	8.2E-03	2.3E-02
291	TRINITY_DN28_c2_g1_i10	-1.373	0.253	1.3E-02	3.4E-02
292	TRINITY_DN246_c0_g1_i20	-1.373	1.381	8.9E-03	2.5E-02
293	TRINITY_DN850_c0_g1_i16	-1.378	-0.397	2.1E-02	5.0E-02
294	TRINITY_DN6930_c0_g1_i3	-1.382	-0.211	1.6E-02	4.0E-02
295	TRINITY_DN6492_c0_g2_i14	-1.386	0.571	9.6E-03	2.6E-02
296	TRINITY_DN2_c0_g2_i7	-1.397	12.472	4.1E-03	1.3E-02
297	TRINITY_DN45_c0_g1_i3	-1.411	2.164	5.3E-03	1.6E-02
298	TRINITY_DN33040_c1_g1_i4	-1.415	0.998	7.5E-03	2.2E-02
299	TRINITY_DN7386_c0_g1_i1	-1.423	4.314	3.7E-03	1.2E-02
300	TRINITY_DN258_c9_g1_i9	-1.433	0.271	9.8E-03	2.7E-02
301	TRINITY_DN129_c2_g1_i2	-1.434	5.220	3.3E-03	1.1E-02
302	TRINITY_DN1436_c0_g1_i22	-1.442	0.106	1.0E-02	2.8E-02
303	TRINITY_DN40858_c0_g1_i3	-1.465	0.255	8.7E-03	2.4E-02
304	TRINITY_DN79729_c0_g1_i3	-1.471	0.276	8.5E-03	2.4E-02
305	TRINITY_DN56812_c0_g1_i9	-1.475	0.391	8.4E-03	2.4E-02
306	TRINITY_DN0_c4_g1_i12	-1.486	3.323	2.7E-03	9.3E-03
307	TRINITY_DN45967_c3_g1_i1	-1.489	4.308	2.4E-03	8.3E-03
308	TRINITY_DN7178_c0_g1_i2	-1.490	0.240	8.7E-03	2.4E-02
309	TRINITY_DN219_c0_g1_i1	-1.499	-0.231	8.1E-03	2.3E-02
310	TRINITY_DN5997_c0_g1_i2	-1.506	1.689	3.2E-03	1.1E-02
311	TRINITY_DN16649_c0_g4_i6	-1.507	1.671	3.1E-03	1.0E-02
312	TRINITY_DN28_c0_g1_i41	-1.513	1.358	3.4E-03	1.1E-02
313	TRINITY_DN9962_c0_g1_i4	-1.517	1.578	3.3E-03	1.1E-02
314	TRINITY_DN85_c2_g2_i1	-1.523	1.118	3.5E-03	1.1E-02
315	TRINITY_DN302750_c0_g1_i2	-1.537	1.313	2.8E-03	9.6E-03
316	TRINITY_DN25790_c0_g1_i3	-1.538	0.740	4.2E-03	1.3E-02
317	TRINITY_DN384_c0_g1_i1	-1.539	0.363	5.3E-03	1.6E-02

318	TRINITY_DN464_c1_g1_i10	-1.560	0.482	4.1E-03	1.3E-02
319	TRINITY_DN213_c0_g1_i12	-1.561	-0.132	7.1E-03	2.1E-02
320	TRINITY_DN24738_c9_g1_i1	-1.569	4.343	1.3E-03	5.1E-03
321	TRINITY_DN1766_c8_g1_i1	-1.580	5.282	1.2E-03	4.5E-03
322	TRINITY_DN34794_c0_g1_i11	-1.591	-0.242	5.6E-03	1.7E-02
323	TRINITY_DN125_c4_g2_i1	-1.591	2.286	1.5E-03	5.7E-03
324	TRINITY_DN5597_c0_g1_i2	-1.592	0.864	2.6E-03	8.9E-03
325	TRINITY_DN163_c0_g1_i1	-1.592	0.266	4.3E-03	1.3E-02
326	TRINITY_DN464_c0_g2_i3	-1.600	0.112	4.9E-03	1.5E-02
327	TRINITY_DN285_c0_g1_i5	-1.602	1.229	2.2E-03	7.8E-03
328	TRINITY_DN112_c1_g2_i21	-1.604	1.368	2.0E-03	7.4E-03
329	TRINITY_DN81_c0_g1_i7	-1.607	0.500	2.8E-03	9.6E-03
330	TRINITY_DN20212_c0_g2_i3	-1.609	1.273	2.1E-03	7.6E-03
331	TRINITY_DN5567_c0_g1_i6	-1.610	-0.357	7.6E-03	2.2E-02
332	TRINITY_DN46393_c2_g1_i1	-1.624	-0.010	4.6E-03	1.4E-02
333	TRINITY_DN2_c0_g2_i1	-1.625	0.402	3.8E-03	1.2E-02
334	TRINITY_DN13140_c7_g1_i1	-1.634	1.797	1.3E-03	5.1E-03
335	TRINITY_DN108_c5_g1_i4	-1.635	1.605	1.4E-03	5.5E-03
336	TRINITY_DN222_c0_g1_i55	-1.640	1.224	1.7E-03	6.3E-03
337	TRINITY_DN5111_c0_g1_i3	-1.646	-0.171	4.3E-03	1.3E-02
338	TRINITY_DN42_c0_g1_i4	-1.648	0.058	4.3E-03	1.3E-02
339	TRINITY_DN1665_c2_g2_i9	-1.655	1.778	1.2E-03	4.8E-03
340	TRINITY_DN98_c0_g1_i14	-1.656	0.260	2.8E-03	9.6E-03
341	TRINITY_DN8_c0_g1_i13	-1.671	4.027	6.3E-04	2.7E-03
342	TRINITY_DN2568_c0_g1_i1	-1.673	-0.063	4.0E-03	1.3E-02
343	TRINITY_DN34444_c3_g1_i1	-1.674	4.321	6.2E-04	2.7E-03
344	TRINITY_DN125_c0_g1_i7	-1.674	2.124	8.3E-04	3.5E-03
345	TRINITY_DN112_c46_g1_i3	-1.675	2.644	7.6E-04	3.2E-03
346	TRINITY_DN2_c0_g1_i1	-1.678	0.724	1.8E-03	6.6E-03
347	TRINITY_DN2_c0_g1_i7	-1.679	0.422	2.2E-03	7.9E-03
348	TRINITY_DN0_c101_g2_i1	-1.690	0.396	2.3E-03	7.9E-03
349	TRINITY_DN0_c2_g2_i20	-1.690	8.448	4.7E-04	2.1E-03
350	TRINITY_DN2015_c0_g1_i1	-1.709	-0.595	4.8E-03	1.5E-02
351	TRINITY_DN2260_c1_g1_i1	-1.711	0.406	2.2E-03	7.9E-03
352	TRINITY_DN134_c1_g1_i32	-1.711	1.900	7.6E-04	3.2E-03
353	TRINITY_DN2_c94_g3_i1	-1.716	2.300	6.4E-04	2.8E-03
354	TRINITY_DN112_c0_g1_i17	-1.719	1.258	8.7E-04	3.5E-03
355	TRINITY_DN37194_c0_g1_i5	-1.731	6.684	3.5E-04	1.6E-03
356	TRINITY_DN33_c0_g1_i3	-1.734	1.372	7.6E-04	3.2E-03
357	TRINITY_DN290_c1_g1_i1	-1.736	-0.121	2.9E-03	9.7E-03
358	TRINITY_DN8_c0_g1_i19	-1.739	1.294	8.1E-04	3.4E-03
359	TRINITY_DN4093_c0_g1_i4	-1.743	0.823	1.0E-03	4.0E-03
360	TRINITY_DN23_c0_g1_i15	-1.746	1.920	5.5E-04	2.4E-03
361	TRINITY_DN1198_c0_g1_i24	-1.756	0.949	8.2E-04	3.4E-03
362	TRINITY_DN112_c1_g2_i30	-1.763	0.503	1.3E-03	5.0E-03
363	TRINITY_DN2_c1_g6_i3	-1.775	1.754	5.0E-04	2.2E-03
364	TRINITY_DN262_c1_g1_i14	-1.789	0.290	9.5E-04	3.8E-03
365	TRINITY_DN6009_c1_g1_i1	-1.805	4.664	2.0E-04	1.1E-03
366	TRINITY_DN79_c0_g1_i13	-1.806	-0.063	1.3E-03	5.1E-03
367	TRINITY_DN11_c0_g1_i1	-1.808	0.345	8.2E-04	3.4E-03
368	TRINITY_DN23_c0_g1_i14	-1.817	1.267	5.2E-04	2.3E-03
369	TRINITY_DN15564_c0_g2_i13	-1.822	0.605	8.7E-04	3.5E-03
370	TRINITY_DN81633_c0_g1_i1	-1.827	-0.018	1.3E-03	5.1E-03
371	TRINITY_DN2260_c1_g6_i2	-1.830	2.793	2.3E-04	1.2E-03
372	TRINITY_DN112_c0_g1_i2	-1.831	1.706	3.1E-04	1.5E-03

373	TRINITY_DN8337_c0_g1_i3	-1.835	0.132	9.2E-04	3.7E-03
374	TRINITY_DN119_c0_g1_i5	-1.840	-0.422	2.1E-03	7.7E-03
375	TRINITY_DN24499_c0_g2_i1	-1.852	0.592	6.7E-04	2.8E-03
376	TRINITY_DN2_c0_g3_i4	-1.852	10.431	1.2E-04	6.7E-04
377	TRINITY_DN0_c2_g2_i4	-1.854	9.316	1.2E-04	6.6E-04
378	TRINITY_DN1436_c0_g1_i2	-1.861	0.738	4.3E-04	1.9E-03
379	TRINITY_DN1202_c9_g1_i13	-1.865	0.967	4.4E-04	2.0E-03
380	TRINITY_DN2_c1_g7_i6	-1.873	12.646	1.0E-04	5.8E-04
381	TRINITY_DN709_c3_g1_i1	-1.880	3.867	1.2E-04	6.6E-04
382	TRINITY_DN2907_c0_g1_i2	-1.889	1.797	1.9E-04	9.8E-04
383	TRINITY_DN32261_c0_g3_i1	-1.900	1.624	2.0E-04	1.0E-03
384	TRINITY_DN71_c0_g1_i1	-1.917	-0.258	8.4E-04	3.5E-03
385	TRINITY_DN8347_c2_g2_i1	-1.920	0.472	4.8E-04	2.1E-03
386	TRINITY_DN2256_c11_g1_i1	-1.939	6.624	5.8E-05	3.6E-04
387	TRINITY_DN4370_c0_g1_i7	-1.940	2.741	9.0E-05	5.3E-04
388	TRINITY_DN8_c17_g1_i11	-1.949	8.329	5.1E-05	3.3E-04
389	TRINITY_DN22624_c0_g1_i11	-1.952	1.443	1.4E-04	7.8E-04
390	TRINITY_DN0_c119_g2_i1	-1.953	0.632	3.5E-04	1.6E-03
391	TRINITY_DN71_c0_g1_i3	-1.958	-0.185	5.5E-04	2.4E-03
392	TRINITY_DN86021_c0_g1_i2	-1.960	3.069	6.6E-05	4.1E-04
393	TRINITY_DN0_c524_g1_i1	-1.967	6.626	4.5E-05	3.0E-04
394	TRINITY_DN3977_c1_g1_i2	-1.975	1.788	1.1E-04	6.1E-04
395	TRINITY_DN112_c1_g2_i36	-1.976	0.252	2.9E-04	1.4E-03
396	TRINITY_DN202_c0_g1_i12	-1.978	0.272	2.9E-04	1.4E-03
397	TRINITY_DN396_c0_g1_i16	-1.979	-0.446	1.0E-03	4.0E-03
398	TRINITY_DN1778_c6_g1_i1	-1.988	2.834	5.5E-05	3.5E-04
399	TRINITY_DN2864_c0_g1_i4	-1.993	0.773	1.6E-04	8.4E-04
400	TRINITY_DN19591_c0_g2_i4	-2.002	3.361	4.2E-05	2.8E-04
401	TRINITY_DN160_c0_g1_i4	-2.022	0.572	1.5E-04	8.4E-04
402	TRINITY_DN317_c11_g1_i1	-2.029	-0.649	9.7E-04	3.9E-03
403	TRINITY_DN6170_c0_g1_i12	-2.040	-0.006	5.1E-04	2.2E-03
404	TRINITY_DN9741_c0_g1_i13	-2.050	-0.281	4.1E-04	1.9E-03
405	TRINITY_DN2260_c1_g6_i4	-2.051	0.861	1.1E-04	6.1E-04
406	TRINITY_DN4370_c0_g1_i3	-2.056	5.006	2.1E-05	1.6E-04
407	TRINITY_DN224_c0_g2_i1	-2.056	2.352	3.9E-05	2.6E-04
408	TRINITY_DN2_c1_g2_i1	-2.064	10.976	1.7E-05	1.3E-04
409	TRINITY_DN23_c0_g1_i12	-2.078	-0.293	4.1E-04	1.9E-03
410	TRINITY_DN0_c2_g1_i13	-2.084	14.562	1.4E-05	1.1E-04
411	TRINITY_DN394_c6_g1_i1	-2.090	5.671	1.5E-05	1.2E-04
412	TRINITY_DN335_c0_g1_i23	-2.097	8.869	1.3E-05	1.0E-04
413	TRINITY_DN335_c0_g1_i38	-2.113	0.822	6.8E-05	4.1E-04
414	TRINITY_DN2925_c0_g1_i4	-2.117	0.410	8.6E-05	5.1E-04
415	TRINITY_DN0_c2_g2_i12	-2.118	14.527	1.0E-05	8.4E-05
416	TRINITY_DN4359_c0_g1_i9	-2.124	0.233	9.0E-05	5.3E-04
417	TRINITY_DN396_c3_g1_i1	-2.140	0.489	7.2E-05	4.3E-04
418	TRINITY_DN3423_c2_g1_i2	-2.144	3.366	1.2E-05	1.0E-04
419	TRINITY_DN320_c9_g1_i1	-2.145	1.803	2.1E-05	1.6E-04
420	TRINITY_DN0_c3_g1_i8	-2.153	2.973	1.2E-05	1.0E-04
421	TRINITY_DN1046_c0_g1_i2	-2.158	-0.586	4.2E-04	1.9E-03
422	TRINITY_DN317_c0_g1_i13	-2.171	-0.081	1.2E-04	6.7E-04
423	TRINITY_DN0_c2_g2_i13	-2.175	14.002	5.8E-06	5.2E-05
424	TRINITY_DN3122_c12_g1_i1	-2.180	1.456	2.6E-05	1.9E-04
425	TRINITY_DN94_c0_g1_i3	-2.198	0.674	3.6E-05	2.5E-04
426	TRINITY_DN955_c1_g4_i1	-2.215	0.198	7.2E-05	4.3E-04
427	TRINITY_DN955_c1_g10_i1	-2.215	0.198	7.2E-05	4.3E-04

428	TRINITY_DN0_c2_g2_i6	-2.240	1.673	1.1E-05	9.1E-05
429	TRINITY_DN416_c0_g3_i2	-2.280	7.686	2.0E-06	2.1E-05
430	TRINITY_DN19111_c0_g4_i1	-2.305	1.929	4.9E-06	4.5E-05
431	TRINITY_DN9500_c10_g1_i1	-2.319	2.204	3.7E-06	3.5E-05
432	TRINITY_DN18788_c0_g3_i4	-2.321	0.053	5.2E-05	3.4E-04
433	TRINITY_DN0_c2_g2_i9	-2.324	13.493	1.2E-06	1.3E-05
434	TRINITY_DN1015_c1_g2_i2	-2.325	0.028	3.8E-05	2.6E-04
435	TRINITY_DN3423_c0_g1_i1	-2.328	0.435	1.8E-05	1.4E-04
436	TRINITY_DN1379_c0_g1_i14	-2.338	0.964	6.3E-06	5.5E-05
437	TRINITY_DN93_c0_g1_i30	-2.338	2.066	2.8E-06	2.8E-05
438	TRINITY_DN208810_c5_g1_i1	-2.340	0.762	9.8E-06	8.2E-05
439	TRINITY_DN28_c0_g1_i20	-2.355	-0.175	3.7E-05	2.5E-04
440	TRINITY_DN878_c0_g1_i6	-2.381	2.904	1.2E-06	1.3E-05
441	TRINITY_DN1293_c29_g1_i1	-2.397	0.715	7.1E-06	6.2E-05
442	TRINITY_DN6170_c0_g1_i11	-2.434	2.277	1.1E-06	1.2E-05
443	TRINITY_DN20209_c8_g1_i1	-2.440	2.682	7.7E-07	8.9E-06
444	TRINITY_DN1109_c0_g1_i2	-2.443	0.477	5.6E-06	5.1E-05
445	TRINITY_DN696_c1_g1_i1	-2.500	2.149	6.1E-07	7.3E-06
446	TRINITY_DN0_c119_g1_i2	-2.603	7.428	5.2E-08	8.4E-07
447	TRINITY_DN212658_c0_g1_i1	-2.619	-0.049	4.9E-06	4.5E-05
448	TRINITY_DN8_c0_g1_i1	-2.621	1.485	2.8E-07	3.8E-06
449	TRINITY_DN1441_c0_g1_i3	-2.627	5.499	4.3E-08	7.2E-07
450	TRINITY_DN298138_c0_g1_i1	-2.708	0.406	5.7E-07	6.9E-06
451	TRINITY_DN7444_c1_g2_i1	-2.729	2.083	4.7E-08	7.6E-07
452	TRINITY_DN5959_c0_g1_i2	-2.734	2.841	2.3E-08	4.1E-07
453	TRINITY_DN22156_c6_g1_i1	-2.739	1.559	8.3E-08	1.2E-06
454	TRINITY_DN5597_c0_g1_i5	-2.754	0.947	1.3E-07	1.9E-06
455	TRINITY_DN56774_c0_g1_i1	-2.768	0.593	2.6E-07	3.6E-06
456	TRINITY_DN0_c2_g1_i18	-2.770	2.213	3.1E-08	5.4E-07
457	TRINITY_DN1441_c0_g1_i6	-2.776	8.122	6.2E-09	1.2E-07
458	TRINITY_DN6472_c4_g2_i1	-2.785	0.862	1.5E-07	2.1E-06
459	TRINITY_DN353_c0_g1_i3	-2.786	1.795	2.8E-08	4.9E-07
460	TRINITY_DN224_c0_g1_i39	-2.953	0.396	5.7E-08	9.0E-07
461	TRINITY_DN23_c6_g1_i8	-2.957	0.332	6.7E-08	1.0E-06
462	TRINITY_DN290_c3_g2_i1	-2.969	0.468	6.9E-08	1.0E-06
463	TRINITY_DN3423_c0_g1_i2	-3.001	4.491	4.5E-10	9.9E-09
464	TRINITY_DN228_c0_g1_i2	-3.033	0.479	3.5E-08	6.0E-07
465	TRINITY_DN853_c0_g1_i12	-3.037	5.859	2.2E-10	5.0E-09
466	TRINITY_DN335_c0_g1_i25	-3.057	0.680	1.4E-08	2.5E-07
467	TRINITY_DN17218_c5_g1_i1	-3.079	1.190	2.6E-09	5.3E-08
468	TRINITY_DN14311_c0_g7_i2	-3.099	1.464	1.9E-09	4.0E-08
469	TRINITY_DN0_c3_g1_i1	-3.180	4.823	3.8E-11	9.5E-10
470	TRINITY_DN17650_c0_g1_i3	-3.250	0.226	5.4E-09	1.0E-07
471	TRINITY_DN617_c0_g1_i9	-3.278	1.528	1.8E-10	4.2E-09
472	TRINITY_DN93_c0_g1_i37	-3.301	1.241	5.7E-10	1.2E-08
473	TRINITY_DN1202_c9_g1_i5	-3.331	6.645	3.3E-12	9.2E-11
474	TRINITY_DN131_c0_g1_i16	-3.383	1.814	2.7E-11	7.1E-10
475	TRINITY_DN0_c70_g2_i1	-3.410	2.522	9.0E-12	2.4E-10
476	TRINITY_DN335_c17_g1_i1	-3.436	1.229	5.0E-11	1.2E-09
477	TRINITY_DN1202_c9_g1_i6	-3.446	5.350	7.2E-13	2.2E-11
478	TRINITY_DN7196_c0_g2_i6	-3.449	3.293	1.7E-12	5.0E-11
479	TRINITY_DN853_c1_g1_i1	-3.452	1.783	1.2E-11	3.2E-10
480	TRINITY_DN714_c0_g1_i24	-3.480	0.711	1.5E-10	3.5E-09
481	TRINITY_DN3423_c0_g1_i3	-3.583	4.558	1.3E-13	4.1E-12
482	TRINITY_DN0_c2_g1_i5	-3.620	8.988	3.9E-14	1.3E-12

483	TRINITY_DN64_c0_g2_i2	-3.628	5.452	4.9E-14	1.6E-12
484	TRINITY_DN64_c0_g2_i21	-3.663	6.329	2.3E-14	8.1E-13
485	TRINITY_DN50096_c4_g1_i1	-3.682	5.454	2.1E-14	7.6E-13
486	TRINITY_DN112_c1_g2_i28	-3.703	1.477	9.5E-13	2.9E-11
487	TRINITY_DN2429_c2_g1_i2	-3.741	3.012	3.7E-14	1.3E-12
488	TRINITY_DN59648_c0_g1_i1	-3.796	5.655	3.0E-15	1.2E-13
489	TRINITY_DN2_c54_g1_i1	-3.887	3.489	2.4E-15	9.6E-14
490	TRINITY_DN93_c0_g1_i20	-3.910	3.071	2.2E-15	9.0E-14
491	TRINITY_DN853_c0_g1_i3	-4.102	1.972	1.4E-15	6.3E-14
492	TRINITY_DN853_c0_g1_i2	-4.171	4.561	1.2E-17	6.6E-16
493	TRINITY_DN21625_c1_g1_i2	-4.188	5.566	6.1E-18	3.5E-16
494	TRINITY_DN417_c1_g1_i1	-4.215	2.820	3.5E-17	1.8E-15
495	TRINITY_DN24221_c0_g2_i4	-4.218	1.510	4.4E-15	1.7E-13
496	TRINITY_DN1441_c0_g1_i5	-4.283	4.053	2.5E-18	1.6E-16
497	TRINITY_DN64_c0_g2_i19	-4.427	6.386	7.1E-20	5.1E-18
498	TRINITY_DN459_c4_g2_i15	-4.464	1.142	1.8E-16	8.7E-15
499	TRINITY_DN506_c1_g1_i1	-4.505	1.223	8.5E-17	4.3E-15
500	TRINITY_DN853_c0_g1_i11	-4.534	2.100	2.9E-18	1.8E-16
501	TRINITY_DN112_c1_g2_i6	-4.549	5.458	1.3E-20	1.0E-18
502	TRINITY_DN1433_c6_g1_i1	-4.826	3.895	4.2E-22	3.4E-20
503	TRINITY_DN2442_c0_g1_i2	-4.930	5.677	1.5E-23	1.4E-21
504	TRINITY_DN0_c2_g1_i8	-4.948	11.462	5.0E-24	6.1E-22
505	TRINITY_DN1441_c0_g1_i4	-4.955	4.880	1.4E-23	1.4E-21
506	TRINITY_DN1441_c0_g1_i7	-5.241	2.653	1.5E-23	1.4E-21
507	TRINITY_DN459_c4_g2_i2	-5.303	3.754	1.9E-25	2.6E-23
508	TRINITY_DN2429_c2_g1_i1	-5.384	4.241	1.6E-26	2.4E-24
509	TRINITY_DN853_c0_g1_i16	-5.387	6.016	2.7E-27	4.7E-25
510	TRINITY_DN11703_c0_g2_i2	-6.141	6.171	2.0E-33	4.1E-31
511	TRINITY_DN1202_c9_g1_i4	-6.339	8.381	1.3E-35	3.9E-33
512	TRINITY_DN1293_c1_g2_i3	-6.383	6.234	2.2E-35	5.4E-33
513	TRINITY_DN2822_c0_g2_i2	-7.428	5.638	2.4E-43	1.5E-40
514	TRINITY_DN0_c3_g1_i2	-7.899	7.032	1.9E-48	2.2E-45

Table S2. **Transcripts of differentially expressed genes (DEGs) in RPE during the late stage of the newt retina regeneration**.

NN		logFC	logCPM	P-Value	FDR
1	TRINITY_DN955_c1_g2_i3	22.735	14.025	7.1E-47	8.7E-44
2	TRINITY_DN31589_c1_g1_i1	15.765	7.058	6.6E-26	1.6E-23
3	TRINITY_DN311_c0_g1_i3	11.371	2.708	1.2E-13	6.5E-12
4	TRINITY_DN29373_c0_g1_i15	11.144	2.486	5.5E-13	2.3E-11
5	TRINITY_DN955_c1_g2_i19	10.959	6.160	1.4E-22	2.9E-20
6	TRINITY_DN37667_c0_g1_i9	10.747	2.106	7.4E-12	2.5E-10
7	TRINITY_DN955_c1_g2_i9	10.598	9.165	1.4E-26	4.2E-24
8	TRINITY_DN33040_c1_g1_i2	10.321	1.694	1.1E-10	3.0E-09
9	TRINITY_DN10266_c0_g1_i8	10.075	1.453	5.3E-10	1.2E-08
10	TRINITY_DN137_c0_g1_i1	9.956	1.362	1.1E-09	2.3E-08
11	TRINITY_DN873_c0_g1_i18	9.866	1.268	1.9E-09	3.6E-08
12	TRINITY_DN2566_c0_g1_i10	9.831	1.226	2.4E-09	4.4E-08
13	TRINITY_DN149_c0_g1_i4	9.756	1.157	3.8E-09	6.7E-08
14	TRINITY_DN11067_c0_g3_i6	9.688	1.097	5.7E-09	9.4E-08
15	TRINITY_DN31_c0_g1_i7	9.661	1.071	6.8E-09	1.1E-07
16	TRINITY_DN2846_c0_g1_i7	9.641	1.058	7.8E-09	1.2E-07
17	TRINITY_DN317_c2_g1_i3	9.623	1.032	8.6E-09	1.3E-07

18	TRINITY_DN224_c3_g1_i1	9.462	0.891	2.2E-08	2.8E-07
19	TRINITY_DN311_c0_g1_i4	9.365	0.779	3.9E-08	4.8E-07
20	TRINITY_DN43340_c2_g1_i3	9.363	4.575	8.3E-18	7.8E-16
21	TRINITY_DN124361_c0_g1_i10	9.287	0.712	6.2E-08	7.2E-07
22	TRINITY_DN2846_c0_g1_i5	9.270	0.701	6.9E-08	7.8E-07
23	TRINITY_DN1536_c0_g1_i7	9.266	0.694	6.9E-08	7.8E-07
24	TRINITY_DN32007_c0_g1_i5	9.262	0.690	7.3E-08	8.1E-07
25	TRINITY_DN746_c0_g1_i2	9.178	0.637	1.2E-07	1.2E-06
26	TRINITY_DN6412_c0_g2_i8	9.168	0.598	1.2E-07	1.2E-06
27	TRINITY_DN119_c3_g1_i4	9.147	0.587	1.4E-07	1.4E-06
28	TRINITY_DN6763_c2_g1_i2	9.138	0.576	1.4E-07	1.4E-06
29	TRINITY_DN99_c0_g1_i2	9.031	0.486	2.7E-07	2.3E-06
30	TRINITY_DN192_c0_g1_i3	9.001	0.466	3.3E-07	2.8E-06
31	TRINITY_DN341_c0_g1_i4	8.972	0.426	3.8E-07	3.2E-06
32	TRINITY_DN112_c1_g2_i20	8.907	0.375	5.4E-07	4.4E-06
33	TRINITY_DN196_c0_g1_i3	8.886	0.365	6.2E-07	5.0E-06
34	TRINITY_DN197_c0_g1_i16	8.820	0.281	8.9E-07	6.9E-06
35	TRINITY_DN221_c0_g1_i14	8.805	0.290	9.6E-07	7.3E-06
36	TRINITY_DN552_c0_g1_i1	8.795	0.266	9.6E-07	7.3E-06
37	TRINITY_DN1425_c0_g1_i2	8.779	0.250	1.1E-06	8.3E-06
38	TRINITY_DN727_c0_g1_i2	8.735	0.223	1.4E-06	1.0E-05
39	TRINITY_DN246_c0_g1_i7	8.677	0.155	1.9E-06	1.3E-05
40	TRINITY_DN394_c0_g1_i2	8.669	0.169	1.9E-06	1.3E-05
41	TRINITY_DN7379_c0_g1_i9	8.667	0.147	1.9E-06	1.3E-05
42	TRINITY_DN222_c0_g1_i30	8.638	0.134	2.3E-06	1.5E-05
43	TRINITY_DN18777_c0_g1_i1	8.636	0.125	2.3E-06	1.5E-05
44	TRINITY_DN4697_c0_g1_i18	8.630	0.126	2.5E-06	1.6E-05
45	TRINITY_DN122953_c0_g1_i1	8.630	0.122	2.5E-06	1.6E-05
46	TRINITY_DN1070_c0_g1_i5	8.614	0.095	2.7E-06	1.7E-05
47	TRINITY_DN0_c5_g2_i2	8.590	0.080	2.9E-06	1.8E-05
48	TRINITY_DN59_c0_g1_i3	8.565	0.062	3.5E-06	2.1E-05
49	TRINITY_DN6863_c0_g1_i1	8.546	0.044	3.8E-06	2.3E-05
50	TRINITY_DN972_c0_g1_i3	8.491	-0.018	4.9E-06	2.8E-05
51	TRINITY_DN52_c0_g1_i10	8.482	-0.015	5.4E-06	3.1E-05
52	TRINITY_DN241_c1_g1_i10	8.467	-0.026	5.9E-06	3.3E-05
53	TRINITY_DN705_c0_g1_i11	8.437	-0.063	6.5E-06	3.6E-05
54	TRINITY_DN4980_c0_g1_i1	8.416	-0.086	7.1E-06	3.9E-05
55	TRINITY_DN5827_c0_g1_i18	8.396	-0.097	8.5E-06	4.5E-05
56	TRINITY_DN25407_c0_g1_i6	8.394	-0.093	8.5E-06	4.5E-05
57	TRINITY_DN279_c0_g1_i2	8.388	-0.090	8.5E-06	4.5E-05
58	TRINITY_DN8721_c0_g1_i9	8.375	-0.117	9.4E-06	4.8E-05
59	TRINITY_DN115_c0_g1_i1	8.336	-0.139	1.1E-05	5.6E-05
60	TRINITY_DN18675_c0_g1_i7	8.328	-0.159	1.1E-05	5.6E-05
61	TRINITY_DN3920_c0_g1_i2	8.327	-0.152	1.1E-05	5.6E-05
62	TRINITY_DN5397_c0_g1_i9	8.316	-0.168	1.2E-05	6.1E-05
63	TRINITY_DN13924_c0_g1_i7	8.289	-0.181	1.5E-05	7.0E-05
64	TRINITY_DN8469_c0_g1_i3	8.286	-0.192	1.5E-05	7.0E-05
65	TRINITY_DN3138_c5_g2_i1	8.282	7.198	6.8E-20	9.5E-18
66	TRINITY_DN351_c0_g1_i4	8.266	-0.215	1.5E-05	7.0E-05
67	TRINITY_DN2616_c0_g1_i1	8.256	-0.217	1.7E-05	7.6E-05
68	TRINITY_DN394_c0_g1_i7	8.238	-0.245	1.9E-05	8.2E-05
69	TRINITY_DN410_c0_g2_i1	8.234	-0.231	1.9E-05	8.2E-05
70	TRINITY_DN2846_c0_g4_i1	8.226	-0.250	1.9E-05	8.2E-05
71	TRINITY_DN1642_c0_g1_i2	8.220	-0.257	2.1E-05	8.9E-05
72	TRINITY_DN572_c1_g1_i1	8.216	-0.247	2.1E-05	8.9E-05

73	TRINITY_DN158_c0_g1_i3	8.196	-0.267	2.3E-05	9.8E-05
74	TRINITY_DN119_c0_g1_i13	8.178	-0.293	2.5E-05	1.1E-04
75	TRINITY_DN4172_c0_g2_i1	8.147	-0.315	2.8E-05	1.2E-04
76	TRINITY_DN335_c1_g1_i4	8.125	-0.340	3.1E-05	1.3E-04
77	TRINITY_DN103_c0_g1_i10	8.115	-0.347	3.5E-05	1.4E-04
78	TRINITY_DN268_c0_g1_i2	8.112	-0.345	3.5E-05	1.4E-04
79	TRINITY_DN9057_c1_g1_i4	8.077	-0.377	3.9E-05	1.6E-04
80	TRINITY_DN253871_c0_g1_i1	8.042	-0.415	4.8E-05	1.9E-04
81	TRINITY_DN2341_c0_g2_i15	8.032	-0.422	4.8E-05	1.9E-04
82	TRINITY_DN2_c0_g5_i1	8.032	8.345	7.0E-20	9.5E-18
83	TRINITY_DN341_c0_g1_i7	8.003	-0.442	6.0E-05	2.3E-04
84	TRINITY_DN9235_c0_g1_i2	7.925	3.161	1.4E-13	6.8E-12
85	TRINITY_DN4172_c0_g2_i12	7.911	-0.528	8.5E-05	3.1E-04
86	TRINITY_DN443_c0_g1_i13	7.638	2.882	9.3E-13	3.7E-11
87	TRINITY_DN29373_c0_g1_i10	7.585	2.831	1.3E-12	5.0E-11
88	TRINITY_DN443_c0_g1_i6	6.247	3.010	2.4E-11	6.9E-10
89	TRINITY_DN4607_c0_g1_i1	6.141	1.433	1.3E-08	1.8E-07
90	TRINITY_DN181_c0_g3_i8	6.112	12.894	1.3E-14	8.8E-13
91	TRINITY_DN1271_c0_g1_i5	5.882	1.201	6.3E-08	7.2E-07
92	TRINITY_DN2561_c0_g1_i3	5.703	1.030	1.8E-07	1.6E-06
93	TRINITY_DN34364_c0_g1_i2	5.487	0.832	6.4E-07	5.1E-06
94	TRINITY_DN516_c0_g2_i4	5.381	2.183	6.1E-09	9.9E-08
95	TRINITY_DN10266_c0_g1_i10	5.342	2.835	6.5E-10	1.4E-08
96	TRINITY_DN36640_c0_g1_i2	5.292	1.545	6.1E-08	7.2E-07
97	TRINITY_DN7337_c0_g1_i1	5.255	0.615	2.3E-06	1.5E-05
98	TRINITY_DN17_c0_g1_i2	5.251	1.509	8.0E-08	8.8E-07
99	TRINITY_DN123_c0_g3_i1	5.239	0.607	2.6E-06	1.6E-05
100	TRINITY_DN1393_c0_g1_i1	5.188	1.450	1.2E-07	1.2E-06
101	TRINITY_DN757_c0_g1_i2	5.094	0.486	5.8E-06	3.3E-05
102	TRINITY_DN181_c0_g3_i6	5.036	6.552	2.8E-11	8.0E-10
103	TRINITY_DN394_c0_g1_i11	5.014	0.403	8.6E-06	4.5E-05
104	TRINITY_DN30_c0_g1_i23	4.988	0.385	9.8E-06	5.0E-05
105	TRINITY_DN7379_c0_g1_i14	4.964	0.357	1.1E-05	5.6E-05
106	TRINITY_DN9_c0_g1_i3	4.943	0.328	1.2E-05	6.0E-05
107	TRINITY_DN2_c0_g2_i1	4.924	6.051	7.2E-11	1.9E-09
108	TRINITY_DN5308_c0_g1_i6	4.910	0.298	1.5E-05	7.0E-05
109	TRINITY_DN1719_c0_g1_i1	4.894	0.283	1.6E-05	7.3E-05
110	TRINITY_DN3885_c0_g1_i4	4.879	0.273	1.7E-05	7.8E-05
111	TRINITY_DN18749_c0_g1_i2	4.753	0.159	3.4E-05	1.4E-04
112	TRINITY_DN5782_c0_g1_i3	4.724	0.150	4.0E-05	1.6E-04
113	TRINITY_DN11263_c0_g1_i10	4.713	5.371	3.4E-10	8.1E-09
114	TRINITY_DN2_c14_g1_i2	4.699	0.119	4.6E-05	1.8E-04
115	TRINITY_DN140_c0_g2_i1	4.698	0.112	4.3E-05	1.7E-04
116	TRINITY_DN11_c0_g1_i17	4.655	1.500	5.0E-07	4.1E-06
117	TRINITY_DN3038_c0_g1_i1	4.629	1.454	5.7E-07	4.6E-06
118	TRINITY_DN2260_c1_g6_i3	4.582	3.552	4.3E-09	7.5E-08
119	TRINITY_DN2341_c0_g2_i4	4.581	0.004	7.5E-05	2.8E-04
120	TRINITY_DN209_c0_g1_i1	4.572	0.884	4.0E-06	2.4E-05
121	TRINITY_DN11_c0_g1_i8	4.510	1.742	2.2E-07	1.9E-06
122	TRINITY_DN308539_c0_g1_i1	4.497	-0.071	1.2E-04	4.0E-04
123	TRINITY_DN2_c1_g1_i2	4.472	0.778	7.0E-06	3.9E-05
124	TRINITY_DN149_c0_g1_i13	4.395	-0.163	2.0E-04	6.4E-04
125	TRINITY_DN676_c0_g2_i1	4.359	0.688	1.3E-05	6.2E-05
126	TRINITY_DN7205_c0_g1_i2	4.350	-0.192	2.4E-04	7.5E-04
127	TRINITY_DN15_c0_g2_i6	4.344	1.591	5.9E-07	4.8E-06

128	TRINITY_DN939_c0_g1_i5	4.337	3.506	1.5E-08	1.9E-07
129	TRINITY_DN72_c0_g1_i1	4.310	-0.234	2.9E-04	8.9E-04
130	TRINITY_DN335_c1_g1_i13	4.298	1.166	4.0E-06	2.3E-05
131	TRINITY_DN379_c0_g1_i1	4.285	1.526	8.1E-07	6.4E-06
132	TRINITY_DN35997_c0_g3_i2	4.272	-0.266	3.5E-04	1.0E-03
133	TRINITY_DN108_c5_g1_i4	4.235	6.622	4.4E-09	7.5E-08
134	TRINITY_DN210_c0_g1_i1	4.233	1.482	1.1E-06	8.2E-06
135	TRINITY_DN394_c0_g1_i14	4.222	1.097	6.0E-06	3.4E-05
136	TRINITY_DN2846_c0_g1_i4	4.217	1.482	1.2E-06	9.1E-06
137	TRINITY_DN1202_c9_g1_i11	4.185	0.516	3.2E-05	1.3E-04
138	TRINITY_DN4172_c0_g2_i10	4.176	0.520	3.4E-05	1.4E-04
139	TRINITY_DN80024_c0_g1_i2	4.171	-0.359	5.2E-04	1.4E-03
140	TRINITY_DN11204_c0_g1_i1	4.146	1.007	4.2E-06	2.5E-05
141	TRINITY_DN41697_c0_g1_i8	4.136	-0.387	6.3E-04	1.7E-03
142	TRINITY_DN11654_c0_g1_i15	4.122	0.462	4.4E-05	1.7E-04
143	TRINITY_DN181_c0_g3_i1	4.102	9.008	8.2E-09	1.2E-07
144	TRINITY_DN955_c1_g4_i1	4.101	5.293	1.4E-08	1.9E-07
145	TRINITY_DN955_c1_g10_i1	4.101	5.293	1.4E-08	1.9E-07
146	TRINITY_DN5490_c0_g1_i4	4.096	-0.418	7.8E-04	2.0E-03
147	TRINITY_DN2260_c1_g6_i2	4.081	7.769	1.0E-08	1.4E-07
148	TRINITY_DN179_c0_g1_i15	4.073	2.952	1.1E-07	1.1E-06
149	TRINITY_DN1291_c0_g2_i6	4.034	0.386	6.8E-05	2.5E-04
150	TRINITY_DN405_c0_g1_i1	4.028	0.395	7.3E-05	2.7E-04
151	TRINITY_DN459_c4_g2_i3	4.014	1.277	3.9E-06	2.3E-05
152	TRINITY_DN28603_c1_g1_i1	4.010	0.884	8.8E-06	4.6E-05
153	TRINITY_DN12229_c1_g1_i5	3.996	1.570	2.5E-06	1.6E-05
154	TRINITY_DN34442_c0_g1_i11	3.995	0.352	8.3E-05	3.0E-04
155	TRINITY_DN291_c0_g1_i5	3.978	0.348	9.5E-05	3.4E-04
156	TRINITY_DN1273_c0_g1_i3	3.947	0.843	1.2E-05	6.1E-05
157	TRINITY_DN7205_c0_g1_i18	3.934	0.307	1.2E-04	4.0E-04
158	TRINITY_DN13170_c0_g1_i4	3.932	0.310	1.2E-04	4.0E-04
159	TRINITY_DN179_c0_g1_i21	3.897	4.416	7.1E-08	8.0E-07
160	TRINITY_DN50515_c0_g1_i1	3.889	0.255	1.4E-04	4.8E-04
161	TRINITY_DN3178_c0_g1_i1	3.881	0.789	1.8E-05	8.0E-05
162	TRINITY_DN6412_c0_g2_i3	3.877	0.257	1.5E-04	5.1E-04
163	TRINITY_DN102_c0_g1_i1	3.872	3.301	2.0E-07	1.8E-06
164	TRINITY_DN82_c0_g1_i4	3.868	5.665	5.2E-08	6.2E-07
165	TRINITY_DN2_c94_g3_i1	3.841	6.985	4.9E-08	6.0E-07
166	TRINITY_DN6763_c2_g6_i2	3.822	1.858	2.0E-06	1.4E-05
167	TRINITY_DN90912_c0_g1_i1	3.798	0.170	2.2E-04	7.0E-04
168	TRINITY_DN26301_c9_g1_i1	3.779	5.742	8.5E-08	9.2E-07
169	TRINITY_DN8131_c0_g2_i1	3.758	0.144	2.8E-04	8.6E-04
170	TRINITY_DN8537_c0_g1_i2	3.741	1.027	1.7E-05	7.8E-05
171	TRINITY_DN4702_c0_g1_i1	3.733	3.905	2.3E-07	2.0E-06
172	TRINITY_DN83_c0_g1_i1	3.719	0.117	3.2E-04	9.7E-04
173	TRINITY_DN9754_c0_g3_i3	3.714	1.762	3.7E-06	2.3E-05
174	TRINITY_DN2556_c0_g1_i10	3.710	1.015	2.1E-05	9.0E-05
175	TRINITY_DN28_c0_g3_i1	3.699	2.095	2.9E-06	1.8E-05
176	TRINITY_DN292_c0_g2_i2	3.696	0.604	4.7E-05	1.9E-04
177	TRINITY_DN254135_c0_g1_i1	3.679	0.060	3.7E-04	1.1E-03
178	TRINITY_DN222_c0_g1_i64	3.668	1.249	1.5E-05	7.0E-05
179	TRINITY_DN1708_c0_g2_i2	3.656	0.564	5.6E-05	2.1E-04
180	TRINITY_DN208_c0_g1_i11	3.645	0.558	6.3E-05	2.4E-04
181	TRINITY_DN1441_c0_g1_i12	3.639	0.559	6.3E-05	2.4E-04
182	TRINITY_DN40630_c0_g1_i3	3.633	5.826	2.1E-07	1.9E-06

183	TRINITY_DN1779_c0_g1_i15	3.629	1.242	1.9E-05	8.4E-05
184	TRINITY_DN317_c0_g1_i8	3.617	1.860	5.4E-06	3.1E-05
185	TRINITY_DN652_c0_g1_i19	3.605	0.009	5.5E-04	1.5E-03
186	TRINITY_DN129_c2_g1_i2	3.600	9.545	1.9E-07	1.7E-06
187	TRINITY_DN260_c0_g2_i1	3.584	1.824	6.5E-06	3.6E-05
188	TRINITY_DN31589_c1_g1_i2	3.568	1.428	1.8E-05	8.2E-05
189	TRINITY_DN80_c1_g1_i33	3.568	4.169	5.0E-07	4.1E-06
190	TRINITY_DN42741_c0_g1_i2	3.543	1.611	1.0E-05	5.1E-05
191	TRINITY_DN38178_c0_g1_i16	3.481	0.783	6.7E-05	2.5E-04
192	TRINITY_DN203_c0_g1_i1	3.477	0.426	1.5E-04	4.9E-04
193	TRINITY_DN955_c1_g2_i20	3.447	0.754	8.2E-05	3.0E-04
194	TRINITY_DN341_c0_g1_i2	3.427	-0.143	1.3E-03	3.0E-03
195	TRINITY_DN617_c0_g1_i7	3.404	0.714	1.0E-04	3.6E-04
196	TRINITY_DN226077_c0_g1_i1	3.401	1.650	1.8E-05	8.0E-05
197	TRINITY_DN149_c0_g1_i5	3.396	0.702	1.1E-04	3.7E-04
198	TRINITY_DN1107_c0_g3_i3	3.393	0.328	2.2E-04	7.0E-04
199	TRINITY_DN270767_c0_g1_i1	3.390	7.267	7.5E-07	5.9E-06
200	TRINITY_DN8544_c0_g1_i1	3.383	0.715	1.2E-04	4.1E-04
201	TRINITY_DN7688_c0_g3_i1	3.367	1.925	1.2E-05	5.8E-05
202	TRINITY_DN147_c0_g1_i15	3.359	0.304	2.5E-04	7.8E-04
203	TRINITY_DN131_c0_g1_i26	3.358	4.157	1.6E-06	1.2E-05
204	TRINITY_DN1479_c0_g2_i3	3.333	3.288	3.2E-06	2.0E-05
205	TRINITY_DN123222_c0_g1_i1	3.330	0.646	1.5E-04	4.9E-04
206	TRINITY_DN528_c0_g1_i2	3.324	0.274	3.1E-04	9.4E-04
207	TRINITY_DN2859_c0_g1_i5	3.319	-0.252	2.0E-03	4.4E-03
208	TRINITY_DN1810_c0_g1_i1	3.319	-0.252	2.0E-03	4.4E-03
209	TRINITY_DN2859_c0_g1_i6	3.319	-0.252	2.0E-03	4.4E-03
210	TRINITY_DN129_c0_g5_i1	3.316	0.949	1.0E-04	3.5E-04
211	TRINITY_DN11263_c0_g1_i15	3.308	4.736	1.7E-06	1.2E-05
212	TRINITY_DN76_c0_g1_i7	3.302	-0.261	2.1E-03	4.7E-03
213	TRINITY_DN1031_c0_g1_i1	3.300	-0.269	2.1E-03	4.7E-03
214	TRINITY_DN73384_c0_g1_i1	3.298	-0.264	2.1E-03	4.7E-03
215	TRINITY_DN11701_c0_g2_i1	3.288	0.244	3.5E-04	1.0E-03
216	TRINITY_DN290_c0_g1_i15	3.269	2.660	7.2E-06	3.9E-05
217	TRINITY_DN4726_c0_g1_i2	3.269	0.231	4.1E-04	1.2E-03
218	TRINITY_DN92_c0_g1_i8	3.268	0.895	1.2E-04	4.2E-04
219	TRINITY_DN290_c0_g1_i19	3.263	2.397	9.4E-06	4.8E-05
220	TRINITY_DN4607_c0_g2_i1	3.243	-0.310	2.6E-03	5.5E-03
221	TRINITY_DN19569_c0_g1_i2	3.237	4.707	2.7E-06	1.7E-05
222	TRINITY_DN19483_c0_g1_i4	3.235	0.199	4.7E-04	1.3E-03
223	TRINITY_DN851_c0_g1_i14	3.224	0.188	5.0E-04	1.4E-03
224	TRINITY_DN2965_c0_g1_i2	3.215	0.191	5.0E-04	1.4E-03
225	TRINITY_DN179_c0_g1_i7	3.206	7.350	2.2E-06	1.5E-05
226	TRINITY_DN137_c0_g1_i2	3.202	2.436	1.3E-05	6.4E-05
227	TRINITY_DN179_c0_g1_i11	3.198	6.929	2.4E-06	1.6E-05
228	TRINITY_DN18749_c0_g2_i3	3.184	0.143	5.8E-04	1.6E-03
229	TRINITY_DN59306_c0_g1_i2	3.174	0.520	3.2E-04	9.7E-04
230	TRINITY_DN64_c0_g2_i1	3.157	2.707	1.4E-05	6.6E-05
231	TRINITY_DN27_c0_g1_i1	3.153	0.123	6.7E-04	1.8E-03
232	TRINITY_DN13082_c0_g3_i4	3.124	1.708	4.5E-05	1.8E-04
233	TRINITY_DN77822_c0_g1_i2	3.120	-0.421	2.1E-03	4.7E-03
234	TRINITY_DN18248_c0_g2_i5	3.119	0.484	4.3E-04	1.2E-03
235	TRINITY_DN112_c1_g2_i22	3.114	1.562	5.1E-05	2.0E-04
236	TRINITY_DN19569_c0_g1_i7	3.112	5.354	4.7E-06	2.7E-05
237	TRINITY_DN131_c0_g1_i7	3.106	3.701	8.4E-06	4.5E-05

238	TRINITY_DN60_c0_g1_i2	3.100	-0.435	2.4E-03	5.1E-03
239	TRINITY_DN506_c1_g1_i6	3.095	2.838	1.6E-05	7.4E-05
240	TRINITY_DN501_c0_g1_i1	3.093	0.462	4.8E-04	1.4E-03
241	TRINITY_DN311_c0_g1_i6	3.085	3.984	8.0E-06	4.3E-05
242	TRINITY_DN4172_c0_g2_i14	3.084	0.736	3.2E-04	9.8E-04
243	TRINITY_DN4615_c0_g1_i1	3.077	-0.453	2.4E-03	5.1E-03
244	TRINITY_DN2367_c0_g1_i2	3.076	0.440	5.1E-04	1.4E-03
245	TRINITY_DN2859_c0_g1_i1	3.047	-0.481	2.9E-03	6.1E-03
246	TRINITY_DN2859_c0_g1_i15	3.047	-0.481	2.9E-03	6.1E-03
247	TRINITY_DN7288_c0_g1_i16	3.029	0.020	1.2E-03	2.9E-03
248	TRINITY_DN2412_c0_g1_i3	3.005	0.367	7.0E-04	1.8E-03
249	TRINITY_DN300430_c0_g1_i1	3.004	-0.002	1.3E-03	3.2E-03
250	TRINITY_DN594_c0_g1_i2	2.990	0.889	2.3E-04	7.1E-04
251	TRINITY_DN6930_c0_g1_i16	2.972	0.342	8.4E-04	2.1E-03
252	TRINITY_DN7231_c0_g1_i1	2.953	1.408	1.2E-04	4.1E-04
253	TRINITY_DN639_c0_g1_i3	2.952	1.554	1.1E-04	3.9E-04
254	TRINITY_DN1812_c0_g1_i5	2.949	1.409	1.2E-04	4.2E-04
255	TRINITY_DN1015_c0_g1_i7	2.939	1.068	2.5E-04	7.7E-04
256	TRINITY_DN82_c0_g1_i2	2.934	2.565	3.9E-05	1.6E-04
257	TRINITY_DN25176_c0_g2_i1	2.933	-0.069	1.8E-03	4.2E-03
258	TRINITY_DN19677_c0_g1_i14	2.933	-0.069	1.8E-03	4.2E-03
259	TRINITY_DN82_c0_g1_i7	2.930	1.999	7.8E-05	2.9E-04
260	TRINITY_DN138613_c1_g1_i1	2.927	0.298	1.0E-03	2.5E-03
261	TRINITY_DN70900_c0_g3_i1	2.924	0.294	1.0E-03	2.5E-03
262	TRINITY_DN1245_c2_g1_i4	2.920	1.532	1.3E-04	4.5E-04
263	TRINITY_DN40630_c0_g1_i2	2.915	4.745	1.6E-05	7.3E-05
264	TRINITY_DN300822_c0_g1_i1	2.899	2.597	4.8E-05	1.9E-04
265	TRINITY_DN6573_c0_g1_i1	2.861	-0.127	2.5E-03	5.4E-03
266	TRINITY_DN129_c0_g4_i1	2.841	0.962	3.9E-04	1.1E-03
267	TRINITY_DN2215_c1_g2_i12	2.838	0.763	5.0E-04	1.4E-03
268	TRINITY_DN418_c0_g1_i3	2.830	0.506	1.0E-03	2.6E-03
269	TRINITY_DN535_c0_g1_i3	2.817	1.898	1.4E-04	4.8E-04
270	TRINITY_DN13295_c0_g1_i26	2.814	0.481	1.1E-03	2.7E-03
271	TRINITY_DN35520_c0_g1_i1	2.811	-0.169	3.0E-03	6.2E-03
272	TRINITY_DN59306_c0_g3_i1	2.803	2.371	9.5E-05	3.4E-04
273	TRINITY_DN2004_c0_g1_i2	2.801	0.738	5.8E-04	1.6E-03
274	TRINITY_DN4316_c1_g1_i23	2.800	0.188	1.8E-03	4.2E-03
275	TRINITY_DN1875_c0_g2_i10	2.791	0.723	6.1E-04	1.7E-03
276	TRINITY_DN955_c1_g2_i25	2.786	8.138	2.5E-05	1.1E-04
277	TRINITY_DN10_c0_g1_i1	2.760	0.902	6.0E-04	1.6E-03
278	TRINITY_DN2340_c0_g1_i1	2.760	1.632	2.0E-04	6.5E-04
279	TRINITY_DN506_c1_g1_i9	2.755	1.502	2.8E-04	8.8E-04
280	TRINITY_DN6412_c0_g2_i7	2.755	-0.215	3.9E-03	7.7E-03
281	TRINITY_DN57462_c1_g1_i1	2.747	0.687	7.5E-04	2.0E-03
282	TRINITY_DN113_c0_g1_i17	2.743	0.665	7.5E-04	2.0E-03
283	TRINITY_DN1479_c0_g1_i2	2.733	1.494	3.3E-04	9.9E-04
284	TRINITY_DN143_c0_g1_i2	2.727	1.343	3.5E-04	1.0E-03
285	TRINITY_DN76_c0_g1_i6	2.722	0.129	2.6E-03	5.6E-03
286	TRINITY_DN3404_c1_g1_i3	2.719	0.129	2.6E-03	5.6E-03
287	TRINITY_DN10010_c0_g1_i17	2.701	0.829	7.5E-04	2.0E-03
288	TRINITY_DN23087_c0_g3_i2	2.689	0.386	2.1E-03	4.6E-03
289	TRINITY_DN261_c0_g1_i2	2.674	1.309	4.6E-04	1.3E-03
290	TRINITY_DN311_c0_g1_i5	2.672	5.163	5.6E-05	2.1E-04
291	TRINITY_DN3604_c0_g4_i4	2.668	0.823	9.4E-04	2.4E-03
292	TRINITY_DN43236_c0_g1_i8	2.668	3.848	7.3E-05	2.7E-04

293	TRINITY_DN4992_c0_g1_i1	2.647	0.791	9.8E-04	2.5E-03
294	TRINITY_DN2785_c0_g1_i1	2.647	1.526	3.6E-04	1.1E-03
295	TRINITY_DN24110_c0_g1_i3	2.647	0.581	1.2E-03	2.9E-03
296	TRINITY_DN178_c0_g1_i4	2.645	0.349	2.5E-03	5.4E-03
297	TRINITY_DN1460_c0_g1_i1	2.640	8.687	5.5E-05	2.1E-04
298	TRINITY_DN418_c0_g1_i11	2.636	0.339	2.5E-03	5.4E-03
299	TRINITY_DN28_c1_g1_i9	2.633	0.976	9.6E-04	2.4E-03
300	TRINITY_DN21_c0_g1_i4	2.611	0.559	1.4E-03	3.3E-03
301	TRINITY_DN4702_c0_g1_i4	2.601	0.933	1.1E-03	2.6E-03
302	TRINITY_DN10728_c0_g1_i9	2.599	2.977	1.5E-04	5.0E-04
303	TRINITY_DN399_c0_g1_i6	2.598	-0.352	7.2E-03	1.3E-02
304	TRINITY_DN7683_c0_g1_i6	2.589	2.434	2.2E-04	7.0E-04
305	TRINITY_DN1536_c0_g1_i15	2.589	0.755	1.3E-03	3.1E-03
306	TRINITY_DN1819_c0_g5_i3	2.576	-0.370	7.9E-03	1.4E-02
307	TRINITY_DN18706_c0_g1_i22	2.574	-0.002	4.6E-03	9.0E-03
308	TRINITY_DN10771_c0_g1_i15	2.571	3.616	1.4E-04	4.7E-04
309	TRINITY_DN56112_c0_g1_i1	2.558	2.856	2.0E-04	6.5E-04
310	TRINITY_DN83279_c0_g1_i1	2.555	0.265	3.6E-03	7.3E-03
311	TRINITY_DN7932_c0_g2_i3	2.554	0.513	1.8E-03	4.2E-03
312	TRINITY_DN19_c0_g1_i3	2.545	-0.041	5.4E-03	1.0E-02
313	TRINITY_DN74_c0_g1_i2	2.515	0.240	4.3E-03	8.5E-03
314	TRINITY_DN26874_c0_g2_i1	2.497	0.462	2.3E-03	5.0E-03
315	TRINITY_DN10266_c0_g1_i7	2.496	2.516	3.2E-04	9.7E-04
316	TRINITY_DN85_c0_g2_i3	2.491	-0.071	6.7E-03	1.3E-02
317	TRINITY_DN334_c0_g3_i1	2.474	-0.459	1.1E-02	2.0E-02
318	TRINITY_DN44_c0_g1_i1	2.473	-0.086	7.3E-03	1.3E-02
319	TRINITY_DN351_c0_g1_i1	2.457	2.481	3.9E-04	1.1E-03
320	TRINITY_DN178_c0_g1_i34	2.452	-0.103	7.9E-03	1.4E-02
321	TRINITY_DN12573_c0_g1_i1	2.451	1.575	9.1E-04	2.3E-03
322	TRINITY_DN108_c5_g1_i1	2.448	4.010	2.2E-04	7.1E-04
323	TRINITY_DN181_c1_g1_i1	2.440	7.374	1.7E-04	5.4E-04
324	TRINITY_DN955_c1_g2_i33	2.439	9.323	1.6E-04	5.4E-04
325	TRINITY_DN955_c1_g2_i8	2.439	9.323	1.6E-04	5.4E-04
326	TRINITY_DN108_c5_g1_i2	2.433	4.118	2.3E-04	7.2E-04
327	TRINITY_DN18066_c0_g1_i2	2.422	0.951	1.7E-03	3.8E-03
328	TRINITY_DN40630_c0_g1_i1	2.415	6.576	2.0E-04	6.3E-04
329	TRINITY_DN2_c1_g1_i1	2.407	1.419	1.1E-03	2.7E-03
330	TRINITY_DN398_c0_g1_i20	2.404	1.621	8.8E-04	2.2E-03
331	TRINITY_DN38178_c0_g1_i17	2.403	1.063	1.7E-03	3.8E-03
332	TRINITY_DN91_c0_g1_i1	2.400	0.135	6.8E-03	1.3E-02
333	TRINITY_DN300548_c0_g1_i1	2.395	0.372	3.6E-03	7.4E-03
334	TRINITY_DN5090_c6_g1_i1	2.395	1.931	7.7E-04	2.0E-03
335	TRINITY_DN9743_c0_g1_i4	2.392	0.126	6.8E-03	1.3E-02
336	TRINITY_DN581_c0_g1_i2	2.389	2.902	4.3E-04	1.2E-03
337	TRINITY_DN2846_c0_g1_i9	2.383	0.572	3.3E-03	6.9E-03
338	TRINITY_DN28_c1_g1_i10	2.382	1.778	9.9E-04	2.5E-03
339	TRINITY_DN15247_c0_g1_i3	2.363	0.735	3.3E-03	6.7E-03
340	TRINITY_DN696_c0_g1_i7	2.359	0.544	3.7E-03	7.5E-03
341	TRINITY_DN25455_c2_g1_i2	2.352	2.540	6.2E-04	1.7E-03
342	TRINITY_DN24357_c0_g1_i9	2.349	1.575	1.2E-03	2.8E-03
343	TRINITY_DN14474_c0_g1_i22	2.334	0.530	4.1E-03	8.2E-03
344	TRINITY_DN9302_c0_g1_i1	2.331	-0.218	1.3E-02	2.2E-02
345	TRINITY_DN2260_c1_g6_i4	2.330	4.251	3.8E-04	1.1E-03
346	TRINITY_DN42_c0_g1_i17	2.322	0.698	3.9E-03	7.9E-03
347	TRINITY_DN28_c1_g1_i3	2.317	1.337	1.7E-03	3.9E-03

348	TRINITY_DN10123_c1_g1_i1	2.315	0.502	4.3E-03	8.5E-03
349	TRINITY_DN9706_c0_g1_i1	2.289	1.525	1.6E-03	3.6E-03
350	TRINITY_DN1667_c11_g1_i1	2.288	4.458	4.5E-04	1.3E-03
351	TRINITY_DN69_c0_g1_i1	2.286	1.510	1.6E-03	3.6E-03
352	TRINITY_DN53_c0_g1_i2	2.277	1.513	1.7E-03	3.8E-03
353	TRINITY_DN8405_c0_g1_i8	2.275	2.155	1.1E-03	2.6E-03
354	TRINITY_DN179_c0_g1_i26	2.261	5.692	4.6E-04	1.3E-03
355	TRINITY_DN617_c0_g1_i9	2.259	5.268	4.8E-04	1.3E-03
356	TRINITY_DN172904_c0_g1_i1	2.252	1.174	2.3E-03	5.1E-03
357	TRINITY_DN4109_c0_g1_i1	2.241	0.236	7.1E-03	1.3E-02
358	TRINITY_DN981_c0_g1_i1	2.239	1.059	3.4E-03	7.0E-03
359	TRINITY_DN398_c0_g1_i13	2.235	2.120	1.3E-03	3.1E-03
360	TRINITY_DN165365_c0_g1_i1	2.227	0.433	6.3E-03	1.2E-02
361	TRINITY_DN3423_c2_g1_i2	2.225	6.732	5.2E-04	1.4E-03
362	TRINITY_DN116_c0_g1_i1	2.215	0.910	4.0E-03	7.9E-03
363	TRINITY_DN33040_c1_g1_i1	2.209	3.723	7.6E-04	2.0E-03
364	TRINITY_DN3049_c0_g1_i1	2.206	3.562	8.1E-04	2.1E-03
365	TRINITY_DN193_c0_g1_i1	2.203	0.417	7.0E-03	1.3E-02
366	TRINITY_DN1441_c0_g1_i3	2.201	9.024	5.7E-04	1.6E-03
367	TRINITY_DN895_c0_g1_i3	2.194	1.115	3.0E-03	6.2E-03
368	TRINITY_DN22226_c0_g1_i12	2.191	-0.329	2.1E-02	3.4E-02
369	TRINITY_DN64_c0_g2_i2	2.177	9.212	6.5E-04	1.7E-03
370	TRINITY_DN5330_c0_g1_i4	2.163	2.058	1.8E-03	4.2E-03
371	TRINITY_DN23_c0_g1_i23	2.162	0.987	4.6E-03	9.0E-03
372	TRINITY_DN10266_c0_g1_i3	2.156	5.474	7.9E-04	2.0E-03
373	TRINITY_DN617_c0_g1_i1	2.156	3.244	1.1E-03	2.6E-03
374	TRINITY_DN16495_c0_g1_i1	2.135	0.141	1.0E-02	1.8E-02
375	TRINITY_DN394_c0_g1_i12	2.131	0.696	6.0E-03	1.1E-02
376	TRINITY_DN10266_c0_g1_i1	2.127	1.282	3.2E-03	6.6E-03
377	TRINITY_DN52_c0_g1_i24	2.121	2.079	2.2E-03	4.9E-03
378	TRINITY_DN10760_c0_g1_i8	2.111	4.527	1.1E-03	2.6E-03
379	TRINITY_DN58_c0_g1_i10	2.103	2.059	2.4E-03	5.3E-03
380	TRINITY_DN4702_c0_g1_i5	2.099	1.811	2.7E-03	5.7E-03
381	TRINITY_DN127_c0_g2_i8	2.098	1.814	2.7E-03	5.7E-03
382	TRINITY_DN27378_c1_g1_i1	2.096	2.560	1.9E-03	4.3E-03
383	TRINITY_DN55_c0_g1_i1	2.090	0.103	1.2E-02	2.2E-02
384	TRINITY_DN887_c0_g1_i4	2.061	-0.148	1.7E-02	2.8E-02
385	TRINITY_DN329_c0_g1_i24	2.055	1.968	3.0E-03	6.2E-03
386	TRINITY_DN311_c0_g1_i10	2.049	4.633	1.4E-03	3.3E-03
387	TRINITY_DN4821_c0_g1_i3	2.044	1.300	4.6E-03	9.0E-03
388	TRINITY_DN11263_c0_g1_i1	2.038	4.291	1.6E-03	3.6E-03
389	TRINITY_DN5612_c0_g1_i1	2.018	0.257	1.5E-02	2.5E-02
390	TRINITY_DN1438_c0_g3_i1	1.994	0.019	1.7E-02	2.9E-02
391	TRINITY_DN209_c0_g1_i6	1.986	1.843	4.0E-03	7.9E-03
392	TRINITY_DN64_c0_g2_i20	1.973	4.189	2.2E-03	4.8E-03
393	TRINITY_DN24182_c9_g1_i1	1.966	0.555	1.2E-02	2.0E-02
394	TRINITY_DN2453_c0_g1_i5	1.960	1.040	8.3E-03	1.5E-02
395	TRINITY_DN1761_c0_g1_i4	1.952	1.128	6.8E-03	1.3E-02
396	TRINITY_DN459_c0_g1_i2	1.947	-0.008	2.1E-02	3.5E-02
397	TRINITY_DN179_c0_g1_i25	1.946	7.132	2.1E-03	4.6E-03
398	TRINITY_DN1875_c0_g2_i13	1.934	1.014	9.2E-03	1.6E-02
399	TRINITY_DN311_c0_g1_i8	1.928	1.284	7.6E-03	1.4E-02
400	TRINITY_DN50_c0_g1_i1	1.927	0.508	1.3E-02	2.3E-02
401	TRINITY_DN1155_c0_g1_i2	1.919	-0.274	2.7E-02	4.3E-02
402	TRINITY_DN1202_c9_g1_i5	1.912	10.106	2.4E-03	5.2E-03

403	TRINITY_DN1666_c0_g1_i4	1.911	0.888	1.0E-02	1.8E-02
404	TRINITY_DN2822_c0_g2_i1	1.903	0.495	1.5E-02	2.5E-02
405	TRINITY_DN9049_c0_g1_i6	1.902	-0.041	2.6E-02	4.1E-02
406	TRINITY_DN357_c0_g4_i2	1.895	0.327	1.5E-02	2.6E-02
407	TRINITY_DN459_c4_g2_i14	1.882	2.021	5.3E-03	1.0E-02
408	TRINITY_DN955_c1_g2_i4	1.869	9.199	2.9E-03	6.1E-03
409	TRINITY_DN2850_c0_g1_i17	1.860	7.918	3.1E-03	6.4E-03
410	TRINITY_DN3582_c0_g3_i3	1.834	0.696	1.4E-02	2.3E-02
411	TRINITY_DN10029_c0_g1_i1	1.821	0.686	1.4E-02	2.4E-02
412	TRINITY_DN246_c0_g1_i14	1.816	3.917	4.6E-03	9.0E-03
413	TRINITY_DN44_c0_g1_i3	1.815	0.405	2.0E-02	3.3E-02
414	TRINITY_DN112_c1_g2_i26	1.811	1.428	1.1E-02	1.9E-02
415	TRINITY_DN1_c0_g1_i27	1.806	0.400	2.1E-02	3.5E-02
416	TRINITY_DN146_c0_g1_i3	1.799	1.957	7.8E-03	1.4E-02
417	TRINITY_DN5308_c0_g1_i1	1.796	0.245	2.3E-02	3.7E-02
418	TRINITY_DN410_c0_g2_i4	1.787	0.228	2.3E-02	3.7E-02
419	TRINITY_DN0_c5_g2_i1	1.764	0.861	1.8E-02	3.0E-02
420	TRINITY_DN13791_c0_g1_i5	1.745	1.695	1.1E-02	2.0E-02
421	TRINITY_DN137741_c0_g1_i1	1.729	0.474	2.7E-02	4.2E-02
422	TRINITY_DN2821_c0_g1_i2	1.726	0.485	2.8E-02	4.4E-02
423	TRINITY_DN16632_c4_g1_i1	1.706	3.905	7.3E-03	1.3E-02
424	TRINITY_DN4697_c0_g1_i19	1.700	1.328	1.7E-02	2.8E-02
425	TRINITY_DN28_c1_g1_i4	1.676	1.948	1.4E-02	2.3E-02
426	TRINITY_DN2189_c0_g4_i10	1.669	1.987	1.3E-02	2.2E-02
427	TRINITY_DN2_c2_g1_i7	1.660	0.880	2.2E-02	3.6E-02
428	TRINITY_DN10190_c1_g1_i1	1.632	1.655	1.6E-02	2.7E-02
429	TRINITY_DN10266_c0_g1_i12	1.616	1.893	1.7E-02	2.9E-02
430	TRINITY_DN14549_c1_g1_i2	1.615	1.338	2.1E-02	3.4E-02
431	TRINITY_DN165315_c2_g1_i1	1.568	11.712	1.1E-02	2.0E-02
432	TRINITY_DN36611_c0_g1_i1	1.568	4.031	1.3E-02	2.2E-02
433	TRINITY_DN339_c0_g2_i12	1.535	1.572	2.4E-02	3.9E-02
434	TRINITY_DN955_c1_g2_i22	1.526	10.179	1.3E-02	2.3E-02
435	TRINITY_DN80_c1_g1_i2	1.519	4.119	1.6E-02	2.6E-02
436	TRINITY_DN1044_c0_g2_i1	1.502	2.335	2.3E-02	3.7E-02
437	TRINITY_DN131_c0_g1_i10	1.497	2.538	2.1E-02	3.5E-02
438	TRINITY_DN181_c0_g3_i3	1.497	6.984	1.5E-02	2.6E-02
439	TRINITY_DN508_c0_g1_i4	1.428	2.138	3.1E-02	4.8E-02
440	TRINITY_DN329_c0_g1_i13	1.382	3.081	2.9E-02	4.6E-02
441	TRINITY_DN179_c0_g1_i24	1.325	5.810	3.1E-02	4.9E-02
442	TRINITY_DN333_c0_g1_i2	-1.174	4.506	2.9E-02	4.6E-02
443	TRINITY_DN853_c0_g1_i2	-1.177	5.996	2.8E-02	4.4E-02
444	TRINITY_DN1441_c0_g1_i6	-1.209	9.243	2.4E-02	3.8E-02
445	TRINITY_DN85_c2_g1_i5	-1.224	3.427	2.5E-02	3.9E-02
446	TRINITY_DN84_c0_g1_i3	-1.228	1.728	3.1E-02	4.8E-02
447	TRINITY_DN0_c3_g1_i7	-1.231	16.962	2.1E-02	3.5E-02
448	TRINITY_DN160_c0_g1_i15	-1.240	1.427	3.0E-02	4.8E-02
449	TRINITY_DN629_c0_g1_i16	-1.252	12.579	1.9E-02	3.2E-02
450	TRINITY_DN2907_c0_g1_i20	-1.257	1.730	2.5E-02	4.0E-02
451	TRINITY_DN10121_c0_g1_i7	-1.259	1.088	3.0E-02	4.8E-02
452	TRINITY_DN112_c1_g2_i33	-1.262	3.828	2.0E-02	3.3E-02
453	TRINITY_DN57384_c1_g1_i1	-1.266	11.560	1.8E-02	3.0E-02
454	TRINITY_DN8347_c2_g2_i4	-1.268	5.657	1.8E-02	3.0E-02
455	TRINITY_DN112_c1_g2_i7	-1.273	4.586	1.8E-02	3.0E-02
456	TRINITY_DN546_c0_g1_i14	-1.276	1.121	2.7E-02	4.3E-02
457	TRINITY_DN2981_c0_g1_i11	-1.281	0.971	2.7E-02	4.2E-02

458	TRINITY_DN40858_c0_g1_i3	-1.282	0.701	3.1E-02	4.8E-02
459	TRINITY_DN24221_c0_g2_i4	-1.294	2.862	1.8E-02	3.0E-02
460	TRINITY_DN28_c2_g1_i10	-1.297	0.637	3.3E-02	5.1E-02
461	TRINITY_DN306_c0_g1_i2	-1.297	1.048	2.6E-02	4.2E-02
462	TRINITY_DN2_c1_g3_i1	-1.306	3.183	1.7E-02	2.8E-02
463	TRINITY_DN1922_c1_g2_i5	-1.308	1.000	2.5E-02	4.1E-02
464	TRINITY_DN4564_c9_g1_i1	-1.311	4.466	1.5E-02	2.6E-02
465	TRINITY_DN8347_c2_g2_i1	-1.314	1.139	2.5E-02	3.9E-02
466	TRINITY_DN2478_c0_g4_i1	-1.323	12.631	1.3E-02	2.3E-02
467	TRINITY_DN112_c1_g2_i6	-1.327	6.874	1.3E-02	2.3E-02
468	TRINITY_DN8_c0_g1_i19	-1.327	1.905	1.9E-02	3.1E-02
469	TRINITY_DN5774_c1_g1_i1	-1.328	10.417	1.3E-02	2.3E-02
470	TRINITY_DN17650_c0_g1_i3	-1.335	1.331	2.0E-02	3.3E-02
471	TRINITY_DN85_c1_g1_i3	-1.342	2.122	1.6E-02	2.7E-02
472	TRINITY_DN3052_c0_g1_i3	-1.353	2.239	1.5E-02	2.6E-02
473	TRINITY_DN4752_c0_g1_i9	-1.367	0.764	2.0E-02	3.3E-02
474	TRINITY_DN79_c0_g1_i13	-1.372	0.484	2.6E-02	4.1E-02
475	TRINITY_DN8_c17_g1_i21	-1.385	1.599	1.5E-02	2.6E-02
476	TRINITY_DN181_c0_g2_i10	-1.390	0.212	2.6E-02	4.2E-02
477	TRINITY_DN21625_c3_g1_i1	-1.392	2.384	1.3E-02	2.2E-02
478	TRINITY_DN167_c0_g1_i11	-1.395	3.266	1.1E-02	1.9E-02
479	TRINITY_DN7240_c5_g1_i1	-1.400	0.638	2.4E-02	3.8E-02
480	TRINITY_DN2442_c24_g1_i1	-1.401	10.705	8.8E-03	1.6E-02
481	TRINITY_DN19591_c0_g2_i3	-1.414	9.348	8.2E-03	1.5E-02
482	TRINITY_DN10121_c0_g1_i11	-1.415	2.866	9.8E-03	1.7E-02
483	TRINITY_DN1198_c0_g1_i24	-1.421	1.509	1.3E-02	2.3E-02
484	TRINITY_DN2260_c1_g1_i1	-1.426	0.920	1.6E-02	2.6E-02
485	TRINITY_DN112_c0_g1_i17	-1.431	1.812	1.0E-02	1.8E-02
486	TRINITY_DN55_c0_g1_i2	-1.448	0.327	1.8E-02	3.0E-02
487	TRINITY_DN117_c0_g1_i1	-1.449	0.482	1.5E-02	2.6E-02
488	TRINITY_DN78434_c0_g1_i1	-1.452	1.340	1.1E-02	2.0E-02
489	TRINITY_DN9962_c0_g1_i4	-1.453	2.030	9.3E-03	1.7E-02
490	TRINITY_DN4359_c0_g1_i9	-1.463	0.901	1.3E-02	2.3E-02
491	TRINITY_DN46015_c8_g1_i1	-1.467	0.103	2.2E-02	3.5E-02
492	TRINITY_DN211_c0_g2_i1	-1.478	1.192	1.1E-02	2.0E-02
493	TRINITY_DN13545_c0_g2_i9	-1.479	0.408	1.4E-02	2.4E-02
494	TRINITY_DN298394_c0_g1_i1	-1.481	0.872	1.2E-02	2.0E-02
495	TRINITY_DN28_c5_g1_i16	-1.488	3.819	6.1E-03	1.1E-02
496	TRINITY_DN85_c1_g1_i7	-1.491	4.047	5.8E-03	1.1E-02
497	TRINITY_DN8_c0_g1_i39	-1.496	3.249	6.1E-03	1.1E-02
498	TRINITY_DN0_c18_g1_i1	-1.499	12.955	5.1E-03	9.8E-03
499	TRINITY_DN28_c0_g2_i5	-1.500	2.669	6.5E-03	1.2E-02
500	TRINITY_DN122753_c1_g1_i1	-1.505	1.834	7.5E-03	1.4E-02
501	TRINITY_DN28_c0_g1_i10	-1.508	0.880	1.1E-02	2.0E-02
502	TRINITY_DN213_c0_g1_i18	-1.515	1.376	8.4E-03	1.5E-02
503	TRINITY_DN26986_c0_g2_i1	-1.515	0.187	1.9E-02	3.1E-02
504	TRINITY_DN8_c17_g1_i9	-1.516	5.053	4.9E-03	9.4E-03
505	TRINITY_DN112_c46_g1_i1	-1.516	10.031	4.6E-03	9.0E-03
506	TRINITY_DN4093_c0_g1_i4	-1.526	1.325	7.7E-03	1.4E-02
507	TRINITY_DN2442_c0_g1_i5	-1.527	3.001	5.2E-03	1.0E-02
508	TRINITY_DN167_c0_g1_i20	-1.528	2.550	5.5E-03	1.1E-02
509	TRINITY_DN1665_c2_g2_i9	-1.528	2.274	5.8E-03	1.1E-02
510	TRINITY_DN8433_c0_g3_i1	-1.530	6.914	4.3E-03	8.5E-03
511	TRINITY_DN4324_c0_g2_i6	-1.541	1.553	7.5E-03	1.4E-02
512	TRINITY_DN213_c0_g1_i9	-1.544	1.990	5.6E-03	1.1E-02

513	TRINITY_DN112_c1_g2_i15	-1.548	2.105	6.0E-03	1.1E-02
514	TRINITY_DN219_c0_g1_i9	-1.560	0.597	8.0E-03	1.4E-02
515	TRINITY_DN8_c0_g1_i33	-1.560	4.995	3.7E-03	7.5E-03
516	TRINITY_DN8_c17_g1_i22	-1.561	9.805	3.5E-03	7.2E-03
517	TRINITY_DN335_c0_g1_i15	-1.562	0.138	1.2E-02	2.1E-02
518	TRINITY_DN2_c1_g7_i1	-1.562	14.086	3.5E-03	7.2E-03
519	TRINITY_DN2907_c0_g1_i9	-1.566	1.206	6.8E-03	1.3E-02
520	TRINITY_DN878_c0_g1_i6	-1.572	3.717	3.7E-03	7.5E-03
521	TRINITY_DN125_c4_g2_i1	-1.577	2.742	4.2E-03	8.2E-03
522	TRINITY_DN4359_c0_g1_i3	-1.578	1.185	5.6E-03	1.1E-02
523	TRINITY_DN1379_c0_g1_i5	-1.584	0.528	8.2E-03	1.5E-02
524	TRINITY_DN59473_c0_g1_i9	-1.585	2.233	4.2E-03	8.3E-03
525	TRINITY_DN4370_c0_g1_i10	-1.587	2.824	4.0E-03	7.9E-03
526	TRINITY_DN11183_c0_g1_i2	-1.590	1.067	6.3E-03	1.2E-02
527	TRINITY_DN2_c1_g2_i4	-1.593	14.697	2.9E-03	6.1E-03
528	TRINITY_DN1293_c1_g2_i3	-1.596	7.660	2.9E-03	6.1E-03
529	TRINITY_DN210791_c0_g1_i2	-1.599	1.784	4.2E-03	8.3E-03
530	TRINITY_DN15564_c0_g2_i13	-1.609	1.096	5.7E-03	1.1E-02
531	TRINITY_DN13354_c0_g2_i6	-1.612	-0.202	1.2E-02	2.1E-02
532	TRINITY_DN23_c0_g1_i16	-1.615	0.673	6.1E-03	1.1E-02
533	TRINITY_DN167_c0_g1_i42	-1.617	-0.040	9.5E-03	1.7E-02
534	TRINITY_DN8_c17_g1_i1	-1.617	14.177	2.5E-03	5.4E-03
535	TRINITY_DN28_c0_g1_i36	-1.617	1.726	4.4E-03	8.7E-03
536	TRINITY_DN4415_c0_g1_i8	-1.622	0.509	8.2E-03	1.5E-02
537	TRINITY_DN3635_c0_g1_i10	-1.624	-0.043	9.5E-03	1.7E-02
538	TRINITY_DN2102_c0_g2_i1	-1.626	0.851	5.3E-03	1.0E-02
539	TRINITY_DN668_c1_g1_i28	-1.632	-0.046	8.0E-03	1.5E-02
540	TRINITY_DN202_c0_g1_i12	-1.632	0.803	5.3E-03	1.0E-02
541	TRINITY_DN475_c0_g1_i2	-1.633	2.883	2.9E-03	6.1E-03
542	TRINITY_DN2907_c0_g1_i14	-1.638	1.337	4.4E-03	8.7E-03
543	TRINITY_DN28_c0_g1_i22	-1.643	1.655	3.8E-03	7.6E-03
544	TRINITY_DN11557_c0_g1_i1	-1.651	0.795	4.8E-03	9.2E-03
545	TRINITY_DN16140_c2_g1_i1	-1.659	3.657	2.3E-03	5.0E-03
546	TRINITY_DN8_c0_g1_i37	-1.663	4.698	2.0E-03	4.5E-03
547	TRINITY_DN3702_c0_g1_i9	-1.668	1.526	3.6E-03	7.3E-03
548	TRINITY_DN1379_c0_g1_i17	-1.674	0.284	6.1E-03	1.1E-02
549	TRINITY_DN85_c0_g3_i3	-1.675	1.552	3.0E-03	6.2E-03
550	TRINITY_DN6900_c0_g1_i3	-1.676	2.052	2.5E-03	5.4E-03
551	TRINITY_DN2_c377_g1_i1	-1.684	5.735	1.7E-03	3.9E-03
552	TRINITY_DN80_c1_g1_i15	-1.684	-0.281	7.9E-03	1.4E-02
553	TRINITY_DN3003_c0_g1_i10	-1.688	1.168	3.6E-03	7.4E-03
554	TRINITY_DN8690_c4_g1_i1	-1.695	2.656	2.1E-03	4.7E-03
555	TRINITY_DN23087_c1_g2_i1	-1.699	6.932	1.5E-03	3.6E-03
556	TRINITY_DN0_c2_g2_i7	-1.706	16.500	1.4E-03	3.4E-03
557	TRINITY_DN23068_c0_g1_i2	-1.706	0.387	5.3E-03	1.0E-02
558	TRINITY_DN464_c0_g2_i3	-1.706	0.416	5.5E-03	1.1E-02
559	TRINITY_DN10760_c0_g1_i5	-1.718	5.173	1.4E-03	3.3E-03
560	TRINITY_DN213_c0_g1_i8	-1.722	0.572	4.3E-03	8.4E-03
561	TRINITY_DN11013_c0_g1_i7	-1.723	1.347	2.6E-03	5.6E-03
562	TRINITY_DN34_c0_g1_i26	-1.725	0.097	5.0E-03	9.6E-03
563	TRINITY_DN75_c0_g1_i2	-1.734	1.783	2.0E-03	4.4E-03
564	TRINITY_DN28_c3_g2_i2	-1.746	0.562	3.3E-03	6.7E-03
565	TRINITY_DN546_c0_g1_i13	-1.754	0.012	4.2E-03	8.3E-03
566	TRINITY_DN28_c0_g1_i21	-1.756	1.157	2.4E-03	5.1E-03
567	TRINITY_DN25_c0_g1_i3	-1.756	0.396	3.6E-03	7.4E-03

568	TRINITY_DN80_c1_g1_i23	-1.766	0.720	3.1E-03	6.5E-03
569	TRINITY_DN961_c0_g1_i3	-1.767	1.564	1.9E-03	4.3E-03
570	TRINITY_DN58031_c0_g1_i1	-1.768	5.385	1.0E-03	2.5E-03
571	TRINITY_DN7178_c0_g1_i2	-1.769	0.472	3.9E-03	7.9E-03
572	TRINITY_DN16127_c0_g1_i12	-1.770	4.867	1.0E-03	2.5E-03
573	TRINITY_DN113_c0_g1_i32	-1.774	-0.234	5.1E-03	9.8E-03
574	TRINITY_DN629_c0_g1_i15	-1.776	13.752	9.0E-04	2.3E-03
575	TRINITY_DN321_c0_g1_i2	-1.777	-0.034	3.7E-03	7.5E-03
576	TRINITY_DN46015_c3_g1_i1	-1.787	3.739	9.9E-04	2.5E-03
577	TRINITY_DN2102_c0_g2_i2	-1.789	2.899	1.1E-03	2.7E-03
578	TRINITY_DN93_c0_g1_i17	-1.789	2.905	1.1E-03	2.6E-03
579	TRINITY_DN181_c0_g2_i9	-1.797	0.228	3.9E-03	7.7E-03
580	TRINITY_DN125_c1_g1_i11	-1.797	0.436	2.7E-03	5.7E-03
581	TRINITY_DN838_c13_g1_i1	-1.800	1.014	2.1E-03	4.7E-03
582	TRINITY_DN7386_c0_g1_i1	-1.805	4.620	8.1E-04	2.1E-03
583	TRINITY_DN0_c93_g1_i1	-1.814	2.756	1.0E-03	2.5E-03
584	TRINITY_DN167_c0_g1_i25	-1.829	1.144	1.5E-03	3.6E-03
585	TRINITY_DN28_c3_g1_i2	-1.833	0.647	1.8E-03	4.2E-03
586	TRINITY_DN3977_c1_g1_i2	-1.834	2.308	1.1E-03	2.6E-03
587	TRINITY_DN8347_c2_g2_i2	-1.837	8.588	6.0E-04	1.6E-03
588	TRINITY_DN972_c0_g1_i11	-1.841	-0.026	3.3E-03	6.7E-03
589	TRINITY_DN167_c0_g1_i37	-1.845	1.454	1.3E-03	3.1E-03
590	TRINITY_DN2_c6_g1_i3	-1.855	2.618	7.6E-04	2.0E-03
591	TRINITY_DN416_c42_g1_i1	-1.859	1.350	1.0E-03	2.6E-03
592	TRINITY_DN5959_c0_g3_i3	-1.861	0.537	2.2E-03	4.9E-03
593	TRINITY_DN26986_c0_g2_i5	-1.873	0.888	1.6E-03	3.8E-03
594	TRINITY_DN19111_c5_g1_i1	-1.880	2.597	6.6E-04	1.8E-03
595	TRINITY_DN432_c0_g2_i16	-1.885	0.477	1.6E-03	3.8E-03
596	TRINITY_DN2442_c0_g1_i3	-1.892	0.920	1.2E-03	2.9E-03
597	TRINITY_DN3459_c3_g2_i1	-1.902	5.874	4.0E-04	1.1E-03
598	TRINITY_DN1501_c0_g1_i1	-1.907	3.812	4.4E-04	1.3E-03
599	TRINITY_DN32255_c0_g1_i1	-1.909	7.272	3.7E-04	1.1E-03
600	TRINITY_DN125_c0_g1_i11	-1.912	1.948	6.6E-04	1.8E-03
601	TRINITY_DN188_c0_g1_i22	-1.914	0.791	1.1E-03	2.6E-03
602	TRINITY_DN21693_c7_g1_i1	-1.915	6.040	3.6E-04	1.1E-03
603	TRINITY_DN138_c0_g1_i20	-1.916	0.439	1.4E-03	3.4E-03
604	TRINITY_DN246_c0_g1_i20	-1.917	1.556	6.8E-04	1.8E-03
605	TRINITY_DN213_c0_g1_i10	-1.919	0.947	9.7E-04	2.5E-03
606	TRINITY_DN506_c15_g1_i1	-1.926	1.792	5.9E-04	1.6E-03
607	TRINITY_DN312_c0_g1_i24	-1.930	0.073	2.9E-03	6.1E-03
608	TRINITY_DN5774_c1_g2_i1	-1.932	1.524	7.2E-04	1.9E-03
609	TRINITY_DN1441_c0_g1_i11	-1.935	4.074	3.5E-04	1.0E-03
610	TRINITY_DN1436_c0_g1_i2	-1.945	1.114	8.3E-04	2.1E-03
611	TRINITY_DN4370_c0_g1_i1	-1.946	1.062	8.2E-04	2.1E-03
612	TRINITY_DN0_c2_g2_i17	-1.950	13.513	2.7E-04	8.4E-04
613	TRINITY_DN188_c0_g1_i13	-1.952	0.475	1.3E-03	3.2E-03
614	TRINITY_DN85_c1_g1_i12	-1.963	1.220	5.4E-04	1.5E-03
615	TRINITY_DN22624_c0_g1_i11	-1.993	1.879	4.1E-04	1.2E-03
616	TRINITY_DN19591_c0_g2_i14	-1.997	8.930	1.9E-04	6.3E-04
617	TRINITY_DN93_c0_g1_i7	-1.999	0.626	6.5E-04	1.7E-03
618	TRINITY_DN27074_c0_g1_i9	-2.001	-0.335	3.2E-03	6.7E-03
619	TRINITY_DN5_c0_g1_i1	-2.002	1.774	3.8E-04	1.1E-03
620	TRINITY_DN1436_c7_g3_i3	-2.007	0.754	6.9E-04	1.8E-03
621	TRINITY_DN129_c1_g2_i1	-2.025	8.590	1.6E-04	5.2E-04
622	TRINITY_DN8_c0_g1_i29	-2.033	1.699	3.0E-04	9.3E-04

623	TRINITY_DN11134_c13_g1_i1	-2.035	1.893	3.0E-04	9.1E-04
624	TRINITY_DN8690_c3_g1_i1	-2.049	1.171	3.4E-04	1.0E-03
625	TRINITY_DN0_c3_g1_i2	-2.059	8.325	1.2E-04	4.2E-04
626	TRINITY_DN32261_c0_g3_i1	-2.063	2.020	2.4E-04	7.5E-04
627	TRINITY_DN8337_c0_g1_i3	-2.068	0.403	5.7E-04	1.6E-03
628	TRINITY_DN1433_c6_g1_i1	-2.085	5.045	1.1E-04	3.8E-04
629	TRINITY_DN353_c0_g1_i2	-2.087	0.681	3.8E-04	1.1E-03
630	TRINITY_DN26986_c0_g2_i3	-2.102	1.183	3.1E-04	9.6E-04
631	TRINITY_DN0_c7_g1_i6	-2.104	0.562	5.6E-04	1.5E-03
632	TRINITY_DN24930_c2_g1_i2	-2.105	0.857	3.8E-04	1.1E-03
633	TRINITY_DN1245_c2_g3_i1	-2.109	1.035	3.1E-04	9.4E-04
634	TRINITY_DN56_c0_g1_i3	-2.118	-0.069	7.0E-04	1.8E-03
635	TRINITY_DN112_c50_g2_i1	-2.119	7.354	7.9E-05	2.9E-04
636	TRINITY_DN34521_c0_g1_i1	-2.122	7.150	7.7E-05	2.8E-04
637	TRINITY_DN8_c0_g1_i2	-2.125	2.516	1.2E-04	4.2E-04
638	TRINITY_DN18788_c0_g3_i16	-2.128	0.029	5.7E-04	1.6E-03
639	TRINITY_DN417_c1_g1_i1	-2.131	3.874	8.7E-05	3.1E-04
640	TRINITY_DN167_c0_g1_i48	-2.134	3.631	8.8E-05	3.2E-04
641	TRINITY_DN112_c0_g1_i2	-2.142	2.044	1.2E-04	4.1E-04
642	TRINITY_DN125068_c0_g1_i1	-2.143	2.895	9.4E-05	3.4E-04
643	TRINITY_DN253776_c3_g1_i9	-2.150	1.086	1.9E-04	6.1E-04
644	TRINITY_DN45967_c3_g1_i1	-2.155	4.529	6.7E-05	2.5E-04
645	TRINITY_DN85_c1_g1_i6	-2.160	1.686	1.2E-04	4.2E-04
646	TRINITY_DN11_c0_g1_i1	-2.166	0.585	3.5E-04	1.1E-03
647	TRINITY_DN49798_c0_g3_i5	-2.208	0.570	3.5E-04	1.1E-03
648	TRINITY_DN2429_c2_g1_i2	-2.222	3.960	4.3E-05	1.7E-04
649	TRINITY_DN4753_c0_g2_i1	-2.226	0.292	3.9E-04	1.1E-03
650	TRINITY_DN534_c0_g1_i15	-2.236	0.441	1.6E-04	5.3E-04
651	TRINITY_DN131_c0_g1_i16	-2.238	2.658	5.3E-05	2.0E-04
652	TRINITY_DN62_c0_g1_i2	-2.240	1.512	8.8E-05	3.2E-04
653	TRINITY_DN21625_c1_g1_i1	-2.262	3.184	3.6E-05	1.5E-04
654	TRINITY_DN28_c0_g1_i35	-2.263	0.549	1.3E-04	4.5E-04
655	TRINITY_DN28_c0_g1_i7	-2.284	0.733	8.9E-05	3.2E-04
656	TRINITY_DN56812_c0_g1_i10	-2.288	1.961	4.4E-05	1.7E-04
657	TRINITY_DN28_c0_g1_i16	-2.305	0.601	1.0E-04	3.6E-04
658	TRINITY_DN534_c0_g1_i20	-2.317	1.623	5.2E-05	2.0E-04
659	TRINITY_DN2_c0_g2_i7	-2.334	12.603	1.4E-05	6.6E-05
660	TRINITY_DN2_c54_g1_i2	-2.339	0.830	6.6E-05	2.5E-04
661	TRINITY_DN459_c4_g2_i8	-2.354	1.653	2.9E-05	1.2E-04
662	TRINITY_DN506_c7_g2_i1	-2.365	3.060	1.7E-05	7.6E-05
663	TRINITY_DN222_c0_g1_i55	-2.377	1.367	2.8E-05	1.2E-04
664	TRINITY_DN56774_c0_g1_i1	-2.394	1.158	4.6E-05	1.8E-04
665	TRINITY_DN224_c0_g2_i1	-2.397	2.734	1.4E-05	6.6E-05
666	TRINITY_DN2256_c11_g1_i1	-2.408	6.996	7.7E-06	4.1E-05
667	TRINITY_DN10728_c0_g1_i16	-2.420	0.248	5.6E-05	2.1E-04
668	TRINITY_DN5597_c0_g1_i2	-2.422	0.942	3.0E-05	1.2E-04
669	TRINITY_DN112_c1_g3_i2	-2.432	2.242	1.2E-05	6.0E-05
670	TRINITY_DN464_c1_g1_i10	-2.433	0.514	4.5E-05	1.8E-04
671	TRINITY_DN1441_c0_g1_i5	-2.437	5.036	6.6E-06	3.6E-05
672	TRINITY_DN1441_c0_g1_i7	-2.438	3.717	7.6E-06	4.1E-05
673	TRINITY_DN0_c9_g4_i1	-2.472	10.267	4.3E-06	2.5E-05
674	TRINITY_DN64_c0_g2_i8	-2.476	0.783	2.6E-05	1.1E-04
675	TRINITY_DN167_c0_g1_i2	-2.478	2.494	7.7E-06	4.1E-05
676	TRINITY_DN23_c0_g1_i7	-2.486	1.676	1.3E-05	6.1E-05
677	TRINITY_DN7196_c0_g2_i6	-2.496	4.108	4.4E-06	2.5E-05

678	TRINITY_DN0_c2_g2_i20	-2.507	8.680	3.2E-06	2.0E-05
679	TRINITY_DN290_c3_g2_i1	-2.516	1.046	2.3E-05	9.8E-05
680	TRINITY_DN31978_c2_g1_i1	-2.517	4.287	3.8E-06	2.3E-05
681	TRINITY_DN7354_c0_g2_i8	-2.519	0.160	3.9E-05	1.6E-04
682	TRINITY_DN1202_c9_g1_i13	-2.524	1.160	1.5E-05	7.0E-05
683	TRINITY_DN25790_c0_g1_i3	-2.542	0.744	1.5E-05	7.0E-05
684	TRINITY_DN112_c46_g1_i3	-2.553	2.814	3.8E-06	2.3E-05
685	TRINITY_DN6230_c0_g4_i2	-2.563	1.177	9.7E-06	4.9E-05
686	TRINITY_DN1909_c0_g1_i1	-2.566	2.720	3.3E-06	2.1E-05
687	TRINITY_DN8_c0_g1_i10	-2.567	4.991	2.2E-06	1.5E-05
688	TRINITY_DN8417_c7_g1_i1	-2.591	1.367	9.3E-06	4.8E-05
689	TRINITY_DN37194_c0_g1_i5	-2.622	6.905	1.2E-06	8.7E-06
690	TRINITY_DN10747_c0_g1_i10	-2.624	0.234	1.6E-05	7.3E-05
691	TRINITY_DN2_c0_g3_i4	-2.629	10.709	1.1E-06	8.1E-06
692	TRINITY_DN64_c8_g1_i1	-2.645	2.665	2.0E-06	1.4E-05
693	TRINITY_DN853_c1_g1_i1	-2.676	2.515	1.7E-06	1.2E-05
694	TRINITY_DN98_c0_g1_i14	-2.682	0.242	1.0E-05	5.1E-05
695	TRINITY_DN696_c1_g1_i1	-2.717	2.611	1.1E-06	8.2E-06
696	TRINITY_DN20212_c0_g2_i3	-2.720	1.302	1.9E-06	1.3E-05
697	TRINITY_DN0_c7_g1_i15	-2.730	0.550	6.2E-06	3.5E-05
698	TRINITY_DN353_c0_g1_i3	-2.784	2.326	8.5E-07	6.6E-06
699	TRINITY_DN6026_c0_g1_i10	-2.795	0.340	1.4E-05	6.5E-05
700	TRINITY_DN9436_c0_g2_i1	-2.799	8.080	2.3E-07	2.0E-06
701	TRINITY_DN1109_c0_g1_i2	-2.802	0.794	2.0E-06	1.4E-05
702	TRINITY_DN0_c2_g2_i4	-2.802	9.554	2.2E-07	1.9E-06
703	TRINITY_DN384_c0_g1_i4	-2.804	1.140	2.0E-06	1.4E-05
704	TRINITY_DN1665_c2_g2_i6	-2.835	0.720	3.8E-06	2.3E-05
705	TRINITY_DN50908_c0_g1_i2	-2.838	1.484	8.3E-07	6.5E-06
706	TRINITY_DN2429_c2_g1_i1	-2.844	5.233	1.7E-07	1.6E-06
707	TRINITY_DN853_c0_g1_i19	-2.846	0.363	2.6E-06	1.7E-05
708	TRINITY_DN0_c2_g1_i13	-2.847	14.890	1.4E-07	1.4E-06
709	TRINITY_DN9739_c2_g2_i5	-2.863	4.368	1.7E-07	1.6E-06
710	TRINITY_DN108_c2_g3_i2	-2.872	4.621	1.5E-07	1.4E-06
711	TRINITY_DN0_c3_g1_i28	-2.880	3.480	1.6E-07	1.5E-06
712	TRINITY_DN0_c9_g2_i2	-2.885	4.627	1.3E-07	1.3E-06
713	TRINITY_DN85_c2_g2_i1	-2.891	1.045	1.2E-06	9.1E-06
714	TRINITY_DN19591_c9_g1_i1	-2.895	4.308	1.2E-07	1.2E-06
715	TRINITY_DN416_c0_g3_i2	-2.896	8.080	8.9E-08	9.5E-07
716	TRINITY_DN210791_c0_g1_i1	-2.903	3.228	1.6E-07	1.5E-06
717	TRINITY_DN4370_c0_g1_i7	-2.916	2.949	1.6E-07	1.5E-06
718	TRINITY_DN709_c3_g1_i1	-2.934	4.067	8.7E-08	9.4E-07
719	TRINITY_DN28_c0_g1_i41	-2.939	1.283	4.2E-07	3.6E-06
720	TRINITY_DN7607_c0_g1_i1	-2.961	0.987	1.1E-06	8.4E-06
721	TRINITY_DN23_c0_g1_i14	-2.971	1.340	4.5E-07	3.8E-06
722	TRINITY_DN208810_c5_g1_i1	-2.973	1.022	4.6E-07	3.8E-06
723	TRINITY_DN19111_c0_g4_i1	-2.976	2.249	1.7E-07	1.6E-06
724	TRINITY_DN1202_c9_g1_i4	-2.984	9.400	3.7E-08	4.6E-07
725	TRINITY_DN64_c0_g2_i24	-3.001	2.320	8.3E-08	9.1E-07
726	TRINITY_DN224_c0_g1_i45	-3.061	1.594	1.0E-07	1.1E-06
727	TRINITY_DN16649_c0_g4_i6	-3.066	1.587	1.4E-07	1.4E-06
728	TRINITY_DN459_c4_g2_i2	-3.074	4.688	1.9E-08	2.5E-07
729	TRINITY_DN33573_c3_g2_i1	-3.104	2.101	5.0E-08	6.0E-07
730	TRINITY_DN335_c0_g1_i23	-3.110	9.149	1.1E-08	1.5E-07
731	TRINITY_DN335_c12_g1_i1	-3.124	3.538	1.7E-08	2.3E-07
732	TRINITY_DN394_c6_g1_i1	-3.128	5.938	9.6E-09	1.4E-07

733	TRINITY_DN2_c1_g7_i6	-3.152	12.822	6.8E-09	1.1E-07
734	TRINITY_DN4370_c0_g1_i3	-3.161	5.249	7.5E-09	1.1E-07
735	TRINITY_DN8_c0_g1_i13	-3.237	4.071	4.3E-09	7.4E-08
736	TRINITY_DN13140_c7_g1_i1	-3.245	1.750	2.1E-08	2.7E-07
737	TRINITY_DN4222_c0_g2_i5	-3.257	1.107	2.6E-08	3.3E-07
738	TRINITY_DN0_c3_g1_i8	-3.269	3.207	4.5E-09	7.6E-08
739	TRINITY_DN1441_c0_g1_i4	-3.292	5.754	1.8E-09	3.4E-08
740	TRINITY_DN0_c2_g2_i13	-3.308	14.279	1.4E-09	2.7E-08
741	TRINITY_DN45_c0_g1_i3	-3.308	2.006	1.1E-08	1.5E-07
742	TRINITY_DN9462_c5_g1_i1	-3.323	7.716	1.2E-09	2.4E-08
743	TRINITY_DN20209_c8_g1_i1	-3.351	3.006	2.5E-09	4.4E-08
744	TRINITY_DN8_c0_g1_i1	-3.353	1.820	7.0E-09	1.1E-07
745	TRINITY_DN34444_c3_g1_i1	-3.357	4.351	1.3E-09	2.5E-08
746	TRINITY_DN19591_c0_g2_i4	-3.362	3.525	1.6E-09	3.2E-08
747	TRINITY_DN0_c2_g2_i12	-3.380	14.769	6.3E-10	1.4E-08
748	TRINITY_DN302750_c0_g1_i2	-3.395	1.144	7.5E-09	1.1E-07
749	TRINITY_DN6170_c0_g1_i11	-3.418	2.572	1.6E-09	3.1E-08
750	TRINITY_DN0_c3_g1_i27	-3.430	10.663	3.7E-10	8.5E-09
751	TRINITY_DN54816_c0_g1_i1	-3.459	0.927	6.4E-09	1.0E-07
752	TRINITY_DN94_c0_g1_i3	-3.465	0.757	9.7E-09	1.4E-07
753	TRINITY_DN8_c17_g1_i11	-3.483	8.482	2.1E-10	5.1E-09
754	TRINITY_DN459_c4_g2_i15	-3.513	1.836	1.1E-09	2.3E-08
755	TRINITY_DN0_c524_g1_i1	-3.536	6.776	1.2E-10	3.1E-09
756	TRINITY_DN1778_c6_g1_i1	-3.643	2.933	1.4E-10	3.5E-09
757	TRINITY_DN17218_c5_g1_i1	-3.703	1.590	5.1E-10	1.2E-08
758	TRINITY_DN10760_c0_g1_i11	-3.745	6.552	1.2E-11	3.9E-10
759	TRINITY_DN1379_c0_g1_i14	-3.751	1.080	3.4E-10	8.1E-09
760	TRINITY_DN0_c2_g2_i9	-3.762	13.760	9.3E-12	3.0E-10
761	TRINITY_DN9500_c10_g1_i1	-3.790	2.396	6.3E-11	1.7E-09
762	TRINITY_DN24738_c9_g1_i1	-3.832	4.249	7.6E-12	2.5E-10
763	TRINITY_DN34_c0_g1_i24	-3.893	1.555	3.4E-11	9.5E-10
764	TRINITY_DN18783_c1_g5_i1	-3.906	3.547	5.9E-12	2.1E-10
765	TRINITY_DN125_c0_g1_i7	-3.927	1.998	1.6E-11	4.8E-10
766	TRINITY_DN2_c1_g6_i3	-4.013	1.647	1.7E-11	4.9E-10
767	TRINITY_DN6009_c1_g1_i1	-4.030	4.667	7.6E-13	3.1E-11
768	TRINITY_DN93_c0_g1_i30	-4.077	2.216	2.1E-12	7.8E-11
769	TRINITY_DN853_c0_g1_i16	-4.120	6.815	1.6E-13	7.4E-12
770	TRINITY_DN1441_c0_g1_i8	-4.161	1.685	2.6E-12	9.5E-11
771	TRINITY_DN1094_c0_g1_i1	-4.184	4.202	1.7E-13	7.5E-12
772	TRINITY_DN2822_c0_g2_i2	-4.212	6.502	5.9E-14	3.3E-12
773	TRINITY_DN93_c0_g1_i20	-4.263	3.668	1.4E-13	6.8E-12
774	TRINITY_DN1293_c1_g2_i1	-4.292	4.003	5.2E-14	3.0E-12
775	TRINITY_DN475_c0_g1_i13	-4.384	1.593	4.0E-13	1.8E-11
776	TRINITY_DN0_c3_g1_i1	-4.389	5.277	9.6E-15	6.9E-13
777	TRINITY_DN63968_c5_g1_i1	-4.463	2.532	5.2E-14	3.0E-12
778	TRINITY_DN1293_c1_g2_i2	-4.505	2.659	1.5E-14	9.7E-13
779	TRINITY_DN86021_c0_g1_i2	-4.527	3.059	8.5E-15	6.5E-13
780	TRINITY_DN4886_c9_g1_i1	-4.838	4.889	4.8E-17	3.9E-15
781	TRINITY_DN50096_c4_g1_i1	-4.914	5.983	1.3E-17	1.2E-15
782	TRINITY_DN0_c2_g1_i5	-5.085	9.501	1.0E-18	1.1E-16
783	TRINITY_DN5959_c0_g1_i2	-5.116	3.077	8.3E-18	7.8E-16
784	TRINITY_DN0_c119_g1_i2	-5.202	7.669	2.3E-19	2.9E-17
785	TRINITY_DN1766_c8_g1_i1	-5.581	5.071	5.9E-21	1.0E-18
786	TRINITY_DN0_c2_g1_i8	-7.191	12.094	7.3E-31	3.0E-28
787	TRINITY_DN955_c1_g2_i11	-7.906	8.533	7.5E-35	4.6E-32

Table S3. **List of gene transcripts that changed their differential expression by two or more times, in RPE cells at the later stage of retinal regeneration**.

NN	Name	Group_1 L.F.Ch.	Group_2 L.F.Ch.	Group 1	Group 2	Effect
1	TRINITY_DN475_c0_g1_i13	-1.271	-4.384	early down	late down	down
2	TRINITY_DN24738_c9_g1_i1	-1.569	-3.832	early down	late down	down
3	TRINITY_DN1766_c8_g1_i1	-1.580	-5.581	early down	late down	down
4	TRINITY_DN125_c0_g1_i7	-1.674	-3.927	early down	late down	down
5	TRINITY_DN2_c1_g6_i3	-1.775	-4.013	early down	late down	down
6	TRINITY_DN6009_c1_g1_i1	-1.805	-4.030	early down	late down	down
7	TRINITY_DN86021_c0_g1_i2	-1.960	-4.527	early down	late down	down
8	TRINITY_DN0_c119_g1_i2	-2.603	-5.202	early down	late down	down
9	TRINITY_DN5959_c0_g1_i2	-2.734	-5.116	early down	late down	down
10	TRINITY_DN0_c2_g1_i8	-4.948	-7.191	early down	late down	down
11	TRINITY_DN853_c0_g1_i2	-4.171	-6.191	early down	late down	up
12	TRINITY_DN417_c1_g1_i1	-4.215	-5.191	early down	late down	up
13	TRINITY_DN24221_c0_g2_i4	-4.218	-4.191	early down	late down	up
14	TRINITY_DN112_c1_g2_i6	-4.549	-3.191	early down	late down	up
15	TRINITY_DN1433_c6_g1_i1	-4.826	-2.191	early down	late down	up
16	TRINITY_DN1441_c0_g1_i7	-5.241	-1.191	early down	late down	up
17	TRINITY_DN459_c4_g2_i2	-5.303	-0.191	early down	late down	up
18	TRINITY_DN2429_c2_g1_i1	-5.384	0.809	early down	late down	up
19	TRINITY_DN1202_c9_g1_i4	-6.339	1.809	early down	late down	up
20	TRINITY_DN1293_c1_g2_i3	-6.383	2.809	early down	late down	up
21	TRINITY_DN2822_c0_g2_i2	-7.428	3.809	early down	late down	up
22	TRINITY_DN0_c3_g1_i2	-7.899	4.809	early down	late down	up
23	TRINITY_DN955_c1_g2_i3	19.692	22.735	early up	late up	up
24	TRINITY_DN31589_c1_g1_i1	9.589	15.765	early up	late up	up
25	TRINITY_DN37667_c0_g1_i9	8.706	10.747	early up	late up	up
26	TRINITY_DN33040_c1_g1_i2	8.261	10.321	early up	late up	up
27	TRINITY_DN955_c1_g2_i19	5.263	10.959	early up	late up	up
28	TRINITY_DN955_c1_g2_i9	4.714	10.598	early up	late up	up
29	TRINITY_DN443_c0_g1_i6	3.588	6.247	early up	late up	up
30	TRINITY_DN516_c0_g2_i4	3.267	5.381	early up	late up	up
31	TRINITY_DN179_c0_g1_i15	1.758	4.073	early up	late up	up
32	TRINITY_DN129_c2_g1_i2	-1.434	3.600	early down	late up	up
33	TRINITY_DN2_c0_g2_i1	-1.625	4.924	early down	late up	up
34	TRINITY_DN108_c5_g1_i4	-1.635	4.235	early down	late up	up
35	TRINITY_DN2_c94_g3_i1	-1.716	3.841	early down	late up	up
36	TRINITY_DN2260_c1_g6_i2	-1.830	4.081	early down	late up	up
37	TRINITY_DN2260_c1_g6_i4	-2.051	2.330	early down	late up	up
38	TRINITY_DN3423_c2_g1_i2	-2.144	2.225	early down	late up	up
39	TRINITY_DN955_c1_g4_i1	-2.215	4.101	early down	late up	up
40	TRINITY_DN955_c1_g10_i1	-2.215	4.101	early down	late up	up
41	TRINITY_DN1441_c0_g1_i3	-2.627	2.201	early down	late up	up

42	TRINITY_DN617_c0_g1_i9	-3.278	2.259	early down	late up	up
43	TRINITY_DN1202_c9_g1_i5	-3.331	1.912	early down	late up	up
44	TRINITY_DN64_c0_g2_i2	-3.628	2.177	early down	late up	up
45	TRINITY_DN112_c1_g2_i33	4.139	-1.262	early up	late down	down
46	TRINITY_DN0_c3_g1_i27	1.797	-3.430	early up	late down	down
47	TRINITY_DN4564_c9_g1_i1	1.482	-1.311	early up	late down	down

Table S4. Annotated up- and down-regulated genes common to the early and late stages of RPE reprogramming, with the expression changes two-fold or more at the later stage of regeneration.

NN	Name	Group_1 L.F.Ch.	Group_2 L.F.Ch.	Group 1	Group 2	Effect	Sprot_Top_ BLASTX_hit	Gene_ontology_ BLASTX
1	TRINITY_ DN475 _c0_g1_i13	-1.271	-4.384	early down	late down	down	FRIHB_XENLA; Full=Ferritin heavy chain B	ferric iron binding, ferroxidase activity, cellular iron ion homeostasis, iron ion transport
14	TRINITY_ DN112 _c1_g2_i6	-4.549	-2.191	early down	late down	up	PPID BOVIN; Full=Peptidyl-prolyl cis-trans isomerase D	transcription factor binding and heat shock protein binding
17	TRINITY_ DN459 _c4_g2_i2	-5.303	-0.191	early down	late down	up	NCBP3_ HUMAN: Full=Nuclear cap-binding protein subunit 3	plays a key role in mRNA export, serves as adapter protein linking the capped RNAs (m7GpppG-capped RNA) to NCBP1/CBP80.
25	TRINITY_ DN37667 _c0_g1_i9	8.706	10.747	early up	late up	up	TCTP_CHICK; Full= Translationally-controlled tumor protein homolog	Regulator of cellular growth and proliferation. Is part of cytoplasmic microtubules, multifollicular bodies, spindle poles.

26	TRINITY_ DN33040 _c1_g1_i2	8.261	10.321	early up	late up	up	TYB4_MOUSE (Tmsb4x); Full=Thymosin beta-4	Plays an important role in the organization of the cytoskeleton. Binds to and sequesters actin monomers (G actin) and therefore inhibits actin polymerization. Acts by inhibits the entry of hematopoietic pluripotent stem cells into the S-phase.
27	TRINITY_ DN955 _c1_g2_i19	5.263	10.959	early up	late up	up	CC127_ HUMAN; Full=Coiled-coil domain-containing protein 127.	Predicted to be integral component of membrane
29	TRINITY_ DN443 _c0_g1_i6	3.588	6.247	early up	late up	up	F2B_BOVIN (EIF2S2); Full=Eukaryotic translation initiation factor 2 subunit 2.	EIF-2 functions in the early steps of protein synthesis by forming a ternary complex with GTP and initiator tRNA and binding to a 40S ribosomal subunit.
30	TRINITY_ DN516 _c0_g2_i4	3.267	5.381	early up	late up	up	AN32B_ XENTR; Full=Acidic leucine-rich nuclear phosphoprotein 32 family member B.	histone binding and regulation of apoptotic process
31	TRINITY_ DN179 _c0_g1_i15	1.758	4.073	early up	late up	up	COX1_XENLA; Full=Cytochrome c oxidase subunit 1	

Table S5. **Up-regulated annotated genes/proteins when comparing Early vs Norm groups**.

ID	Isoforms	SwissProt ID	Log FCs	Log CPMs	p-Val.	FDRs
**TRINITY_DN10_c0_g1**	i1	RWDD1_HUMAN; RWDD1_RAT	2.563	0.475	0.001	0.004
**TRINITY_DN102_c0_g1**	i1	MYH10_RAT	3.022	2.292	0.000	0.0002
**TRINITY_DN10266_c0_g1**	i10; i7; i1	NU4LM_DANRE; NU4M_CARAU	4.565; 2.654; 1.729	1.909; 2.446; 0.709	6.04E-08; 0.0001; 0.019	9.40E-07; 0.0006; 0.047
**TRINITY_DN10728_c0_g1**	i9	CAPG_BOVIN	1.547	1.799	0.018	0.045
**TRINITY_DN10760_c0_g1**	i8;i2	HBA1_PLEWA; HBB1_TRICR	3.414; 2.273	5.548; 3.506	4.88E-07; 0.0004	5.99E-06; 0.002
**TRINITY_DN11_c0_g1**	i10; i17; i8	PUF60_BOVIN	4.978; 4.189; 3.909	0.189; 0.845; 0.972	9.71E-06; 3.91E-06; 6.10E-06	8.19E-05; 3.73E-05; 5.36E-05
**TRINITY_DN112_c1_g2**	i20; i33; i22	PPID_BOVIN; YCX91_PHAAO	8.382; 4.139; 2.921	0.318; 7.964; 1.165	4.34E-07; 4.20E-09; 0.0002	5.43E-06; 8.36E-08; 0.001
**TRINITY_DN11263_c0_g1**	i10; i15	CYB_RANSI	3.588; 1.952	4.096; 3.271	2.96E-07; 0.002	3.91E-06; 0.007
**TRINITY_DN113_c0_g1**	i31	HMGB3_CHICK	4.159	0.290	0.000	0.0002
**TRINITY_DN119_c0_g1**	i15	K1C15_SHEEP; K1C19_BOVIN; K1C19_POTTR; SFPQ_HUMAN	3.162	-0.612	0.002	0.008
**TRINITY_DN12229_c1_g1**	i5	SPRC_XENLA	2.644	0.107	0.002	0.006
**TRINITY_DN1271_c0_g1**	i5	RAB1A_RAT; TSR1_PONAB; TSR1_XENLA	5.593	0.749	0.000	4.24E-06
**TRINITY_DN131_c0_g1**	i26; i7	K2C4_HUMAN; K2C5_PANTR; K2C6A_HUMAN; K2C7_XENLA;	2.031; 1.896	2.712; 2.369	0.002; 0.003	0.006; 0.011
**TRINITY_DN137_c0_g1**	i1; i2	HABP4_MOUSE	8.976; 2.874	0.889; 1.908	1.55E-08; 7.12E-05	2.81E-07; 0.0004
**TRINITY_DN13791_c0_g1**	i5	MYSU_RABIT	2.002	1.672	0.003	0.010
**TRINITY_DN1393_c0_g1**	i1	FUS_BOVIN; FUS_HUMAN; FUS_MOUSE; RANG_MOUSE	3.711	-0.126	0.000	0.001

**TRINITY_DN14_c1_g1**	i12	NACA_XENTR	7.827	-0.190	0.000	6.71E-05
**TRINITY_DN1411_c0_g1**	i12	MIER1_XENLA	1.867	0.202	0.013	0.034
**TRINITY_DN1460_c0_g1**	i1	NU2M_XENLA	2.349	8.214	0.000	0.001
**TRINITY_DN1479_c0_g1**	i2	PDIA3_CHICK; PDIA3_PONAB	1.968	0.563	0.010	0.027
**TRINITY_DN1479_c0_g2**	i3	PDIA3_BOVIN	2.805	2.584	0.000	0.0003
**TRINITY_DN149_c0_g1**	i4; i5	AKAP9_HUMAN; MYH3_RAT; MYH4_PIG; MYH6_RABIT; MYH6_RAT; MYSS_CYPCA	8.040; 2.546	0.030; -0.257	2.99E-06; 0.004	2.91E-05; 0.013
**TRINITY_DN15_c0_g2**	i6	SF3A3_HUMAN	3.146	0.260	0.000	0.001
**TRINITY_DN1536_c0_g1**	i11	CAPR1_HUMAN; CAPR1_MOUSE	2.993	0.131	0.001	0.003
**TRINITY_DN15937_c0_g1**	i3	MTDC_CHICK	2.399	0.336	0.002	0.008
**TRINITY_DN1625_c0_g2**	i1	VPS29_XENTR	2.264	-0.224	0.008	0.022
**TRINITY_DN1779_c0_g1**	i15	OTOG_HUMAN; PARN_PONAB	2.389	-0.105	0.005	0.015
**TRINITY_DN179_c0_g1**	i21; i11; i7; i15; i26	COX1_ANAPL; COX1_APTAU; COX1_ONCMY; COX1_SALSA; COX1_XENLA	2.831; 2.337; 2.117; 1.758; 1.390	3.207; 5.922; 6.131; 0.600; 4.689	2.92E-05; 0.0002; 0.0007; 0.017; 0.021	0.0002; 0.001; 0.003; 0.042; 0.049
**TRINITY_DN181_c1_g1**	i1	RL22_CHICK	2.638	7.357	0.000	0.0003
**TRINITY_DN18248_c0_g2**	i5	IF4A1_PONAB	2.401	-0.399	0.007	0.021
**TRINITY_DN1875_c0_g2**	i10; i13	EPS15_HUMAN; SET_MOUSE; EPS15_HUMAN; SET_MOUSE	3.037; 1.952	0.704; 0.776	0.0002; 0.008	0.001; 0.023
**TRINITY_DN192_c0_g1**	i3	CALX_HUMAN	7.963	-0.050	0.000	3.83E-05

**TRINITY_DN209344_c0_g1**	i2	S10AA_PIG; S10AA_RABIT	8.520	0.473	0.000	2.91E-06
**TRINITY_DN21_c0_g1**	i4	LC7L3_MOUSE; LC7L3_PONAB	2.357	0.091	0.004	0.012
**TRINITY_DN2189_c0_g4**	i10	PRC2C_HUMAN; PRC2C_MOUSE	1.741	1.799	0.008	0.0220
**TRINITY_DN222_c0_g1**	i57; i50	TC3A_CAEEL	8.584; 7.891	0.543; -0.119	1.57E-07; 6.08E-06	2.22E-06; 5.36E-05
**TRINITY_DN2340_c0_g1**	i1	PSB7_BOVIN	2.263	0.951	0.002	0.009
**TRINITY_DN2429_c0_g1**	i1	BIR_CHICK; PSB4_XENLA	8.197	0.157	0.000	1.30E-05
**TRINITY_DN24357_c0_g1**	i9	RO31_XENLA	1.704	0.757	0.019	0.045
**TRINITY_DN2556_c0_g1**	i10	CAVN1_HUMAN; CAVN1_MOUSE; OBSCN_MOUSE; PHF12_HUMAN; PLEC_HUMAN; PLEC_RAT	3.771	0.838	0.000	0.0001
**TRINITY_DN260_c0_g2**	i1	NU1M_PTEGI	4.095	2.109	0.000	2.91E-06
**TRINITY_DN261_c0_g1**	i2	SNAA_RAT	2.363	0.785	0.002	0.008
**TRINITY_DN28_c0_g3**	i1	DEK_HUMAN	2.206	0.508	0.005	0.016
**TRINITY_DN290_c0_g1**	i15	K2C5_RAT; K2C6A_HUMAN; K2C6A_MOUSE	2.474	1.702	0.000	0.002
**TRINITY_DN29373_c0_g1**	i10	NUCL_XENLA	6.317	1.453	0.000	9.30E-08
**TRINITY_DN30_c0_g1**	i23; i34	KTN1_CHICK; KTN1_HUMAN; KTN1_MOUSE; ZBT7A_CHICK	5.116; 1.631	0.312; 1.157	4.47E-06; 0.017	4.18E-05; 0.042
**TRINITY_DN3049_c0_g1**	i1	YBOX1_XENLA	1.523	2.721	0.015	0.037
**TRINITY_DN3098_c0_g1**	i22	CLH1_BOVIN; CTR9_HUMAN; CTR9_XENTR; MYH10_RAT	8.034	-0.001	0.000	2.76E-05

**TRINITY_DN311_c0_g1**	i3; i6; i10	NU5M_FORLA; NU5M_GADMO; NU5M_ONCMY; NU5M_RABIT	10.488; 1.997; 1.780	2.379; 2.7712; 4.171	1.26E-12; 0.002; 0.004	3.74E-11; 0.007; 0.012
**TRINITY_DN33040_c1_g1**	i2	TYB4_MOUSE	8.261	0.227	0.000	1.01E-05
**TRINITY_DN3519_c2_g1**	i1	UBQ12_ARATH	1.976	1.648	0.003	0.011
**TRINITY_DN37667_c0_g1**	i9	TCTP_CHICK; TCTP_XENTR	8.706	0.641	0.000	1.10E-06
**TRINITY_DN379_c0_g1**	i1	LEG4_RAT	3.350	0.446	0.000	0.0006
**TRINITY_DN381_c0_g1**	i4	SPB10_SORAR	2.043	0.355	0.006	0.019
**TRINITY_DN39414_c0_g1**	i1	HEM0_DELLE; HEM0_MOUSE	2.156	0.291	0.005	0.015
**TRINITY_DN40630_c0_g1**	i2; i3; i1	ATP6_SALSA	2.765; 2.719; 1.596	4.407; 4.765; 5.616	2.72E-05; 3.13E-05; 0.008	0.0002; 0.0002; 0.023
**TRINITY_DN4219_c0_g1**	i1	IRF2_HUMAN	3.386	0.474	0.000	0.0005
**TRINITY_DN42741_c0_g1**	i2	SP16H_MOUSE	2.311	0.253	0.003	0.011
**TRINITY_DN43236_c0_g1**	i8	TPM1_RANTE; TPM3_BOVIN; TPM3_HUMAN; TPM3_MOUSE; TPM3_RAT	1.757	2.798	0.006	0.017
**TRINITY_DN443_c0_g1**	i13;i6	IF2B_BOVIN; IF2B_MOUSE	6.060; 3.588	1.216; 0.302	2.35E-08; 9.48E-05	4.13E-07; 0.0005
**TRINITY_DN4697_c0_g1**	i9	NP1L1_XENTR; NPL1A_XENLA	8.034	-0.005	0.000	2.76E-05
**TRINITY_DN4702_c0_g1**	i1	EF1B_XENLA; EF1B_XENTR	2.930	2.944	0.000	0.0002
**TRINITY_DN474_c1_g1**	i8; i9	TF211_SCHPO; TF28_SCHPO	8.374; 2.924	0.313; 0.374	4.34E-07; 0.0005	5.43E-06; 0.002
**TRINITY_DN4745_c1_g1**	i8	TITIN_HUMAN	2.481	-0.031	0.003	0.011
**TRINITY_DN489_c0_g1**	i18	NEBU_HUMAN	3.703	0.775	0.000	0.0001

**TRINITY_DN516_c0_g2**	i4	AN32B_XENTR	3.267	-0.005	0.000	0.002
**TRINITY_DN53300_c0_g2**	i6	TITIN_HUMAN; TITIN_MOUSE	3.693	-0.123	0.000	0.001
**TRINITY_DN58_c0_g1**	i10	EIF3D_XENLA	2.029	1.758	0.003	0.009
**TRINITY_DN594_c0_g1**	i2	GANAB_HUMAN	3.310	0.954	0.000	0.0003
**TRINITY_DN6480_c1_g1**	i8	1433Z_DROME; 143B1_ONCMY	9.376	1.286	0.000	3.04E-08
**TRINITY_DN65289_c0_g1**	i1	POL_WDSV	2.777	-0.057	0.002	0.006
**TRINITY_DN727_c0_g1**	i2	NDUA4_MOUSE	8.278	0.245	0.000	8.96E-06
**TRINITY_DN7379_c0_g1**	i5	DSC3_BOVIN; EXOS7_MOUSE; GALT3_HUMAN; RADI_MOUSE; REN3B_HUMAN; SRPRB_MOUSE	2.556	-0.259	0.004	0.012
**TRINITY_DN7478_c0_g1**	i1	DDX5_HUMAN; DDX5_MACFA; DDX5_PANTR	3.726	-0.078	0.000	0.001
**TRINITY_DN79_c0_g1**	i14	MUC16_HUMAN	8.455	0.401	0.000	3.91E-06
**TRINITY_DN8_c17_g1**	i13	SPAG1_DANRE	1.861	7.264	0.002	0.008
**TRINITY_DN80_c1_g1**	i8; i33	ACT5_XENLA; ACTA_RAT; ACTB_TRIVU; ACTB_XENLA; ACTB_XENTR; ACTH_RAT; ACTS_RAT	9.799; 3.143	1.707; 3.572	1.06E-10; 4.62E-06	2.56E-09; 4.28E-05
**TRINITY_DN82_c0_g1**	i4; i7; i2	HMGB2_CHICK	3.635; 3.2086; 3.050	5.257; 2.038; 2.459	1.49E-07; 1.48E-05; 1.68E-05	2.15E-06; 0.0001; 0.0001
**TRINITY_DN895_c1_g1**	i14	EF1A_SCHCO; EF1A0_XENLA; EF1A2_XENLA; EF1A3_XENLA	1.876	0.067	0.015	0.039
**TRINITY_DN939_c0_g1**	i5	LORF2_HUMAN	2.928	1.964	0.000	0.0003

**TRINITY_DN955_c1_g2**	i3; i19; i9; i25; i33; i8; i6	CC127_HUMAN	19.692; 5.263; 4.714; 2.271; 2.060; 2.060; 1.757	11.559; 0.466; 3.158; 7.456; 8.765; 8.765; 10.108	4.59E-40; 2.18E-06; 3.19E-09; 0.0002; 0.0009; 0.0009; 0.004	1.86E-37; 2.26E-05; 6.45E-08; 0.001; 0.004; 0.004; 0.012
**TRINITY_DN9706_c0_g1**	i1	TNNC2_PELLE	1.947	0.977	0.007	0.021
**TRINITY_DN9729_c0_g1**	i11	EPIPL_HUMAN; EPIPL_MOUSE	2.214	0.906	0.003	0.0105

83 transcripts were mapped to available protein sequences in the SwissProt database.

Table S6. **Up-regulated annotated genes/proteins, when comparing Late vs Norm groups**.

ID	Isoforms	Proteins	Log FCs	Log CPMs	p-Val.	FDRs
**TRINITY_DN1_c0_g1**	i27	EPIPL_HUMAN; EPIPL_MOUSE	1.806	0.400	0.021	0.035
**TRINITY_DN10_c0_g1**	i1	RWDD1_HUMAN; RWDD1_RAT	2.760	0.902	5.99E-04	0.002
**TRINITY_DN10010_c0_g1**	i17	INO1B_XENLA; PABP1_MOUSE; PABP1_PONAB; PABP1_RAT	2.701	0.829	7.49E-04	0.002
**TRINITY_DN102_c0_g1**	i1	MYH10_RAT	3.872	3.301	2.00E-07	1.80E-06
**TRINITY_DN10266_c0_g1**	i8; i10; i7; i3; i1; i12	NU4LM_DANRE; NU4M_CARAU	10.075; 5.3421; 2.496; 2.156; 2.127; 1.616	1.453; 2.835; 2.516; 5.474; 1.282; 1.893	5.34E-10; 6.47E-10; 0.0003; 0.0008; 0.003; 0.017	1.19E-08; 1.39E-08; 0.001; 0.002; 0.007; 0.029
**TRINITY_DN1070_c0_g1**	i5	SAFB1_PONA; SAFB1_RAT	8.614	0.095	2.70E-06	1.72E-05
**TRINITY_DN10728_c0_g1**	i9	CAPG_BOVIN	2.599	2.977	1.52E-04	5.01E-04
**TRINITY_DN10760_c0_g1**	i8	HBA1_PLEWA; HBB1_TRICR	2.111	4.527	1.06E-03	0.003
**TRINITY_DN10771_c0_g1**	i15	H2AW_MOUSE; IDHP_MOUSE; IDHP_PIG	2.571	3.616	1.39E-04	4.65E-04
**TRINITY_DN11_c0_g1**	i17; i8	PUF60_BOVIN	4.655; 4.510	1.499; 1.742	5.02E-07; 2.16E-07	4.12E-06; 1.90E-06
**TRINITY_DN112_c1_g2**	i20; i22; i26	PPID_BOVIN; YCX91_PHAAO	8.906; 3.114; 1.811	0.375; 1.562; 1.428	5.37E-07; 5.10E-05; 0.011	4.38E-06; 0.0002; 0.019
**TRINITY_DN11263_c0_g1**	i10; i15; i1	CYB_RANSI	4.713; 3.308; 2.038	5.371; 4.736; 4.291	3.42E-10; 1.73E-06;	8.09E-09; 1.23E-05; 0.0036

					0.0016	
**TRINITY_DN113_c0_g1**	i17	HMGB3_CHICK	2.743	0.665	7.48E-04	0.002
**TRINITY_DN115_c0_g1**	i1	NEUL_PONAB	8.336	-0.139	1.13E-05	0.000
**TRINITY_DN1155_c0_g1**	i2	RS9_RAT	1.919	-0.274	0.027	0.043
**TRINITY_DN11654_c0_g1**	i15	MAL_CANLF; SEM6D_PONAB	4.122	0.462	4.37E-05	1.74E-04
**TRINITY_DN11701_c0_g2**	i1	CCD43_CHICK	3.288	0.244	3.52E-04	0.001
**TRINITY_DN119_c0_g1**	i13	K1C15_SHEEP; K1C19_BOVIN; K1C19_POTTR; SFPQ_HUMAN	8.178	-0.293	2.53E-05	1.08E-04
**TRINITY_DN119_c3_g1**	i4	AT1B1_CHICK	9.147	0.587	1.42E-07	1.39E-06
**TRINITY_DN12229_c1_g1**	i5	SPRC_XENLA	3.996	1.570	2.51E-06	1.62E-05
**TRINITY_DN122953_c0_g1**	i1	P53_DANRE	8.630	0.122	2.49E-06	1.61E-05
**TRINITY_DN123222_c0_g1**	i1	PUM1_CHICK	3.330	0.646	1.46E-04	4.87E-04
**TRINITY_DN124361_c0_g1**	i10	AGR2A_XENLA	9.287	0.712	6.15E-08	7.17E-07
**TRINITY_DN12573_c0_g1**	i1	ERVB_TABDI	2.451	1.575	9.12E-04	0.002
**TRINITY_DN127_c0_g2**	i8	YBOX1_XENLA	2.098	1.814	2.70E-03	0.006
**TRINITY_DN1271_c0_g1**	i5	RAB1A_RAT; TSR1_PONAB; TSR1_XENLA	5.882	1.201	6.27E-08	7.24E-07
**TRINITY_DN1273_c0_g1**	i3	HDA1B_XENLA	3.947	0.843	1.25E-05	6.09E-05
**TRINITY_DN13082_c0_g3**	i4	LRP5_HUMAN	3.124	1.708	4.50E-05	1.78E-04
**TRINITY_DN131_c0_g1**	i26; i7; i10	K2C4_HUMAN; K2C5_PANTR; K2C6A_HUMAN; K2C7_XENLA;	3.358; 3.1068; 1.497	4.157; 3.701; 2.538	1.65E-06; 8.45E-06; 0.021	1.18E-05; 4.48E-05; 0.035
**TRINITY_DN13170_c0_g1**	i4	AN32B_BOVIN	3.932	0.310	1.17E-04	4.04E-04
**TRINITY_DN13295_c0_g1**	i26	LRRF1_MOUSE	2.814	0.481	1.10E-03	0.003
**TRINITY_DN137_c0_g1**	i1; i2	HABP4_MOUSE	9.956; 3.202	1.362; 2.436	1.12E-09; 1.33E-05	2.32E-08; 6.41E-05
**TRINITY_DN13791_c0_g1**	i5	MYSU_RABIT	1.745	1.695	0.011	0.020
**TRINITY_DN13924_c0_g**	i7	NAA16_HUMAN;	8.289	-0.181	1.52E-05	7.05E-05

**1**		PCY1A_HUMAN ; PCY1A_RAT				
**TRINITY_DN1393_c0_g1**	i1	FUS_BOVIN; FUS_HUMAN; FUS_MOUSE; RANG_MOUSE	5.188	1.450	1.16E-07	1.20E-06
**TRINITY_DN140_c0_g2**	i1	CTNA1_BOVIN	4.698	0.112	4.27E-05	1.72E-04
**TRINITY_DN1425_c0_g1**	i2	ANX2A_XENLA	8.779	0.250	1.12E-06	8.33E-06
**TRINITY_DN1438_c0_g3**	i1	RHOAA_DANRE	1.994	0.019	0.017	0.029
**TRINITY_DN14474_c0_g1**	i22	HS90B_HUMAN ; HS90B_PONAB; HS90B_XENLA; HSP83_DROMI; MSTN1_XENLA	2.334	0.530	4.11E-03	0.008
**TRINITY_DN14549_c1_g1**	i2	NSA2_BOVIN	1.615	1.338	0.021	0.034
**TRINITY_DN146_c0_g1**	i3	VIM4_XENLA; VIME_BOVIN	1.799	1.957	7.79E-03	0.014
**TRINITY_DN1460_c0_g1**	i1	NU2M_XENLA	2.640	8.687	5.53E-05	0.000
**TRINITY_DN1479_c0_g1**	i2	PDIA3_CHICK; PDIA3_PONAB	2.733	1.494	3.28E-04	0.001
**TRINITY_DN1479_c0_g2**	i3	PDIA3_BOVIN	3.333	3.288	3.17E-06	1.97E-05
**TRINITY_DN149_c0_g1**	i4; i13; i5	AKAP9_HUMAN; MYH3_RAT; MYH4_PIG; MYH6_RABIT; MYH6_RAT; MYSS_CYPCA	9.756; 4.395; 3.396	1.157; -0.163; 0.702	3.80E-09; 0.0002; 0.0001	6.75E-08; 6.36E-04; 3.74E-04
**TRINITY_DN15_c0_g2**	i6	SF3A3_HUMAN	4.344	1.591	5.93E-07	4.77E-06
**TRINITY_DN15247_c0_g1**	i3	MYH10_MOUSE ; MYH9_CHICK; STIP1_XENLA	2.363	0.735	0.003	0.007
**TRINITY_DN1536_c0_g1**	i7; i15	CAPR1_HUMAN ; CAPR1_MOUSE	9.266; 2.589	0.694; 0.755	6.91E-08; 0.001	7.83E-07; 0.003
**TRINITY_DN158_c0_g1**	i3	ZN462_HUMAN	8.196	-0.267	2.28E-05	9.79E-05
**TRINITY_DN1642_c0_g1**	i2	ARC2B_XENLA	8.220	-0.257	2.06E-05	8.93E-05
**TRINITY_DN1666_c0_g1**	i4	SKP1_RAT	1.911	0.888	0.010	0.018
**TRINITY_DN1708_c0_g2**	i2	SEPP1_MOUSE	3.656	0.564	5.63E-05	2.15E-04
**TRINITY_DN1719_c0_g1**	i1	PPME1_RAT	4.894	0.283	1.61E-05	7.34E-05
**TRINITY_DN1779_c0_g1**	i15	OTOG_HUMAN; PARN_PONAB	3.629	1.242	1.91E-05	0.000
**TRINITY_DN179_c0_g1**	i15; i21; i7; i11; i26; i25; i24	COX1_ANAPL; COX1_APTAU; COX1_ONCMY; COX1_SALSA; COX1_XENLA; COX2_XENLA; MUC5A_HUMAN	4.073; 3.897; 3.206; 3.198; 2.2613; 1.946; 1.323	2.952; 4.416; 7.350; 6.929; 5.692; 7.132; 5.810	1.09E-07; 7.09E-08; 2.24E-06; 2.40E-06;	1.14E-06; 7.96E-07; 1.50E-05; 1.57E-05; 0.001; 0.005; 0.049

					0.0005; 0.002; 0.031	
**TRINITY_DN18066_c0_g1**	i2	PR38A_PONAB	2.422	0.951	0.002	0.004
**TRINITY_DN181_c1_g1**	i1	RL22_CHICK	2.440	7.374	1.66E-04	0.001
**TRINITY_DN1810_c0_g1**	i1	FBP1L_HUMAN	3.319	-0.252	0.002	0.004
**TRINITY_DN1812_c0_g1**	i5	SEPT8_XENTR	2.949	1.409	1.22E-04	4.16E-04
**TRINITY_DN18248_c0_g2**	i5	IF4A1_PONAB	3.119	0.484	4.28E-04	1.22E-03
**TRINITY_DN18706_c0_g1**	i22	CSDE1_HUMAN	2.574	-0.002	0.005	9.00E-03
**TRINITY_DN18749_c0_g1**	i2	GSE1_HUMAN	4.753	0.159	3.38E-05	1.40E-04
**TRINITY_DN18749_c0_g2**	i3	R3HD1_HUMAN; STX16_HUMAN	3.184	0.143	5.79E-04	0.002
**TRINITY_DN1875_c0_g2**	i10; i13	EPS15_HUMAN; SET_MOUSE	2.791; 1.934	0.723; 1.014	0.0006; 0.009	0.0016; 0.016
**TRINITY_DN18777_c0_g1**	i1	CAST2_XENLA	8.636	0.125	2.29E-06	1.51E-05
**TRINITY_DN192_c0_g1**	i3	CALX_HUMAN	9.001	0.466	3.30E-07	2.83E-06
**TRINITY_DN197_c0_g1**	i16	6PGD_HUMAN; 6PGD_SHEEP; ATRX_MOUSE; ATRX_PANTR; CHD5_RAT; SNW1_HUMAN	8.820	0.281	8.95E-07	6.89E-06
**TRINITY_DN208_c0_g1**	i11	HA10_MOUSE; HA1Y_MOUSE; HMR1_MOUSE; HMR1_PONAB	3.645	0.558	6.32E-05	2.38E-04
**TRINITY_DN21_c0_g1**	i4	LC7L3_MOUSE; LC7L3_PONAB	2.611	0.559	0.001	0.003
**TRINITY_DN2189_c0_g4**	i10	PRC2C_HUMAN; PRC2C_MOUSE	1.669	1.987	0.013	0.022
**TRINITY_DN2215_c1_g2**	i12	CDV3_XENTR; NUDT9_HUMAN; PSA5_MOUSE	2.838	0.763	0.001	0.001
**TRINITY_DN222_c0_g1**	i30; i64	TC3A_CAEEL	8.6375295647; 3.6675953295	0.1335823528; 1.2494094255	2.29E-06; 1.53E-05	1.51E-05; 7.05E-05
**TRINITY_DN23087_c0_g3**	i2	MYH11_HUMAN; MYH9_MOUSE	2.689	0.386	0.002	0.005
**TRINITY_DN2340_c0_g1**	i1	PSB7_BOVIN	2.760	1.632	2.03E-04	6.50E-04
**TRINITY_DN2341_c0_g2**	i15; i4	DJC25_XENLA; MYH10_HUMAN	8.032; 4.581	-0.422; 0.004	4.82E-05; 7.54E-05	0.0002; 0.0003
**TRINITY_D**	i2	LA_BOVIN;	3.076	0.440	5.12E-04	1.42E-03

**N2367_c0_g1**		LA_HUMAN				
**TRINITY_DN241_c1_g1**	i10	K1C10_BOVIN; K1C10_RAT; K1C13_HUMAN; K1C17_HUMAN; K1C4_XENLA	8.467	-0.026	5.91E-06	3.33E-05
**TRINITY_DN24110_c0_g1**	i3	PPIB_HUMAN	2.647	0.581	1.19E-03	0.003
**TRINITY_DN2412_c0_g1**	i3	ZO1_MOUSE	3.005	0.367	6.95E-04	0.002
**TRINITY_DN24357_c0_g1**	i9	RO31_XENLA	2.349	1.575	1.15E-03	0.003
**TRINITY_DN246_c0_g1**	i7; i14	EF2_CALJA; EF2_CHICK; EF2_MOUSE; EF2_RAT	8.677; 1.816	0.158; 3.917	1.95E-06; 0.005	1.35E-05; 0.009
**TRINITY_DN253871_c0_g1**	i1	DHX15_HUMAN	8.042	-0.415	4.82E-05	0.000
**TRINITY_DN25407_c0_g1**	i6	DESP_HUMAN	8.394	-0.093	8.53E-06	4.48E-05
**TRINITY_DN254135_c0_g1**	i1	INADL_CANLF	3.679	0.060	3.73E-04	1.10E-03
**TRINITY_DN2556_c0_g1**	i10	CAVN1_HUMAN; CAVN1_MOUSE; OBSCN_MOUSE; PHF12_HUMAN; PLEC_HUMAN; PLEC_RAT	3.710	1.015	2.08E-05	9.01E-05
**TRINITY_DN2566_c0_g1**	i10	IPP2_MOUSE; SCFD1_HUMAN; SCFD1_MOUSE	9.831	1.226	2.39E-09	4.36E-08
**TRINITY_DN260_c0_g2**	i1	NU1M_PTEGI	3.584	1.824	6.48E-06	3.59E-05
**TRINITY_DN261_c0_g1**	i2	SNAA_RAT	2.674	1.309	4.58E-04	1.30E-03
**TRINITY_DN2616_c0_g1**	i1	TITIN_HUMAN	8.256	-0.217	1.68E-05	7.62E-05
**TRINITY_DN26874_c0_g2**	i1	SKIL_HUMAN; SKIL_MOUSE; SKIL_PONAB	2.497	0.462	0.002	0.005
**TRINITY_DN2785_c0_g1**	i1	PSB7_HUMAN	2.647	1.526	3.58E-04	1.06E-03
**TRINITY_DN28_c0_g3**	i1	DEK_HUMAN	3.699	2.095	2.86E-06	1.80E-05
**TRINITY_DN2846_c0_g1**	i7; i5; i4; i9	MYH1B_CHICK; MYH2_BOVIN; MYH4_MOUSE; MYH4_PIG; MYH7_PAPHA; MYH8_RAT	9.641; 9.270; 4.217; 2.383	1.058; 0.701; 1.482; 0.572	7.81E-09; 6.91E-08; 1.25E-06; 0.003	1.17E-07; 7.83E-07; 9.05E-06; 0.007
**TRINITY_DN2846_c0_g4**	i1	MYH1B_CHICK	8.226	-0.250	1.86E-05	8.15E-05
**TRINITY_DN2859_c0_g1**	i5; i6; i1; i15; i15	GNL1_MACMU; IPO7_HUMAN; PDIA5_HUMAN;	3.319; 3.047	-0.25; -0.481	0.002; 0.003	0.004; 0.006

		PSMD6_BOVIN; SMCA2_HUMAN; UBC9_XENTR; ZHX1_PONPY				
**TRINITY_DN290_c0_g1**	i15; i19	K2C5_RAT; K2C6A_HUMAN; K2C6A_MOUSE	3.269; 3.263	2.660; 2.397	7.18E-06; 9.40E-06	3.90E-05; 4.84E-05
**TRINITY_DN29373_c0_g1**	i15; i10	NUCL_XENLA	11.144; 7.585	2.486; 2.831	5.54E-13; 1.32E-12	2.34E-11; 5.04E-11
**TRINITY_DN30_c0_g1**	i23	KTN1_CHICK; KTN1_HUMAN; KTN1_MOUSE; ZBT7A_CHICK	4.988	0.385	9.84E-06	5.02E-05
**TRINITY_DN300430_c0_g1**	i1	TITIN_HUMAN	3.004	-0.002	0.001	0.003
**TRINITY_DN3038_c0_g1**	i1	IGJ_EQUAS	4.629	1.454	5.74E-07	0.000
**TRINITY_DN3049_c0_g1**	i1	YBOX1_XENLA	2.206	3.562	8.06E-04	0.002
**TRINITY_DN31_c0_g1**	i7	IF4G2_PONAB; IF4G2_RABIT	9.661	1.071	6.80E-09	1.07E-07
**TRINITY_DN311_c0_g1**	i3; i4; i6; i5; i10; i8	NU5M_FORLA; NU5M_GADMO; NU5M_ONCMY; NU5M_RABIT	11.372; 9.365; 3.085; 2.672; 2.049; 1.928	2.708; 0.779; 3.984; 5.163; 4.633; 1.284	1.22E-13; 3.93E-08; 7.96E-06; 5.61E-05; 0.001; 0.008	6.49E-12; 4.81E-07; 4.25E-05; 0.0002; 0.003; 0.014
**TRINITY_DN3178_c0_g1**	i1	TITIN_HUMAN	3.881	0.789	1.78E-05	7.95E-05
**TRINITY_DN32007_c0_g1**	i5	AKP8L_HUMAN; AKP8L_MOUSE; KAP2_PIG	9.262	0.690	7.32E-08	8.14E-07
**TRINITY_DN329_c0_g1**	i24; i13	K1C0_XENLA; K1C12_MOUSE; K1C15_SHEEP; K1C17_PANTR; K1C17_RAT; K1C24_HUMAN	2.055; 1.382	1.968; 3.081	0.003; 0.029	0.006; 0.046
**TRINITY_DN33040_c1_g1**	i2; i1	TYB4_MOUSE	10.321; 2.209	1.694; 3.723	1.13E-10; 0.0008	2.95E-09; 0.002
**TRINITY_DN341_c0_g1**	i4; i7; i2	EIF3L_HUMAN	8.972; 8.003; 3.427	0.426; -0.442; -0.143	3.79E-07; 6.04E-05; 0.001	3.22E-06; 0.0002; 0.003
**TRINITY_DN34364_c0_g1**	i2	RYR1_RABIT	5.487	0.832	6.37E-07	5.06E-06
**TRINITY_DN357_c0_g4**	i2	ICAL_PIG	1.895	0.327	0.015	0.026
**TRINITY_DN35997_c0_g3**	i2	MYSS_CYPCA	4.272	-0.266	3.47E-04	1.04E-03
**TRINITY_DN3604_c0_g4**	i4	RAB2A_CHICK; RAB2A_MOUSE	2.668	0.823	9.40E-04	0.002

**TRINITY_DN37667_c0_g1**	i9	TCTP_CHICK; TCTP_XENTR	10.747	2.106	7.35E-12	2.50E-10
**TRINITY_DN379_c0_g1**	i1	LEG4_RAT	4.285	1.526	8.14E-07	6.39E-06
**TRINITY_DN38178_c0_g1**	i16; i17	DNAJ1_CHLAE; DNJA1_PONAB; S17A5_HUMAN	3.481; 2.403	0.783; 1.063	6.75E-05; 0.002	0.0002; 0.004
**TRINITY_DN3885_c0_g1**	i4	AN32A_BOVIN	4.879	0.273	1.73E-05	7.77E-05
**TRINITY_DN3920_c0_g1**	i2	RTF1_MOUSE	8.327	-0.152	0.000	5.64E-05
**TRINITY_DN398_c0_g1**	i20; i13	HSP7C_CHICK; HSP7C_ICTPU; HSP7C_PONAB; HSP7C_RAT; HSP7C_SAGOE	2.404; 2.235	1.621; 2.120	0.0009; 0.001	0.002; 0.003
**TRINITY_DN399_c0_g1**	i6	STAU1_HUMAN	2.598	-0.352	0.007	0.013
**TRINITY_DN40630_c0_g1**	i3; i2; i1	ATP6_SALSA	3.633; 2.915; 2.415	5.826; 4.745; 6.575	2.09E-07; 1.59E-05; 0.0002	1.87E-06; 7.31E-05; 0.0006
**TRINITY_DN4172_c0_g2**	i1; i12; i10; i14	ILF2_PONAB; MK16B_XENLA; TR150_HUMAN; TR150_XENLA	8.1467; 7.911; 4.176; 3.084	-0.314; -0.528; 0.519; 0.736	2.81E-05; 8.54E-05; 3.42E-05; 0.0003	0.0001; 0.0003; 0.0001; 0.001
**TRINITY_DN418_c0_g1**	i3; i11	FOXK2_MOUSE	2.830; 2.636	0.506; 0.339	0.001; 0.002	0.003; 0.005
**TRINITY_DN42_c0_g1**	i17	K2C7_BOVIN; K2C7_XENLA	2.322	0.698	0.004	0.008
**TRINITY_DN42741_c0_g1**	i2	SP16H_MOUSE	3.543	1.611	0.000	5.13E-05
**TRINITY_DN43236_c0_g1**	i8	TPM1_RANTE; TPM3_BOVIN; TPM3_HUMAN; TPM3_MOUSE; TPM3_RAT	2.668	3.848	7.35E-05	2.72E-04
**TRINITY_DN44_c0_g1**	i1; i3	AT2A1_RABIT; AT2A1_RAT;	2.473; 1.815	-0.086; 0.405	0.007; 0.020	0.013; 0.033
**TRINITY_DN443_c0_g1**	i13; i6	IF2B_BOVIN; IF2B_MOUSE	7.638; 6.247	2.882; 3.010	9.31E-13; 2.36E-11	3.68E-11; 6.87E-10
**TRINITY_DN459_c4_g2**	i3; i14	NCBP3_HUMAN	4.014; 1.882	1.277; 2.021	3.90E-06; 0.005	2.32E-05; 0.010
**TRINITY_DN4697_c0_g1**	i18; i19	NP1L1_XENTR; NPL1A_XENLA	8.630; 1.699	0.126; 1.327	2.49E-06; 0.017	1.61E-05; 0.028
**TRINITY_DN4702_c0_g1**	i1; i4; i5	EF1B_XENLA; EF1B_XENTR	3.733; 2.600; 2.099	3.905; 0.933; 1.811	2.34E-07; 0.001; 0.003	2.03E-06; 0.003; 0.006
**TRINITY_DN4821_c0_g1**	i3	ZN326_BOVIN	2.044	1.300	0.005	0.009
**TRINITY_DN50_c0_g1**	i1	CALM_STIJA; CAM2B_XENLA	1.927	0.508	0.013	0.023
**TRINITY_DN501_c0_g1**	i1	NEBU_HUMAN	3.093	0.462	0.000	0.001

**TRINITY_DN50515_c0_g1**	i1	COX2_XENLA	3.889	0.255	0.000	0.000
**TRINITY_DN508_c0_g1**	i4	CALR_HUMAN; CALR_MACFU	1.428	2.138	0.031	0.048
**TRINITY_DN516_c0_g2**	i4	AN32B_XENTR	5.381	2.183	6.08E-09	9.93E-08
**TRINITY_DN52_c0_g1**	i10; i24	A2MG_HUMAN; MUG2_RAT; OVOS_CHICK; OVOS_MOUSE; PZP_HUMAN	8.482; 2.121	-0.015; 2.079	5.40E-06; 0.002	3.08E-05; 0.0049
**TRINITY_DN5308_c0_g1**	i6; i1	SEC62_HUMAN; SEC62_MOUSE	4.910; 1.796	0.2979; 0.244	1.50E-05; 0.0223	7.04E-05; 0.038
**TRINITY_DN5397_c0_g1**	i9	ENPL_CHICK; ENPL_MESAU	8.316	-0.168	1.25E-05	6.09E-05
**TRINITY_DN55_c0_g1**	i1	RL23_RAT	2.090	0.103	0.012	0.022
**TRINITY_DN552_c0_g1**	i1	CCS_PIG; LSM14_PLEWA	8.795	0.266	9.64E-07	7.33E-06
**TRINITY_DN57462_c1_g1**	i1	PPAC_MOUSE; PPAC_RAT	2.747	0.687	0.001	1.95E-03
**TRINITY_DN5782_c0_g1**	i3	VATG1_BOVIN	4.724	0.150	3.95E-05	1.60E-04
**TRINITY_DN58_c0_g1**	i10	EIF3D_XENLA	2.103	2.059	0.002	0.005
**TRINITY_DN5827_c0_g1**	i18	AKAP9_RABIT; TCPE_MACFA; TCPE_PONAB	8.396	-0.097	8.53E-06	4.48E-05
**TRINITY_DN59306_c0_g1**	i2	KLC1_RAT	3.174	0.520	3.19E-04	9.71E-04
**TRINITY_DN594_c0_g1**	i2	GANAB_HUMAN	2.990	0.889	2.26E-04	7.14E-04
**TRINITY_DN652_c0_g1**	i19	CLUS_COTJA	3.605	0.009	0.001	0.002
**TRINITY_DN6763_c2_g1**	i2	KCRM_HUMAN; KCRM_PIG; KCRM_RAT	9.138	0.576	1.42E-07	1.39E-06
**TRINITY_DN6863_c0_g1**	i1	SIN3A_MOUSE	8.546	0.044	3.80E-06	2.29E-05
**TRINITY_DN6930_c0_g1**	i16	ANXA1_CAVCU; ANXA1_RABIT	2.972	0.342	0.001	0.002
**TRINITY_DN705_c0_g1**	i11	TBA2_DROME; TBA_LEPDS; TBA_LYTPI; TBA_TORMA	8.437	-0.063	6.47E-06	3.59E-05
**TRINITY_DN72_c0_g1**	i1	ZNFX1_HUMAN	4.310	-0.234	0.000	0.001
**TRINITY_DN7205_c0_g1**	i2; i18	LC7L3_PONAB; NMI_BOVIN; PPM1G_BOVIN; PPM1G_MOUSE; RN213_HUMAN; RN213_MOUSE	4.350; 3.934	-0.192; 0.307	0.0002; 0.0001	0.0007; 0.0004
**TRINITY_DN727_c0_g1**	i2	NDUA4_MOUSE	8.735	0.223	1.42E-06	1.02E-05
**TRINITY_DN7288_c0_g1**	i16	CATL_DROME; GTPB4_MOUSE; MYLK_MELGA; RAI14_MOUSE;	3.029	0.020	0.001	2.95E-03

		RAI14_RAT				
**TRINITY_DN7337_c0_g1**	i1	TSYL2_MOUSE	5.255	0.615	2.28E-06	1.51E-05
**TRINITY_DN73384_c0_g1**	i1	KGUA_HUMAN	3.298	-0.264	0.002	0.005
**TRINITY_DN7379_c0_g1**	i9; i14	DSC3_BOVIN; EXOS7_MOUSE; GALT3_HUMAN; RADI_MOUSE; REN3B_HUMAN; SRPRB_MOUSE	8.667; 4.964	0.147; 0.357	1.95E-06; 1.13E-05	1.35E-05; 5.64E-05
**TRINITY_DN74_c0_g1**	i2	SPSY_BOVIN	2.515	0.240	0.004	0.009
**TRINITY_DN77822_c0_g1**	i2	GLO2_DANRE	3.120	-0.421	0.002	0.005
**TRINITY_DN7932_c0_g2**	i3	SRS1A_DANRE; SRSF1_CHICK	2.554	0.513	0.002	0.004
**TRINITY_DN80_c1_g1**	i33; i2	ACT5_XENLA; ACTA_RAT; ACTB_TRIVU; ACTB_XENLA; ACTB_XENTR; ACTH_RAT; ACTS_RAT	3.568; 1.520	4.169; 4.119	5.01E-07; 0.015	4.12E-06; 0.026
**TRINITY_DN82_c0_g1**	i4; i2; i7	HMGB2_CHICK	3.868; 2.934; 2.930	5.665; 2.565; 1.999	5.23E-08; 3.93E-05; 7.83E-05	6.22E-07; 0.0002; 0.0003
**TRINITY_DN851_c0_g1**	i14	IGK_HUMAN	3.224	0.188	0.001	0.001
**TRINITY_DN8721_c0_g1**	i9	GNAI2_CAVPO; GNAI2_MOUSE; MPRD_RAT	8.375	-0.117	9.37E-06	4.84E-05
**TRINITY_DN873_c0_g1**	i18	AHNK_HUMAN	9.866	1.268	1.94E-09	3.61E-08
**TRINITY_DN887_c0_g1**	i4	RUXF_XENLA	2.061	-0.148	0.017	0.028
**TRINITY_DN9_c0_g1**	i3	NOL8_HUMAN	4.943	0.328	1.21E-05	5.96E-05
**TRINITY_DN90912_c0_g1**	i1	TOM70_HUMAN	3.798	0.170	0.000	7.03E-04
**TRINITY_DN939_c0_g1**	i5	LORF2_HUMAN	4.337	3.506	1.45E-08	1.91E-07
**TRINITY_DN955_c1_g2**	i3; i19; i9; i20; i25; i33; i8; i4; i22	CC127_HUMAN	22.735; 10.959; 10.598; 3.447; 2.786; 2.439; 1.526	14.02; 6.160; 9.165; 0.754; 8.138; 9.323; 9.199; 10.179	7.08E-47; 1.42E-22; 1.36E-26; 8.25E-05; 2.46E-05; 0.0002; 0.003; 0.013	8.66E-44; 2.91E-20; 4.18E-24; 0.0003; 0.0001; 0.0005; 0.006; 0.023
**TRINITY_DN9706_c0_g1**	i1	TNNC2_PELLE	2.289	1.525	0.002	0.004

**TRINITY_DN972_c0_g1**	i3	ANXA1_CHICK; ANXA1_HORSE; ANXA1_HUMAN; ANXA1_RABIT	8.491	-0.018	4.94E-06	2.84E-05

174 transcripts were mapped to available protein sequences in the SwissProt database.

Table S7. Down-regulated annotated genes/proteins when comparing Early vs Norm groups.

ID	Isoforms	SwissProt ID	logFCs	Log CPMs	PValues	FDRs
**TRINITY_DN1046_c0_g1**	i2	ATP5H_HUMAN	-2.158	-0.586	4.16E-04	1.92E-03
**TRINITY_DN11_c0_g1**	i1	PUF60_BOVIN	-1.808	0.345	8.20E-04	3.42E-03
**TRINITY_DN1109_c0_g1**	i2	RL38_RAT	-2.443	0.477	5.64E-06	5.07E-05
**TRINITY_DN112_c1_g2**	i21; i30; i36; i28; i6	PPID_BOVIN; YCX91_PHAAO	-1.604; -1.763; -1.976; -3.702; -4.548	1.367; 0.503; 0.251; 1.476; 5.458	0.002; 0.001; 0.0002; 9.51E-13; 1.34E-20	0.007; 0.005; 0.001; 2.89E-11; 1.01E-18
**TRINITY_DN119_c0_g1**	i5	K1C15_SHEEP; K1C19_BOVIN; K1C19_POTTR; SFPQ_HUMAN	-1.840	-0.422	2.1E-03	7.71E-03
**TRINITY_DN1198_c0_g1**	i24	RACK1_RAT; SMUF1_HUMAN; SMUF1_MOUSE	-1.756	0.949	8.0E-04	3.43E-03
**TRINITY_DN125068_c0_g1**	i1	RL37A_XENLA	-1.282	2.806	1.0E-02	2.75E-02
**TRINITY_DN131_c0_g1**	i16	K2C4_HUMAN; K2C5_PANTR; K2C6A_HUMAN; K2C7_XENLA	-3.383	1.814	2.74E-11	7.08E-10
**TRINITY_DN134_c1_g1**	i32	LGMN_BOVIN; LGMN_MACFA; RLA0_LITSY; RLA0_RAT	-1.711	1.900	7.57E-04	3.20E-03
**TRINITY_DN15564_c0_g2**	i13	AAKG1_HUMAN; G3P_CHICK; G3P_FELCA	-1.822	0.605	8.69E-04	3.54E-03
**TRINITY_DN16127_c0_g1**	i12	DAA1A_XENLA	-1.246	4.643	1.12E-02	3.04E-02
**TRINITY_DN163_c0_g1**	i1	H4_XENTR	-1.592	0.266	4.33E-03	1.33E-02

**TRINITY_DN17650_c0_g1**	i3	RS12_CHICK; RS12_PIG	-3.250	0.226	5.36E-09	1.02E-07
**TRINITY_DN18788_c0_g3**	i4	MUC2_HUMAN; MUC5B_CHICK; VWF_CANLF	-2.321	0.053	5.22E-05	3.35E-04
**TRINITY_DN1909_c0_g1**	i1	RL30_OPHHA	-1.309	2.744	8.98E-03	2.49E-02
**TRINITY_DN210791_c0_g1**	i1	RL37_RAT	-1.193	3.384	1.61E-02	4.02E-02
**TRINITY_DN212658_c0_g1**	i1	RL40_SHEEP	-2.619	-0.049	4.90E-06	4.47E-05
**TRINITY_DN222_c0_g1**	i55	TC3A_CAEEL	-1.640	1.224	1.70E-03	6.29E-03
**TRINITY_DN22624_c0_g1**	i11	ADT2_MOUSE; ADT2_TACAC	-1.952	1.443	1.43E-04	7.79E-04
**TRINITY_DN246_c0_g1**	i20	EF2_CALJA; EF2_CHICK; EF2_MOUSE; EF2_RAT	-1.373	1.381	8.94E-03	2.49E-02
**TRINITY_DN2925_c0_g1**	i4	RL31_RAT	-2.117	0.410	8.63E-05	5.11E-04
**TRINITY_DN302750_c0_g1**	i2	RS24_PONAB	-1.537	1.313	2.83E-03	9.58E-03
**TRINITY_DN33_c0_g1**	i3	RL27_CANLF; RL27_RAT	-1.734	1.372	7.58E-04	3.20E-03
**TRINITY_DN33040_c1_g1**	i4	TYB4_MOUSE	-1.415	0.998	7.52E-03	2.16E-02
**TRINITY_DN353_c0_g1**	i2; i3	RL9_HUMAN; RL9_RAT; RL9_HUMAN; RL9_RAT	-1.322; -2.785	0.643; 1.795	0.016; 2.82E-08	0.039; 4.90E-07
**TRINITY_DN42_c0_g1**	i4	K2C7_BOVIN; K2C7_XENLA	-1.648	0.058	4.29E-03	1.33E-02
**TRINITY_DN4359_c0_g1**	i9	RL23A_RAT	-2.124	0.233	8.99E-05	5.28E-04
**TRINITY_DN45_c0_g1**	i3	RL24_RAT	-1.411	2.164	5.26E-03	1.58E-02
**TRINITY_DN459_c4_g2**	i15; i2	NCBP3_HUMAN	-4.463; -5.303	1.142; 3.754	1.79E-16; 1.93E-25	8.68E-15; 2.60E-23
**TRINITY_DN475_c0_g1**	i13	FRIHB_XENLA	-1.271	2.002	1.22E-02	3.24E-02

**TRINITY_DN5_c0_g1**	i1	S10AB_CHICK	-1.234	1.700	1.63E-02	4.06E-02
**TRINITY_DN5111_c0_g1**	i3	RL13A_HUMAN	-1.646	-0.171	4.32E-03	1.33E-02
**TRINITY_DN5997_c0_g1**	i2	RSSA_CHICK; RSSA_XENLA	-1.506	1.689	3.23E-03	1.07E-02
**TRINITY_DN6930_c0_g1**	i3	ANXA1_CAVCU; ANXA1_RABIT	-1.382	-0.211	1.58E-02	3.98E-02
**TRINITY_DN696_c1_g1**	i1	ARR2_ONCMY; ARRB2_PONAB	-2.500	2.149	6.14E-07	7.27E-06
**TRINITY_DN71_c0_g1**	i1; i3	CXB5_HUMAN	-1.917; -1.958	-0.258; -0.185	0.0008; 0.0005	0.003; 0.002
**TRINITY_DN79_c0_g1**	i13	MUC16_HUMAN	-1.806	-0.063	1.32E-03	5.05E-03
**TRINITY_DN79729_c0_g1**	i3	RS6_XENLA	-1.471	0.276	8.49E-03	2.38E-02
**TRINITY_DN8_c17_g1**	i11	SPAG1_DANRE	-1.949	8.329	5.15E-05	3.32E-04
**TRINITY_DN81_c0_g1**	i7	RS17_COTJA; RS17_PIG	-1.607	0.500	2.82E-03	9.58E-03
**TRINITY_DN81633_c0_g1**	i1	RL17_HUMAN; RL17_RAT	-1.827	-0.018	1.32E-03	5.05E-03
**TRINITY_DN8337_c0_g1**	i3	PDIA1_CHICK; PDIA1_HUMAN	-1.835	0.132	9.23E-04	3.71E-03
**TRINITY_DN878_c0_g1**	i6	H2B1_DANRE	-2.381	2.904	1.19E-06	1.31E-05
**TRINITY_DN94_c0_g1**	i3	H3_URECA; H33_XENTR	-2.198	0.674	3.61E-05	2.50E-04
**TRINITY_DN9741_c0_g1**	i13	GLNA_BOVIN; GLNA_CANLF	-2.050	-0.281	4.12E-04	1.91E-03
**TRINITY_DN981_c0_g2**	i1	CSN2_XENLA; SMC1A_RAT	-1.266	2.019	1.32E-02	3.42E-02

Table S8. Down-regulated annotated genes/proteins when comparing Late vs Norm groups.

ID	Isoforms	Proteins	Log FCs	Log CPMs	P-Value	FDRs

**TRINITY_DN0_c93_g1**	i1	HBB2_TRICR	-1.814	2.756	9.964E-04	2.494E-03
**TRINITY_DN10728_c0_g1**	i16	CAPG_BOVIN	-2.420	0.248	5.650E-05	2.147E-04
**TRINITY_DN10747_c0_g1**	i10	AQP3_BOVIN	-2.624	0.234	1.59E-05	7.310E-05
**TRINITY_DN10760_c0_g1**	i5; i11	HBA1_PLEWA; HBB1_TRICR; HBA1_PLEWA; HBB1_TRICR	-1.718 -3.745	5.173; 6.552	0.001; 1.23E-11	0.003; 3.85E-10
**TRINITY_DN11_c0_g1**	i1	PUF60_BOVIN	-2.166	0.585	3.543E-04	1.050E-03
**TRINITY_DN1109_c0_g1**	i2	RL38_RAT	-2.802	0.794	2.020E-06	1.380E-05
**TRINITY_DN11183_c0_g1**	i2	RL12_HUMAN; RL12_MOUSE	-1.590	1.067	0.006	1.168E-02
**TRINITY_DN112_c1_g2**	i33; i7; i6; i15	PPID_BOVIN; YCX91_PHAAO;	-1.262; -1.273; -1.326 -1.548	3.828; 4.586; 6.874; 2.105	0.020; 0.018; 0.013 0.006	0.033; 0.030; 0.023; 0.011
**TRINITY_DN113_c0_g1**	i32	HMGB3_CHICK	-1.774	-0.234	0.005	9.752E-03
**TRINITY_DN117_c0_g1**	i1	RS3A_CALJA; RS3A_HUMAN; RS3A_XENTR	-1.449	0.482	0.015	2.590E-02
**TRINITY_DN1198_c0_g1**	i24	RACK1_RAT; SMUF1_HUMAN; SMUF1_MOUSE	-1.421	1.509	0.013	2.281E-02
**TRINITY_DN125068_c0_g1**	i1	RL37A_XENLA	-2.143	2.895	9.440E-05	3.368E-04
**TRINITY_DN131_c0_g1**	i16	K2C4_HUMAN; K2C5_PANTR; K2C6A_HUMA; K2C7_XENLA	-2.238	2.658	5.270E-05	2.030E-04
**TRINITY_DN13545_c0_g2**	i9	RL32_BOVIN; RL32_RAT	-1.479	0.408	0.014	2.448E-02
**TRINITY_DN1501_c0_g1**	i1	RS15A_PONAB; RS15A_RAT	-1.907	3.812	4.393E-04	1.250E-03
**TRINITY_DN15564_c0_g2**	i13	AAKG1_HUMAN; G3P_CHICK; G3P_FELCA	-1.609	1.096	0.006	1.069E-02
**TRINITY_DN16127_c0_g1**	i12	DAA1A_XENLA	-1.770	4.867	1.012E-03	2.523E-03
**TRINITY_DN167_c0_g1**	i11; i20; i42; i25; i37; i48; i2	MUC5A_HUMAN;	-1.395; -1.528; -1.617; -1.829; -1.845; -2.134; -2.478	3.266; 2.550; -0.0403; 1.144; 1.4534; 3.631; 2.494	0.011; 0.005; 0.009; 0.001; 0.001; 8.76E-05; 7.68E-06	0.019; 0.010; 0.017; 0.004; 0.003; 0.0003; 4.13E-05
**TRINITY_DN17650_c0_g1**	i3	RS12_CHICK; RS12_PIG	-1.335	1.331	0.020	3.250E-02
**TRINITY_D**	i16	MUC2_HUMAN;	-2.128	0.029	5.737E-04	1.567E-03

**N18788_c0_g 3**		MUC5B_CHICK; VWF_CANLF				
**TRINITY_D N1909_c0_g1**	i1	RL30_OPHHA	-2.566	2.720	3.340E-06	2.060E-05
**TRINITY_D N210791_c0_ g1**	i2; i1	RL37_RAT	-1.598; -2.903	1.783; 3.228	0.004; 1.57E-07	0.008; 1.49E-06
**TRINITY_D N222_c0_g1**	i55	TC3A_CAEEL	-2.377	1.367	2.800E-05	1.185E-04
**TRINITY_D N22624_c0_g 1**	i11	ADT2_MOUSE; ADT2_TACAC	-1.993	1.879	4.076E-04	1.168E-03
**TRINITY_D N23068_c0_g 1**	i2	ACOD_CYPCA; SCD1_TACFU	-1.706	0.387	5.326E-03	1.018E-02
**TRINITY_D N246_c0_g1**	i20	EF2_CALJA; EF2_CHICK; EF2_MOUSE; EF2_RAT	-1.917	1.556	6.795E-04	1.800E-03
**TRINITY_D N25_c0_g1**	i3	EIF1_PONAB; EIF1B_PIG	-1.756	0.396	3.613E-03	7.358E-03
**TRINITY_D N27074_c0_g 1**	i9	EHF_MOUSE; MUC5A_HUMA N; MUC5B_HUMA N	-2.001	-0.335	3.242E-03	6.713E-03
**TRINITY_D N302750_c0_ g1**	i2	RS24_PONAB	-3.395	1.144	7.460E-09	1.130E-07
**TRINITY_D N3052_c0_g1**	i3	RL26_MOUSE; RL26L_HUMAN	-1.353	2.239	0.015	2.590E-02
**TRINITY_D N321_c0_g1**	i2	LDHA_AMBME; LDHA_RABIT	-1.777	-0.034	3.729E-03	7.519E-03
**TRINITY_D N353_c0_g1**	i2; i3	RL9_HUMAN; RL9_RAT; RL9_HUMAN; RL9_RAT	-2.086; -2.784	0.681; 2.326	0.0004; 8.46E-07	0.001; 6.55E-06
**TRINITY_D N3702_c0_g1**	i9	ASSY_RAT; ASSY_XENLA; ASSY_XENTR	-1.668	1.526	3.602E-03	7.347E-03
**TRINITY_D N4359_c0_g1**	i9; i3	RL23A_RAT	-1.463; -1.577	0.9007; 1.185	0.013; 0.006	0.023; 0.011
**TRINITY_D N4415_c0_g1**	i8	RL3_HUMAN; RL3_PIG; RL3_RAT	-1.621	0.509	8.000E-03	1.478E-02
**TRINITY_D N45_c0_g1**	i3	RL24_RAT	-3.308	2.006	1.120E-08	1.540E-07
**TRINITY_D N459_c4_g2**	i8; i2; i15	NCBP3_HUMAN	-2.354; -3.074; -3.513	1.653; 4.688; 1.836	2.87E-05; 1.92E-08; 1.07E-09	0.0001; 2.47E-07; 2.26E-08
**TRINITY_D N475_c0_g1**	i2; i13	FRIHB_XENLA	-1.633; -4.384	2.883; 1.592	0.003; 4.01E-13	0.006; 1.75E-11
**TRINITY_D N4752_c0_g1**	i9	HOOK3_HUMA N; RS20_XENLA	-1.367	0.764	0.020	3.255E-02
**TRINITY_D N49798_c0_g 3**	i5	RL5_CHICK; RL5_MACFA	-2.208	0.570	3.543E-04	1.050E-03
**TRINITY_D N5_c0_g1**	i1	S10AB_CHICK	-2.002	1.774	3.800E-04	1.108E-03
**TRINITY_D N55_c0_g1**	i2	RL23_RAT	-1.448	0.327	0.018	3.030E-02

**TRINITY_DN56_c0_g1**	i3	EF1D_RAT; EF1D_XENLA	-2.118	-0.069	6.964E-04	1.833E-03
**TRINITY_DN59473_c0_g1**	i9	RL34_ICTPU; RL34_PIG	-1.585	2.233	0.004	8.282E-03
**TRINITY_DN6026_c0_g1**	i10	TSP1_XENLA	-2.795	0.340	1.350E-05	6.490E-05
**TRINITY_DN62_c0_g1**	i2	HBB2_TRICR	-2.240	1.512	8.780E-05	3.151E-04
**TRINITY_DN6230_c0_g4**	i2	RL39_CHICK	-2.563	1.177	9.650E-06	4.940E-05
**TRINITY_DN668_c1_g1**	i28	MUC5A_HUMAN; MUC5B_HUMAN	-1.632	-0.046	8.040E-03	1.451E-02
**TRINITY_DN696_c1_g1**	i1	ARR2_ONCMY; ARRB2_PONAB	-2.717	2.611	1.090E-06	8.160E-06
**TRINITY_DN7354_c0_g2**	i8	RS13_GILMI; RS13_XENLA	-2.519	0.160	3.910E-05	1.590E-04
**TRINITY_DN75_c0_g1**	i2	RL21_MOUSE; RL21_PIG; RL21_RAT	-1.734	1.783	1.956E-03	4.411E-03
**TRINITY_DN78434_c0_g1**	i1	RL36_HYDHA	-1.452	1.340	1.145E-02	2.003E-02
**TRINITY_DN79_c0_g1**	i13	MUC16_HUMAN	-1.372	0.484	2.55E-02	4.06E-02
**TRINITY_DN8_c17_g1**	i21; i9; i22; i1; i11	SPAG1_DANRE	-1.385; -1.515; -1.562; -1.617; -3.483	1.599; 5.053; 9.805; 14.177; 8.482	0.015; 0.005; 0.003; 0.002; 2.10E-10	0.025; 0.009; 0.007; 0.005; 5.15E-09
**TRINITY_DN80_c1_g1**	i15; i23	ACT5_XENLA; ACTA_RAT; ACTB_TRIVU; ACTB_XENLA; ACTB_XENTR; ACTH_RAT; ACTS_RAT	-1.684; -1.766	-0.281; 0.720	0.008; 0.003	0.014; 0.006
**TRINITY_DN8337_c0_g1**	i3	PDIA1_CHICK; PDIA1_HUMAN	-2.068	0.403	5.697E-04	1.564E-03
**TRINITY_DN84_c0_g1**	i3	RS7_RAT	-1.228	1.728	3.101E-02	4.835E-02
**TRINITY_DN878_c0_g1**	i6	H2B1_DANRE	-1.572	3.717	3.701E-03	7.496E-03
**TRINITY_DN94_c0_g1**	i3	H33_XENTR; H3_URECA	-3.465	0.757	9.690E-09	1.380E-07
**TRINITY_DN955_c1_g2**	i11	CC127_HUMAN	-7.906	8.533	7.470E-35	4.570E-32
**TRINITY_DN972_c0_g1**	i11	ANXA1_CHICK; ANXA1_HORSE; ANXA1_HUMAN; ANXA1_RABIT	-1.841	-0.026	3.264E-03	6.725E-03

List 1. RNA-binding Up-regulated proteins in early stage (a) and late stage (b) samples.

(a) EIF4A1; eEF1a1; TPT1; CAVIN1; GANAB; EPPK1; nap1l1; FUS; DEK; Luc7l3; Habp4; EIF2S2; SF3A3; DDX5; HMGB3; PDIA3; CANX; CAPRIN1; RPL22; KTN1; PRRC2C; HMGB2; PUF60

(b) AHNAK, AKAP8L, ANP32A, CALR, CANX, CAPRIN1, CAST, CLU, CSDE1, CTNNA1, DEK, DHX15, DSP, EEF2, EIF2S2, EIF3L, EIF4A1, EIF4G2, FUS, GANAB, Gtpbp4, Habp4, HMGB2, HMGB3, HSP90B1, HSPA8, KTN1, Lrrfip1, Luc7l3, Myh10, nap1l1, NCBP3, NOL8, NSA2, PDIA3, PLEC, PPIB, Ppme1, PRPF38A, PRRC2C, PUF60, PUM1, R3HDM1, RPL22, Rpl23, Rps9, Rtf1, SAFB, SF3A3, Sin3a, SSB, STAU1, Tmsb4x, tp53, TPT1, ZNF326, ZNFX1,

List 2. RNA-binding Down-regulated proteins in early stage (a) and late stage (b) samples

(a) RPL12, Rpl23, Rpl23a, RPL3, Rpl37, RPL5.
(b) RPL9EEF2, h4c1, NCBP3, P4HB, PUF60, Rack1, RPL13A, RPL17, Rpl23a, Rpl24, RPL27, Rpl31, Rpl37, Rpl38, RPL9, RPS12, RPS17, RPS24, Slc25a5, Tmsb4x.

Table S9. Signaling cascades enriched by up-regulated proteins.

Data Base	Name	Overlap	P-value	Adjusted P-value	Odds Ratio	Genes
**Reactome 2022**	Calnexin/calreticulin Cycle R-HSA-901042	4/26	3.50E-05	0.0148	25.57	PDIA3; GANAB; CANX; CALR
**Reactome 2022**	Muscle Contraction R-HSA-397014	8/196	9.34E-05	0.0148	6.11	RYR1; ANXA1; TPM1; AKAP9; MYH11; NEB; ATP2A1; ATP1B1
**Reactome 2022**	Maturation Of Spike Protein R-HSA-9683686	2/23	5.15E-04	0.0330	92.55	GANAB; CANX
**Reactome 2022**	N-glycan Trimming In ER And Calnexin/Calreticulin Cycle R-HSA-532668	4/35	1.17E-04	0.0148	18.14	PDIA3; GANAB; CANX; CALR
**Reactome 2022**	L13a-mediated Translational Silencing Of Ceruloplasmin Expression R-HSA-156827	6/108	1.36E-04	0.0148	8.36	EIF4A1; RPS9; EIF3L; RPL23; RPL22; EIF2S2
**Reactome 2022**	GTP Hydrolysis And Joining Of 60S Ribosomal Subunit R-HSA-72706	6/109	1.44E-04	0.0148	8.28	EIF4A1; RPS9; EIF3L; RPL23; RPL22; EIF2S2
**Reactome 2022**	Cap-dependent Translation Initiation R-HSA-72737	6/116	2.02E-04	0.0174	7.75	EIF4A1; RPS9; EIF3L; RPL23; RPL22; EIF2S2
**Reactome 2022**	mRNA Activation Upon Binding Of Cap-Binding Complex And eIFs Subsequent Binding To 43S R-HSA-72662	4/58	8.30E-04	0.0330	10.40	EIF4A1; RPS9; EIF3L; EIF2S2
**Reactome 2022**	Ribosomal Scanning And Start Codon Recognition R-HSA-	4/56	7.77E-04	0.0330	10.60	EIF4A1; RPS9; EIF3L; EIF2S2

	72702					
**Reactome 2022**	Translation Initiation Complex Formation R-HSA-72649	4/57	7.77E-04	0.0330	10.60	EIF4A1; RPS9; EIF3L; EIF2S2
**Reactome 2022**	Virus Assembly And Release R-HSA-168268	2/23	7.68E-04	0.0330	69.41	CANX; CALR
**Reactome 2022**	Cellular Responses to Stress R-HSA-2262752	14/722	7.83E-04	0.0330	2.89	TSPYL2; HSPA8; ATP6V1G1; RPS9; RPL23; RPL22; EIF2S2; HSP90B1; PSMB7; CCS; SIN3A; CALR; TP53; SKP1
**Reactome 2022**	Cellular Responses to Stimuli R-HSA-8953897	14/736	9.42E-04	0.0348	2.83	TSPYL2; HSPA8; ATP6V1G1; RPS9; RPL23; RPL22; EIF2S2; HSP90B1; PSMB7; CCS; SIN3A; CALR; TP53; SKP1
**Reactome 2022**	Antigen Presentation: Folding Assembly Peptide Loading Of Class I MHC R-HSA-983170	3/28	0.001071	0.0369	16.76	PDIA3; CANX; CALR
**BioPlanet 2019**	N-glycan trimming in the ER and calnexin/calreticulin cycle	4/13	1.80E-06	6.8E-04	62.56	PDIA3; GANAB; CANX; CALR
**BioPlanet 2020**	Eukaryotic protein translation	4/17	5.86E-06	0.0011	43.30	EIF4A1; EEF2; EIF2S2; EIF4G2
**BioPlanet 2021**	Protein processing in the endoplasmic reticulum	8/166	2.89E-05	0.0036	7.28	PDIA3; HSPA8; GANAB; CANX; CALR; SEC62; SKP1; HSP90B1
**BioPlanet 2022**	ER-associated degradation (ERAD) pathway	3/19	3.32E-04	0.0273	26.20	GANAB; CANX; SKP1
**BioPlanet 2023**	Golgi-associated vesicle biogenesis	3/20	3.89E-04	0.0273	24.65	NAPA; HSPA8; PUM1
**BioPlanet 2024**	Muscle contraction	4/49	4.36E-04	0.0273	12.49	TPM1; MYH11; NEB; TTN
**BioPlanet 2025**	Apoptotic execution phase	4/52	5.48E-04	0.0294	11.71	DSP; TJP1; HMGB2; PLEC
**BioPlanet 2026**	Translation	6/151	8.24E-04	0.0387	5.87	EIF4A1; RPS9; RPL23; RPL22; EEF2; EIF2S2
**BioPlanet 2027**	Internal ribosome entry pathway	2/7	0.00107	0.0447	55.52	EIF4A1; EIF4G2

**KEGG2021 Human**	Protein processing in endoplasmic reticulum	8/171	3.57E-05	0.0058	7.05	PDIA3; HSPA8; GANAB; CANX; CALR; SEC62; SKP1; HSP90B1
**KEGG2021 Human**	Pathways of neurodegeneration	12/475	1.83E-04	0.0147	3.78	RYR1; PSMB7; CCS; FUS; NDUFA4; ATP6; LRP5; COX2; ATP2A1; KLC1; ND2; PPID
**KEGG2021 Human**	Spliceosome	6/150	7.96E-04	0.0279	5.91	SF3A3; HSPA8; PRPF38A; PUF60; FUS; DHX15
**KEGG2021 Human**	Antigen processing and presentation	4/78	0.0025	0.0446	7.58	PDIA3; HSPA8; CANX; CALR
**KEGG2021 Human**	Oxidative phosphorylation	5/133	0.0029	0.0460	5.50	ATP6V1G1; NDUFA4; ATP6; COX2; ND2

Table S10. Signaling cascades enriched by down-regulated proteins.

Data Base	Name	Overlap	P-value	Adjusted P-value	Odds Ratio	Genes
Reactome 2022	rRNA Processing R-HSA-72312	11/199	3.12E-13	6.48E-12	36.15	RPS17; RPL31; RPL13A; RPL38; RPL23A; RPL27; RPL37; RPL9; UBA52; RPS24; RPS12
Reactome 2022	Translation R-HSA-72766	12/281	4.87E-13	9.65E-12	28.33	RPS17; RPL31; RPL13A; RPL38; RPL23A; RPL27; RPL37; EEF2; RPL9; UBA52; RPS24; RPS12
Reactome 2022	Translation Initiation Complex Formation R-HSA-72649	3/57	2.50E-04	0.002866	27.64	RPS17; RPS24; RPS12
Reactome 2022	Termination Of O-glycan Biosynthesis R-HSA-977068	2/25	0.0013	0.0133	42.28	MUC2; MUC16
Reactome 2022	Regulation Of TNFR1 Signaling R-HSA-5357905	2/35	0.0026	0.0249	29.45	RACK1; UBA52
Reactome 2022	C-type Lectin Receptors (CLRs) R-HSA-5621481	3/141	0.0034	0.0320	10.77	MUC2; MUC16; UBA52
Reactome 2022	TNF Signaling R-HSA-75893	2/44	0.0040	0.0366	23.13	RACK1; UBA52

BioPlanet 2019	Cytoplasmic ribosomal proteins	13/108	5.03E-20	5.25E-18	90.60	RPL31; RPL13A; RPL23A; RPL9; RPS17; RPL24; RPL38; RPL27; RPL37; RPL17; UBA52; RPS24; RPS12
BioPlanet 2019	Translation	14/151	6.86E-20	5.25E-18	69.84	RPL31; RPL13A; RPL23A; EEF2; RPL9; RPS17; RPL24; RPL38; RPL27; RPL37; RPL17; UBA52; RPS24; RPS12
BioPlanet 2019	Termination of O-glycan biosynthesis	2/26	0.00142	0.021728	40.51	MUC2; MUC16
WikiPathways	Cytoplasmic Ribosomal Proteins WP477	13/88	3.01E-21	1.54E-19	114.87	RPL31; RPL13A; RPL23A; RPL9; RPS17; RPL24; RPL38; RPL27; RPL37; RPL17; UBA52; RPS24; RPS12
WikiPathways	VEGFA VEGFR2 Signaling WP3888	5/430	0.0022	0.0562	6.05	TMSB4X; RACK1; RPL13A; RPL27; P4HB

Figure S1. Up-regulated protein classes from early and late stage of retina regeneration.

Frequency indicators are presented in absolute terms.

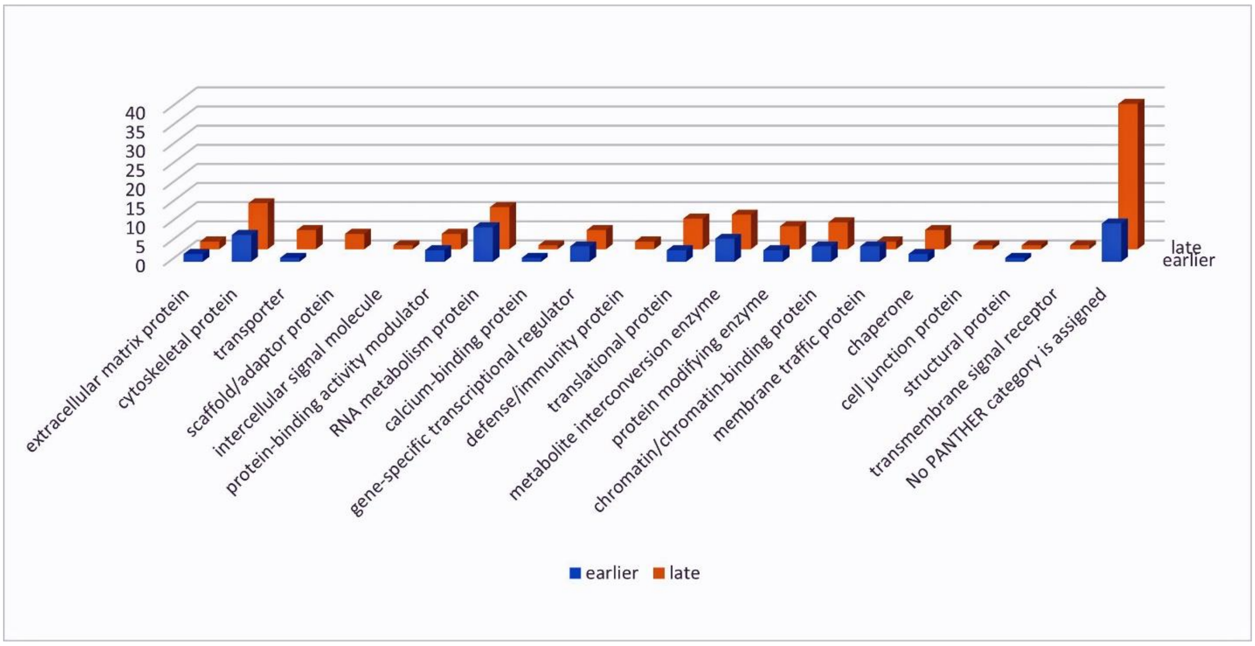

Text 1. Assessment of the composition of up-regulated proteins at early and later stages of regeneration according to the PANTHER Classification System.

The analysis was carried out according to the “PANTHER GO-Slim Molecular Function” protein classification presented in the PANTHER Classification System. This classification is more detailed includes widely used classes of protein function, and contains a larger number of classes. At the same time, the size of the resulting classes is on average smaller than the size of GO classes, which reduces the estimate of the probability of a non-random inclusion of the target protein in a particular class.

The diagram shows 20 classes of up-regulated genes, including a group of unclassified genes shown for the stages of retinal regeneration studied (**Figure S1**). One can note a high proportion and/or continued increase at a later stage of regeneration of cytoskeletal proteins, RNA metabolism proteins, metabolite interconversion enzymes and chromatin/chromatin-binding proteins. The classes transporters, scaffold/adaptor proteins, protein modifying enzyme and chaperones are represented by rare up-regulated genes at the early stage of regeneration and double their numbers at a later stage. Confirmation of significant enrichment of classes of up-regulated genes using Fisher's Exact test and FDR<0.05 was not obtained for both stages of regeneration, due to the small number of classified proteins. However, based on the Fold Enrichment (FE) and raw P-value (p) indicators, we can identify a significant excess of the expected level of up-regulated genes in the glucosidases group (FE 38.13, p 0.029) as part of the metabolite interconversion enzyme, intermediate filament class binding protein (FE 24.51, p 0.043) and actin (FE 6.15, p 0.001) as part of the cytoskeletal protein class, HMG box transcription factor (FE 20.19, p 0.049) as part of the class as part of the gene-specific transcriptional regulators class, RNA helicase (FE 9.53, p 0.019) as part of the RNA metabolism protein class, translation factor class (FE 6.07, p 0.044).

The later stage of RPE reprogramming during retinal regeneration in the newt is characterized by an increase in the number of classes with up-regulated genes. They are: the amino-acid kinase as part of protein modifying enzyme (FE 41.18, p 0.024), cytoskeletal protein (FE 3.10, p 5.32E-04) and their constituent intermediate filament binding protein (FE 23.53, p 0.003), P53-like transcription factor (FE 23.53, p 0.041) as part of chromatin/chromatin-binding proteins, Hsp90 family chaperone (FE 20.59, p 0.047) and the entire class of chaperones (FE 4.00, p 0.008), phosphatase inhibitor (FE 20.59, p 0.047) in as part of the class protein-binding activity modulator, HMG box transcription factors (FE 9.69, p 0.018) as part of the class gene-specific transcriptional regulators. Changes in FE in the range from 8 to 5 are shown for the classes of translational protein, RNA metabolism protein, ATP synthase as part of the transporter class.

Figure S2. Down-regulated protein classes from earlier and late stage of retina regeneration.
Frequency indicators are presented in absolute terms.

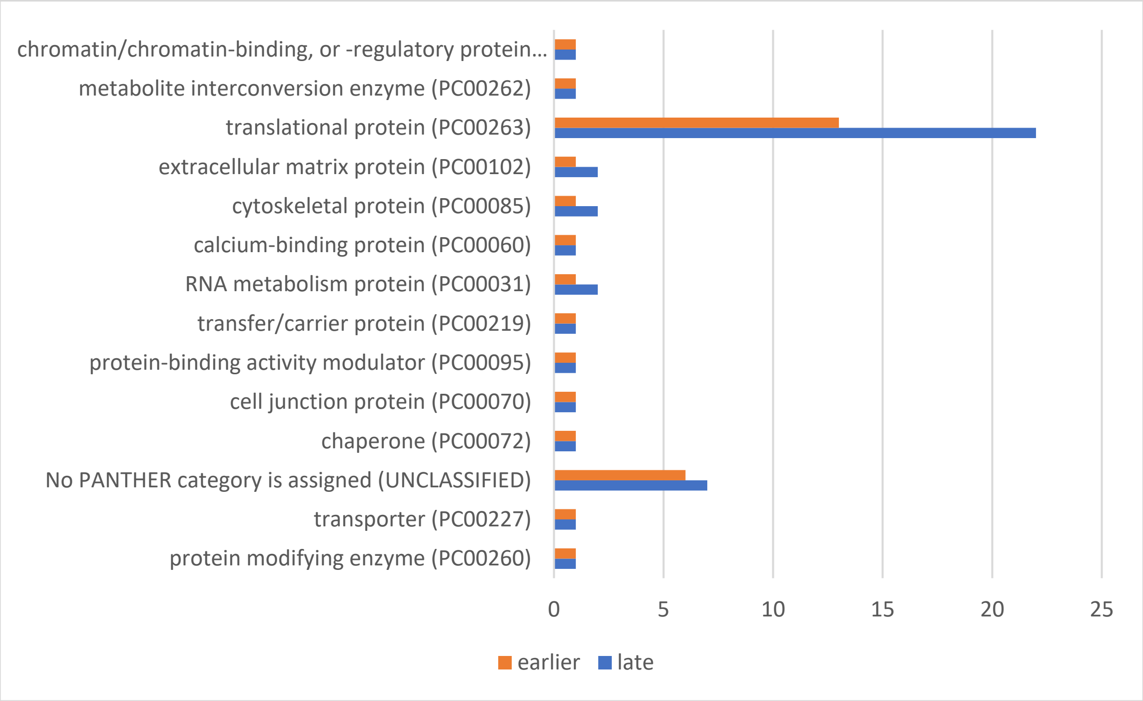

Pipeline for processing source data in appropriate programs.

Cod 1.txt

```
Trinity --seqType fq --left
../2/2_1.fq.gz,../3/3_1.fq.gz,../4/4_1.fq.gz,../5/5_1.fq.gz,../6/6_1.fq.gz,../7/7_1.fq.gz
    --right ../2/2_2.fq.gz,../3/3_2.fq.gz,../4/4_2.fq.gz,../5/5_2.fq.gz,../6/6_2.fq.gz,../7/7_2.fq.gz,
    --CPU 16 --max_memory 100G --min_contig_length 150  --output /var/bioinf/ref/
```

Cod 2.txt

```
TransDecoder.LongOrfs -t trinity_out.Trinity.fasta

blastp -db trinity_out.Trinity.fasta.transdecoder_dir/longest_orfs.pep -out axolotl_blast.tblout -
evalue 1e-05 -max_target_seqs 1 -outfmt 6 -num_threads 8

hmmscan --cpu 7 --domtblout axolotl_pfam.domtblout dbs/Pfam-A.hmm
trinity_out.Trinity.fasta.transdecoder_dir/longest_orfs.pep
Pfam-A.hmm trinity_out.Trinity.fasta.transdecoder_dir/longest_orfs.pep


TransDecoder.Predict -t trinity_out.Trinity.fasta  --retain_blastp_hits axolotle3_blast.tblout --
retain_pfam_hits axolotl_pfam.domtblout
```

Cod 3.txt

```
cd-hit -M 6000 -d 0 -T 2 -i trinity_out.Trinity.fasta.transdecoder.pep -o
axolotle.Trinity.fasta.transdecoder.pep.nr90 -g 1 -c 0.90 -b 1000
```

Cod 4.txt

```
busco/bin/busco -c 2 -i axolotle.Trinity.fasta.transdecoder.pep.nr90 -o BUSCO-axolotle-euk -l eukaryota_odb10 -m prot
busco/bin/busco -c 2 -i axolotle.Trinity.fasta.transdecoder.pep.nr90 -o BUSCO-axolotle-bac -l bacteria_odb10 -m prot
```

Cod 5.txt

For each of the samples (where {x} is the sample number: 2,3,4,5,6,7):

```
align_and_estimate_abundance.pl --seqType fq --left {x}/{x}_1.fq.gz --right {x}/{x}_2.fq.gz --transcripts trinity_out_dir.Trinity.fasta --thread_count 16 --est_method RSEM --output_dir {x}/ --aln_method bowtie2 --trinity_mode --coordsort_bam --prep_reference

abundance_estimates_to_matrix.pl --est_method RSEM --out_prefix matrix_trans2 --name_sample_by_basedir --gene_trans_map none 2/RSEM.isoforms.results 3/RSEM.isoforms.results 4/RSEM.isoforms.results 5/RSEM.isoforms.results 6/RSEM.isoforms.results 7/RSEM.isoforms.results

run_DE_analysis.pl --matrix matrix_trans2.isoform.counts.matrix --samples_file samples_all.txt --method edgeR --dispersion 0.1 --output edgeR_trans_all

analyze_diff_expr.pl --matrix matrix_trans2.isoform.counts.matrix --samples samples_t.txt -P 1e-3 -C 2

cd edgeR_trans_all
analyze_diff_expr.pl --matrix matrix_trans2.isoform.TMM.EXPR.matrix --samples samples_all.txt -P 1e-3 -C 2
```

Cod 6.txt

```
define_clusters_by_cutting_tree.pl -R diffExpr.P1e-3_C2.matrix.RData --Ptree 60
```

Cod 7.txt

```
extract_GO_assignments_from_Trinotate_xls.pl \
                        --Trinotate_xls trinotate.xls \
                        -G --include_ancestral_terms \
                        > go_annotations.txt

extract_GO_assignments_from_Trinotate_xls.pl \
                        --Trinotate_xls trinotate.xls \
                        -G --include_ancestral_terms \
                        > go_annotations.txt

blastx -query ref/trinity_out_dir.Trinity.fasta -db Trinotate-Trinotate-v3.2.2/admin/uniprot_sprot.pep -out blastx.outfmt6 -evalue 1e-5 -num_threads 16 -max_target_seqs 1 -outfmt 6

blastp -query td/trinity_out_dir.Trinity.fasta.transdecoder.pep -db Trinotate-Trinotate-v3.2.2/admin/uniprot_sprot.pep -num_threads 12 -max_target_seqs 1 -outfmt 6 -evalue 1e-5 > blastp.outfmt6
```
